# Fate of the RAFT End-Group in the Thermal Depolymerization of Polymethacrylates

**DOI:** 10.1021/acsmacrolett.3c00418

**Published:** 2023-08-24

**Authors:** Florian Häfliger, Nghia P. Truong, Hyun Suk Wang, Athina Anastasaki

**Affiliations:** † Laboratory of Polymeric Materials, Department of Materials, ETH Zurich, Vladimir-Prelog-Weg 5, 8093 Zurich, Switzerland; ‡ Monash Institute of Pharmaceutical Sciences, Monash University, 399 Royal Parade, Parkville, VIC 3152, Australia

## Abstract

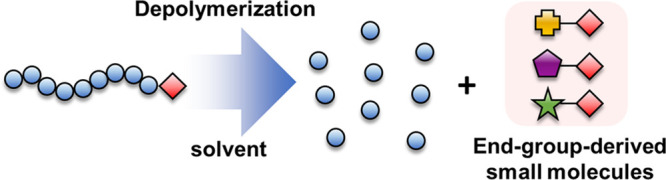

Thermal RAFT depolymerization has recently emerged as a promising methodology for the chemical recycling of polymers. However, while much attention has been given to the regeneration of monomers, the fate of the RAFT end-group after depolymerization has been unexplored. Herein, we identify the dominant small molecules derived from the RAFT end-group of polymethacrylates. The major product was found to be a unimer (DP = 1) RAFT agent, which is not only challenging to synthesize using conventional single-unit monomer insertion strategies, but also a highly active RAFT agent for methyl methacrylate, exhibiting faster consumption and yielding polymers with lower dispersities compared to the original, commercially available 2-cyano-2-propyl dithiobenzoate. Solvent-derived molecules were also identified predominantly at the beginning of the depolymerization, thus suggesting a significant mechanistic contribution from the solvent. Notably, the formation of both the unimer and the solvent-derived products remained consistent regardless of the RAFT agent, monomer, or solvent employed.

Reversible deactivation radical polymerization (RDRP) allows the synthesis of polymers with well-defined end-groups, offering high control over molecular weight and dispersity and enabling the synthesis of block copolymers.^1−11^ The primary principle of RDRP is the frequent deactivation of propagating chains that ultimately results in chains that are terminated by a chemical moiety, typically a halogen or thiocarbonylthio compound.^2,12,13^ For example, in reversible addition–fragmentation chain-transfer (RAFT) polymerization, chains are terminated by a thiocarbonylthio chain-transfer agent, while atom transfer radical polymerization (ATRP) generates chain-ends with a halogen group.^13^

Recently, polymethacrylates synthesized by various RDRP methodologies have been actively utilized for depolymerization to monomer, exploiting their activatable end-groups, which provide a low-temperature route to introducing a chain-end radical that can trigger depolymerization.^14−29^ Polymethacrylates with halogen chain ends have been depolymerized by several groups, usually in the presence of a catalyst that abstracts the halogen end-group.^19,21,22,24,28^ For example, Matyjaszewski and co-workers recently demonstrated the depolymerization of poly(methyl methacrylate) (PMMA) and poly(*n*-butyl methacrylate) using an iron catalyst, reaching an impressive 70% depolymerization at 170 °C.^22^ In the RAFT arena, depolymerization of polymethacrylates with thiocarbonylthio end-groups was reported by our group and the groups of Gramlich^25^ and Sumerlin.^23,29^ For instance, we recently reported the thermal depolymerization of dithiobenzoate-terminated polymethacrylates in dioxane, reaching up to 92% conversion at 120 °C, and later expanded the scope to various end-groups and solvents.^14,17^ Sumerlin and co-workers reported an elegant photoassisted depolymerization of PMMA, whereby visible light irradiation enabled a reduction of the reaction temperature.^23^ In parallel, our group reported a photoaccelerated depolymerization of PMMA in the presence of a photocatalyst.^15^

Despite these advances in reversing RDRP, identifying the end-group-derived small molecule products obtained after depolymerization has received little attention (Figure 1). This is a significant omission in the literature, as the end-group is the linchpin for depolymerization of RDRP-synthesized polymethacrylates. Especially, for thermal polymerizations, determining the end-group after depolymerization may provide critical mechanistic information. At the same time, considering that the RAFT agent is an expensive reagent in RAFT polymerization, identifying the RAFT agent retrieved after depolymerization is also important.

**Figure 1:**
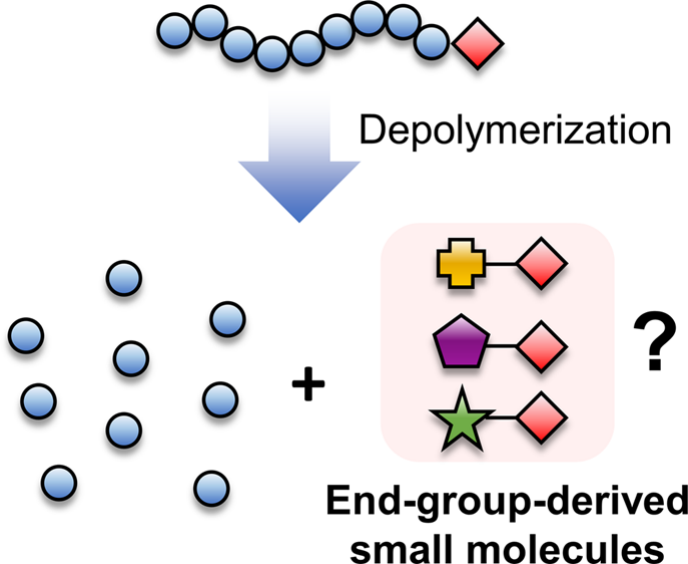
Schematic illustration of the end-group-derived small molecules formed in the depolymerization of RAFT-polymethacrylates.

To initiate our study, PMMA was first synthesized via RAFT polymerization using 2-cyano-2-propyl dithiobenzoate (DTB) as the chain-transfer agent (CTA), yielding a well-defined polymer terminated by a dithiobenzoate unit (Figures S1 and S2 and Table S1). The PMMA-DTB polymer was then thermally depolymerized in 1,4-dioxane at 120 °C (Table S2) and the reaction was monitored by size-exclusion chromatography (SEC) and ^1^H NMR (Figure 2a–c). UV-SEC traces showed a gradual disappearance of the UV signal of the polymer and a gradual appearance of apparently two peaks at higher retention times (>33 min) corresponding to the small-molecule regime (<1000 g/mol), indicating a formation of UV-active products presumed to be derived from the DTB end-group (Figure 2b). ^1^H NMR analysis also showed the appearance of new peaks in the 7.9–8.1 ppm region that suggested structural modifications near the phenyl ring of the dithiobenzoate (Figure 2c). After 6 h, the reaction was stopped and the end-group derived products were purified by flash column chromatography, which visibly showed three major pinkish-red products (Figure S3). The molecular structures of these products were then elucidated using various NMR mass spectrometry techniques (Figures S4–S21) and are depicted in Figure 2d–f. Overall, the three products can be classified into two categories: a unimer (**1**) (i.e., “PMMA-DTB” with a single MMA unit) and a solvent-derived dithiobenzoate (**2**, **3**) (Figure 2b–d). Compound **1** is likely to have formed from the “unzipping” of the PMMA radical until the final MMA unit, and subsequent deactivation by a dithiobenzoate. This is also evidence that deactivation may occur during the depolymerization. We could not identify in any meaningful amount the original RAFT agent (2-cyano-2-propyl dithiobenzoate) as one of the depolymerization products, suggesting that the final MMA unit attached to the 2-cyano-2-propyl group is quite stable from further unzipping. The formation of **1** as the major product is particularly interesting, as previous works have reported the difficulty in synthesizing unimers of nonbulky methacrylates using single-unit monomer insertion^30−32^ due to their tendency to form oligomers.^31,33^ Thus, depolymerization provides a new route for the synthesis of unimers.

**Figure 2:**
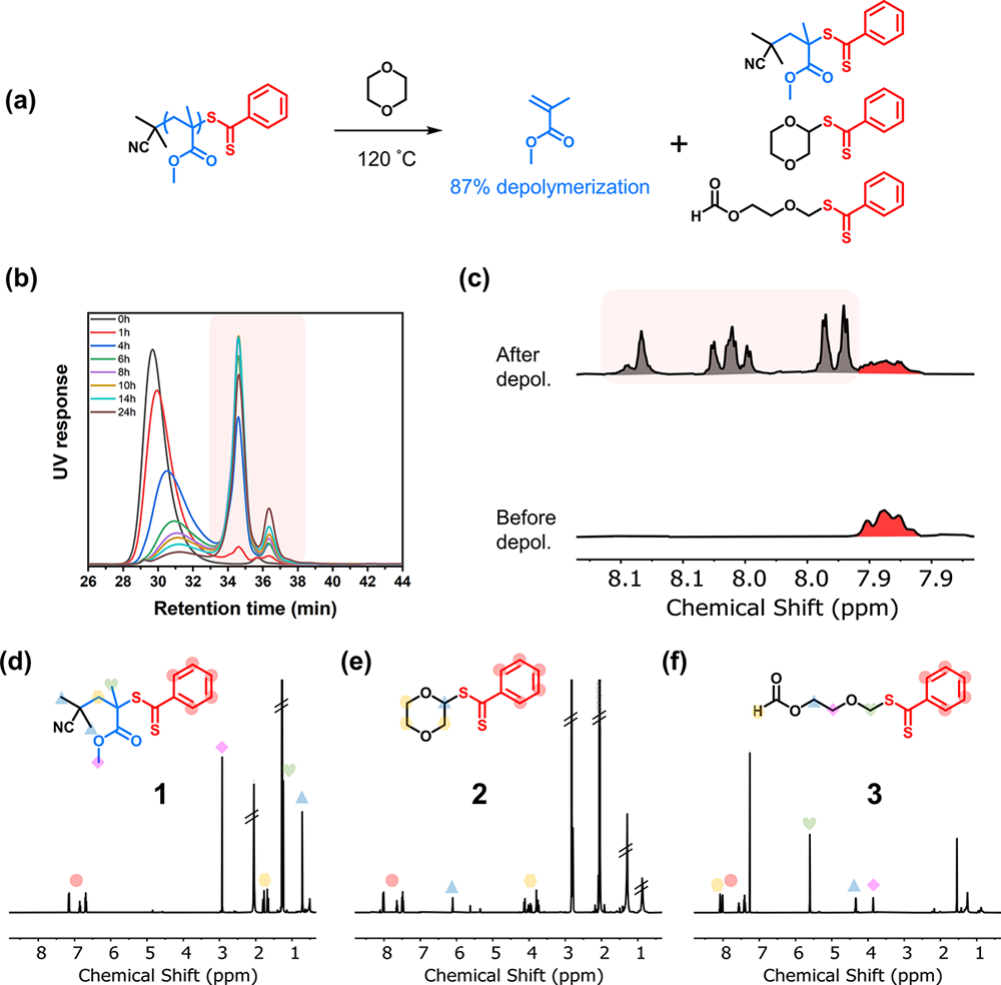
(a) Scheme of PMMA-DTB depolymerized at 120 °C in 1,4-dioxane. (b) UV-SEC traces and (c) magnified ^1^H NMR spectrum of samples during the depolymerization. (d–f) Chemical structure and ^1^H NMR spectra of end-group-derived products after depolymerization.

Compound **2** resembles a 1,4-dioxane-derived dithiobenzoate species, likely formed by the reaction between a carbon-centered 1,4-dioxane radical and PMMA-DTB. The presence of this structure suggests a contribution of an unknown initiation pathway through a solvent radical, which displaces the PMMA in PMMA-DTB by creating a new dioxane-DTB and a PMMA radical that can undergo depropagation. As it is widely documented in the literature that 1,4-dioxane contains trace amounts of peroxides, we hypothesize that the solvent radicals are formed by trace peroxides upon heating (Figure S22).^34−36^ Compound **3** contains a methoxyethyl formate ester fragment connected to the dithiobenzoate through a C–S bond and is also likely to have formed through a similar pathway involving trace peroxides and a ring-opening reaction (Figure S22).^36^ The formation of compounds **2** and **3** is similar to previous reports on the recovery of various dithiobenzoate end-groups by reaction with different free radical sources.^37^

Important pieces of information about thermal depolymerization can be extracted from these identified molecules. The presence of unimer **1** is a direct product of the depolymerization of PMMA-DTB, while the solvent-derived species **2** and **3** are products of the reaction between PMMA-DTB and the solvent radicals. With this in mind, we were interested in investigating the relative generation of compounds **1**, **2**, and **3** during the depolymerization of PMMA-DTB in an effort to shed light on the initiation mechanism of thermal depolymerization.

To investigate the relative fractions of **1**, **2**, and **3** during depolymerization in 1,4-dioxane at 120 °C, we sampled the reaction at various time points and measured the relative intensity of the characteristic proton signals of each product (Figures 3 and S23 and Table S3). We could identify three regimes in the evolution of these products. In the first regime, arbitrarily assigned to be 0–10% depolymerization, **2** and **3** comprised 80% of the small-molecule dithiobenzoate products, and only 20% of **1** was detected. As **2** and **3** are most likely products of initiation by solvent and **1** is a product of chain depolymerization, the presence of such a large fraction of **2** and **3** is very strong evidence that a significant initiation pathway occurs through solvent radicals. It is worth noting that this does not rule out the presence of direct homolytic cleavage of the end-group (C–S homolysis). The small relative fraction of the unimer was attributed to partial deactivation occurring at the beginning of the depolymerization, thus delaying the unimer formation. This hypothesis was supported by the SEC traces shifting to a lower molecular weight at the early stages of the reaction.

**Figure 3:**
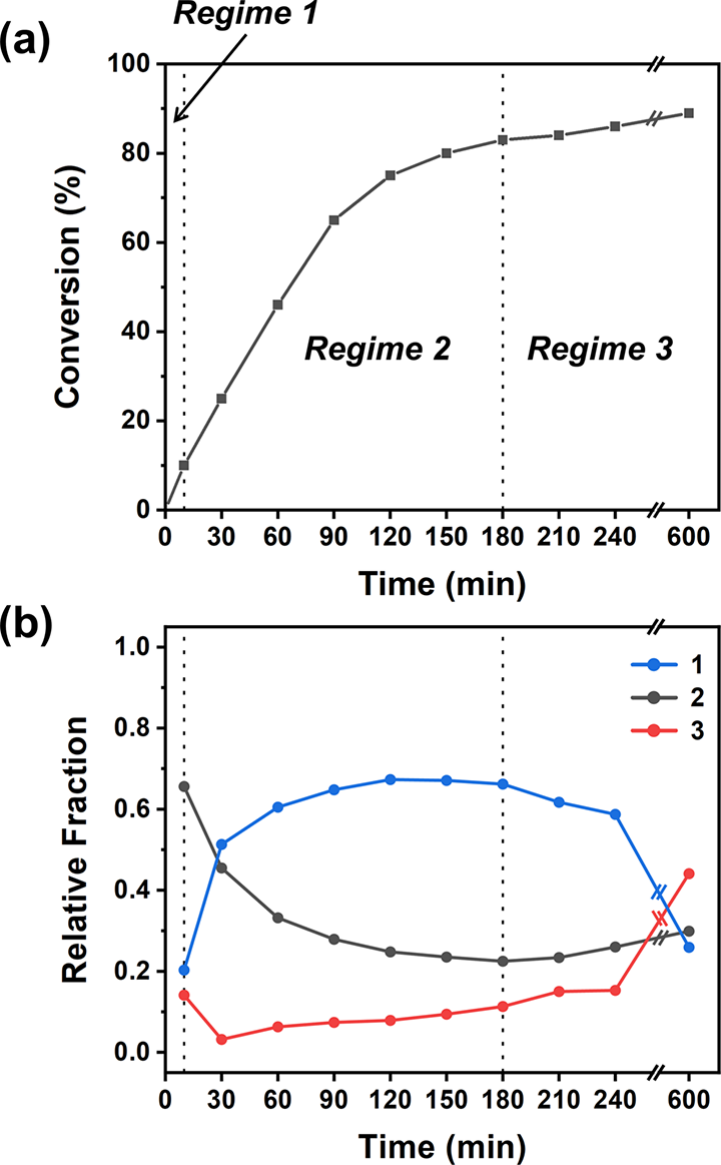
(a) Plot of the depolymerization conversion and (b) the relative fraction of **1**, **2**, and **3** as a function of time.

The second regime is characterized by the great bulk of the depolymerization that occurs (10–83%). As depolymerization commenced, a rapid increase in the fraction of **1** was observed, signaling a rapid conversion of PMMA-DTB to **1** by the unzipping of MMA until the final unit. As the depolymerization conversion increased from 10% to 83%, the fraction of **1** in dithiobenzoate increased from 20% to 67% and clearly became the major end-group-derived depolymerization product.

In the third and final regime, no meaningful depolymerization occurred (83–89%), but a clear decrease in the fraction of **1** was observed (from 67% to 26%), presumably corresponding to the conversion of **1** (and some PMMA-DTB) to **2** and **3**. This is not surprising, as a constant generation of dioxane-derived radicals is expected to displace the unimeric “R-group” of **1**, especially since the precursor radicals to form **2** and **3** are 2° and 1° carbon-centered radicals, respectively, and thus, the unimeric R-group is a better homolytic leaving group. Another hypothesis for the decrease in unimer fraction at the later stages of the depolymerization is the possible degradation of the RAFT agent via a Chugaev-type elimination at prolonged reaction times. All in all, this depolymerization pathway can be summarized as shown in Figure S24, with the two types of solvent radicals acting as one of the triggers for depolymerization and the formation of unimer and solvent-derived dithiobenzoates and finally converting the generated unimer to the solvent-derived dithiobenzoate.

We were subsequently interested in testing the ability of these recovered products to potentially act as RAFT agents, considering that **1**, **2**, and **3** are essentially dithiobenzoates with different R-groups (Figure 4a). In our previous work, we have shown that the mixture of small-molecule products obtained from the depolymerization of poly(oligo(ethylene glycol) methyl ether methacrylate) could be used in a RAFT repolymerization and produce polymers with low dispersity. However, the structure of the RAFT agent that was responsible for the controlled polymerization was not identified. To test the efficiency of each dithiobenzoate derivative to act as a RAFT agent, MMA was polymerized in the presence of **1**, **2**, or **3**, with azobis(isobutyronitrile) as the initiator. When **1** was employed in the polymerization, a linear increase in the *M*_n_ with respect to monomer conversion alongside a low dispersity (1.10) was observed, signaling a well-controlled RAFT polymerization (Figures 4b,e and S25–S27 and Table S4). In fact, a slightly lower dispersity could be reached with **1** than with the widely used 2-cyano-2-propyl dithiobenzoate (CPDB) under the same conditions (Figure S28). UV-SEC showed rapid incorporation of **1** into the polymer chain, with near-complete consumption in the first hour of polymerization, whereas more than 4 h was needed for CPDB, which explains the higher dispersity for CPDB in the early stages of polymerization (Figures S25 and S28). This suggests a much faster fragmentation of the unimer R group compared to that of CPDB. In the presence of **2** as a potential RAFT agent, a polymerization behavior resembling free radical polymerization was observed, with very little correlation between *M*_n_ and monomer conversion (Figures 4c,f, S29, S30, and S33 and Table S5). Similar results were seen in the polymerization of MMA in the presence of **3** (Figures 4d,g, S31, and S32 and Table S6), also indicating an uncontrolled polymerization. From a structural viewpoint, the low chain-transfer activities of **2** and **3** are unsurprising, as the fragmentation of PMMA is expected to be favorable over that of the R-groups of **2** and **3**, leading to poor incorporation of the dithiobenzoate to the PMMA chain. Taken together, the unimer species act as a very powerful RAFT agent, as evidenced by its rapid consumption and the very low final dispersity obtained.

**Figure 4:**
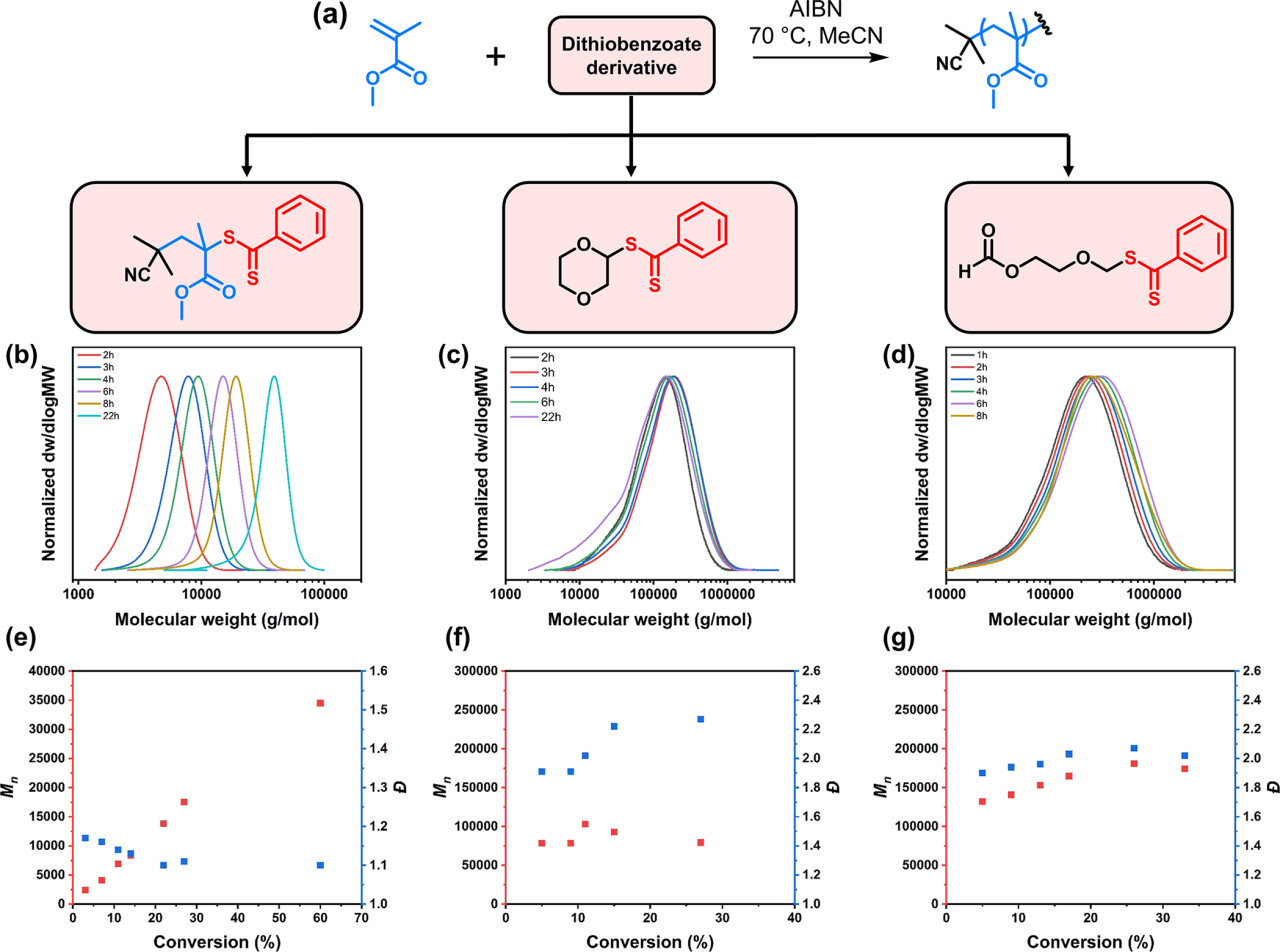
(a) Scheme of the polymerization of MMA in the presence of either **1**, **2**, or **3** at 70 °C in the presence of 10 mol % AIBN relative to the end-group derived products. (b–d) SEC traces and (e, f) plots of *M*_n_ and dispersity versus monomer conversion for the polymerization of MMA in the presence of (b, e) **1**, (c, f) **2**, and (d, g) **3**.

To investigate whether the unimer and solvent-derived species observed are a result of a specific monomer, solvent, and RAFT agent combination, we performed a series of additional experiments where we sequentially changed each of the three components involved (i.e., monomer, RAFT agent, and solvent) and analyzed their respective depolymerization products. A trithiocarbonate-terminated PMMA (PMMA-TTC) was first synthesized by polymerizing MMA using 2-cyano-2-propyl dodecyl trithiocarbonate as the RAFT agent (Figures S34 and S35). PMMA-TTC was then depolymerized in 1,4-dioxane under similar conditions to that of PMMA-DTB and the end-group derived products were purified by flash column chromatography (Figures S3 and S36 and Table S8). Similar to PMMA-DTB, TTC analogues of the unimer (**4**) and solvent-derived end-groups (**5** and **6**) were identified, confirming that these products are not dithiobenzoate-specific (Figures 5a and S37–S47). Changing the monomer to benzyl methacrylate to afford poly(benzyl methacrylate)-DTB (Figure S48 and Table S9) also gave a similar set of products, a unimer with a single benzyl methacrylate unit (**7**) and the same two solvent-derived end-groups, as seen in PMMA-DTB (**2** and **3**) (Figures 5b and S49–S55 and Table S10). Finally, we conducted a depolymerization of PMMA-DTB in *p*-xylene instead of 1,4-dioxane and purified the dithiobenzoate products by flash column chromatography (Figure S56 and Table S11). As expected, the same unimer product (**1**) as that seen in the case of 1,4-dioxane was identified. Additionally, we identified a *p*-xylene-derived dithiobenzoate compound (**8**; Figures 5c and S57–S62), a clearly different product compared to those seen in depolymerization in 1,4-dioxane. This suggests that, under the depolymerization conditions employed, a *p*-xylene radical centered on the methyl carbon can be generated through various pathways such as the reaction with impurities (e.g., trace metals, sulfur-containing compounds, etc.) or chain-transfer from other radical sources.

**Figure 5:**
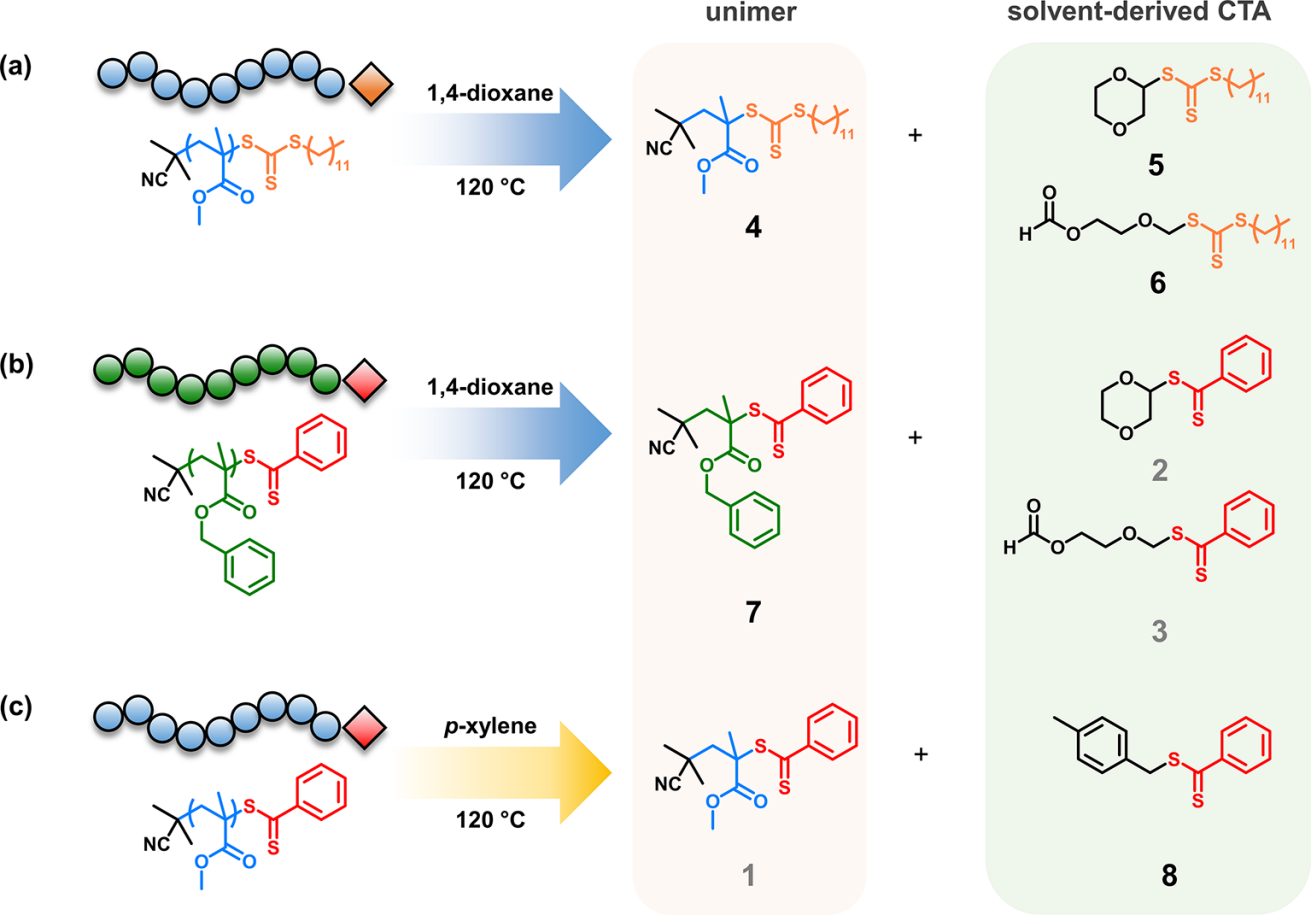
End-group derived products from the depolymerization of (a) PMMA-TTC in 1,4-dioxane, (b) PBzMA-DTB in 1,4-dioxane, and (c) PMMA-DTB in *p*-xylene.

In conclusion, we identified the fate of the RAFT end-group during the thermal depolymerization of polymethacrylates. We could consistently identify a unimer- and solvent-derived end-group as the small-molecule products of depolymerization, regardless of the end-group, monomer, and solvent employed. Detailed investigation of the evolution of these species during depolymerization revealed a high fraction of solvent-derived end-groups in the early stages of the reaction, strongly suggesting a significant solvent-initiated depolymerization, although other pathways cannot be excluded from these results. Finally, the unimer product was found to be a high-activity RAFT agent, superior to the commercially available 2-cyano-2-propyl dithiobenzoate, in the controlled polymerization of MMA. These results advance our mechanistic understanding of thermal RAFT depolymerizations and open up an unexpected and novel approach for the synthesis of RAFT methacrylate unimers.

## Supporting Information Available

General information, experimental procedures, ^1^H NMR spectra, and SEC traces (PDF)
