# Supplementary material for: Fate of the RAFT End-Group in the Thermal Depolymerization of Polymethacrylates
Source: ACS Macro Lett. 2023 Aug 24;12(9):1207–12. doi: 10.1021/acsmacrolett.3c00418 (PMC10515620; doi:10.1021/acsmacrolett.3c00418)
Supplement: Supplementary file 1 — mz3c00418_si_001.pdf [file mz3c00418_si_001.pdf]

Supporting Information

Fate of the RAFT End-Group in the Thermal Depolymerization of Polymethacrylates

Florian Häfliger,[1] Nghia P. Truong,[1,2] Hyun Suk Wang,[1]* Athina Anastasaki[1]*

[1]Laboratory of Polymeric Materials, Department of Materials, ETH Zurich, Vladimir-Prelog-Weg 5, Zurich, Switzerland

[2] Monash Institute of Pharmaceutical Sciences, Monash University, 399 Royal Parade, Parkville, VIC 3152, Australia.

Table of Contents

1. Methods 1
1.1 Materials 1
1.2 Nuclear Magnetic Resonance Spectroscopy (NMR) 1
1.3 Size-Exclusion Chromatography (SEC) 1
1.4 Mass Spectrometry (MS) 1
1.5 Polymerization of MMA with DTB 1
1.6 Polymerization of MMA with TTC 1
1.7 Polymerization of BzMA with DTB 2
1.8 Purification of PMMA, PBzMA 2
1.9 Depolymerization Procedure for PMMA and PBzMA 2
1.10 Polymerization Procedure for MMA using 1 as CTA 2
1.11 Polymerization Procedure for MMA using 2 as CTA 3
1.12 Polymerization Procedure for MMA using 3 as CTA 3
1.13 Determination of depolymerization conversion 3
1.14 Determination of small molecule fraction ratio 4
2. Structure Elucidation 5
2.1 NMR and MS data 5
3. Polymerization & Depolymerization of PMMA-DTB 7
3.1 Synthesis of PMMA-DTB 7
3.2 Depolymerization of PMMA-DTB 9
3.3 PMMA-DTB 5 mM kinetics interval 31
4. Polymerization using depolymerization products 33
4.1 Polymerization of MMA using 1 as CTA 33
4.2 Polymerization of MMA using CPDB as CTA 36
4.3 Polymerization of MMA in the presence of 2 37
4.4 Polymerization of MMA in the presence of 3 39
4.5 Free Radical Polymerization of MMA 41
5. Polymerization & Depolymerization of PMMA-TTC 42
5.1 Synthesis of PMMA-TTC 42
5.2 Depolymerization of PMMA-TTC 44
6. Polymerization & Depolymerization of PBzMA-DTB 56
6.1 Synthesis of PBzMA-DTB 56
6.2 Depolymerization of PBzMA-DTB 57
7. Depolymerization of PMMA-DTB in *p*-xylene 64

Table of Figures

Figure S1. SEC trace of PMMA synthesized via RAFT polymerization with DTB as the chain transfer agent ([MMA]:[CTA]:[AIBN] = 50:1:0.1). 7
Figure S2. ^{1}H-NMR spectrum of purified PMMA-DTB prior to depolymerization........8
Figure S3. Photo of the flash column chromatography performed to purify the depolymerization reaction products from PMMA-DTB (left), PMMA-TTC (middle) and PBzMA (right). 10
Figure S4. ^{13}C NMR of molecule **1** with indicated carbon atoms.............................11
Figure S5. ^{1}H NMR of molecule **1** with indicated hydrogen atoms. 12
Figure S6. 2D NMR (COSY) of molecule **1** with indicated H-H correlations............13
Figure S7. 2D NMR (HSQC) of molecule **1** with indicated H-C correlations. 14
Figure S8. 2D NMR (HMBC) of molecule **1** with indicated H-C correlations. 15
Figure S9. Full MS spectrum of molecule **1** (top), experimental isotope pattern (middle) and predicted isotope pattern (bottom)..16
Figure S10. ^{13}C NMR of molecule **2** with indicated carbon atoms...........................17
Figure S11. ^{1}H NMR of molecule **2** with indicated hydrogen atoms. 18
Figure S12. 2D NMR (COSY) of molecule **2** with indicated H-H correlations...........19
Figure S13. 2D NMR (HSQC) of molecule **2** with indicated H-C correlations. 20
Figure S14. 2D NMR (HMBC) of molecule **2** with indicated H-C correlations. 21
Figure S15. Full MS spectrum of molecule **2** (top), experimental isotope pattern (middle) and predicted isotope pattern (bottom)..22
Figure S16. ^{13}C NMR of molecule **3** with indicated carbon atoms...........................23
Figure S17. ^{1}H NMR of molecule **3** with indicated hydrogen atoms. 24
Figure S18. 2D NMR (HSQC) of molecule **3** with indicated H-C correlations. 25
Figure S19. 2D NMR (HMBC) of molecule **3** with indicated H-C correlations. 26
Figure S20. Full MS spectrum of molecule **3** (top), experimental isotope pattern (middle) and predicted isotope pattern (bottom)..27
Figure S21. UV-SEC traces of molecules **1**, **2**, and **3**..28
Figure S22. Formation of solvent-based radicals. (a) Proposed mechanism leading to 1,4-dioxanyl radical. (b) Proposed mechanism leading to 2-methoxyethyl formate radical. 29
Figure S23. Characteristic ^{1}H NMR used for the calculation of the relative fraction of the end-group derived molecules.. 30
Figure S24. Proposed mechanism of solvent-initiated thermal RAFT depolymerization in 1,4-dioxane. (a) Initiation of the depolymerization achieved through 1,4-dioxane-based radicals with pathway *i* leading to **2** and pathway *ii* leading to **3**. (b) Depropagation generating monomer and unimer **1**. (c) Degradation of unimer **1** to form **2** and **3**. 32
Figure S25. SEC trace (UV detector) of repolymerization using **1** as a CTA. Fast CTA consumption can be observed as well as incorporation of the chromophore in the resulting PMMA polymer. 33
Figure S26. Plot of monomer conversion and ln(1/(1-x)) versus time during the polymerization of MMA with **1** as the CTA..34
Figure S27. UV and RI SEC traces after 3 h of MMA polymerization in the presence of **1** as the CTA...35
Figure S28. (a) SEC trace (UV detector) of repolymerization using CPDB as a CTA. (b) Plot of M_n and dispersity as a function of conversion. ([CTA]:[MMA]:[AIBN] = 1:500:0.1) 36
Figure S29. SEC trace (UV detector) of MMA polymerization in the presence of **2.** 37

Figure S30. Plot of monomer conversion and ln(1/(1-x)) versus time during the polymerization of MMA in the presence of **2**. 38
Figure S31. SEC trace (UV detector) of MMA polymerization in the presence of **3.** 39
Figure S32. Plot of monomer conversion and ln(1/(1-x)) versus time during the polymerization of MMA in the presence of **3**. 40
Figure S33. (a) SEC trace (RI detector) and (b) Plot of M_n and dispersity versus monomer conversion during the free radical polymerization of MMA.......................41
Figure S34. SEC trace of PMMA synthesized via RAFT polymerization with TTC as the chain transfer agent ([MMA]:[CTA]:[AIBN] = 50:1:0.1). 42
Figure S35. ^{1}H-NMR spectrum of purified PMMA-TTC prior to depolymerization....43
Figure S36. SEC trace of PMMA-TTC depolymerization at 120 °C at 25 mM. UV-Vis (left) and RI (right). 44
Figure S37. Full MS spectrum of molecule **4** (top), experimental isotope pattern (middle) and predicted isotope pattern (bottom)...45
Figure S38. ^{13}C NMR of molecule **5** with indicated carbon atoms...........................46
Figure S39. ^{1}H NMR of molecule **5** with indicated hydrogen atoms. 47
Figure S40. 2D NMR (HSQC) of molecule **5** with indicated H-C correlations. 48
Figure S41. 2D NMR (HMBC) of molecule **5** with indicated H-C correlations. 49
Figure S42. Full MS spectrum of molecule **5** (top), experimental isotope pattern (middle) and predicted isotope pattern (bottom)...50
Figure S43. ^{13}C NMR of molecule **6** with indicated carbon atoms...........................51
Figure S44. ^{1}H NMR of molecule **6** with indicated hydrogen atoms. 52
Figure S45. 2D NMR (HSQC) of molecule **6** with indicated H-C correlations. 53
Figure S46. 2D NMR (HMBC) of molecule **6** with indicated H-C correlations. 54
Figure S47. Full MS spectrum of molecule **6** (top), experimental isotope pattern (middle) and predicted isotope pattern (bottom)...55
Figure S48. SEC trace of PBzMA synthesized via RAFT polymerization with DTB as the chain transfer agent ([MMA]:[CTA]:[AIBN] = 52:1:0.1). 56
Figure S49. SEC trace of PBzMA-DTB depolymerization at 120 °C at 25 mM. UV-Vis (left) and RI (right). 57
Figure S50. ^{1}H-NMR spectrum of purified PBzMA-DTB prior to depolymerization. .58
Figure S51. ^{13}C NMR of molecule **7** with indicated carbon atoms...........................59
Figure S52. ^{1}H NMR of molecule **7** with indicated hydrogen atoms. 60
Figure S53. 2D NMR (HSQC) of molecule **7** with indicated H-C correlations. 61
Figure S54. 2D NMR (HMBC) of molecule **7** with indicated H-C correlations. 62
Figure S55. Full MS spectrum of molecule **7** (top), experimental isotope pattern (middle) and predicted isotope pattern (bottom)...63
Figure S56. SEC trace of PMMA-DTB in p-xylene depolymerization at 120 °C at 25 mM. UV-Vis (left) and RI (right). 64
Figure S57. ^{13}C NMR of molecule **8** with indicated carbon atoms...........................65
Figure S58. ^{1}H NMR of molecule **8** with indicated hydrogen atoms. 66
Figure S59. 2D NMR (COSY) of molecule **8** with indicated H-C correlations...........67
Figure S60. 2D NMR (HSQC) of molecule **8** with indicated H-C correlations. 68
Figure S61. 2D NMR (HMBC) of molecule **8** with indicated H-C correlations. 69
Figure S62. Full MS spectrum of molecule **8** (top), experimental isotope pattern (middle) and predicted isotope pattern (bottom)...70

Table of Tables

Table S1. Characterization of PMMA-DTB. 7
Table S2. Characterization of PMMA-DTB depolymerization. 9
Table S3. Kinetic Data on the depolymerization of PMMA-DTB in 1,4-dioxane at 5 mM and 120 °C. The Relative fraction ratios have been normalized to 1. 31
Table S4. Characterization of repolymerization polymer using **1** as CTA. The data shows good agreement of the observed molecular weight with the theoretical M_n. ..33
Table S5. Characterization of repolymerization polymer in the presence of **2**. 38
Table S6. Characterization of repolymerization polymer in the presence of **3**. 40
Table S7. Characterization of PMMA-TTC..42
Table S8. Characterization of PMMA-TTC depolymerization. 44
Table S9. Characterization of PBzMA-DTB. 56
Table S10. Characterization of PBzMA-DTB depolymerization. 57
Table S11. Characterization of PMMA-TTC depolymerization. 64

1. Methods

1.1 Materials

All materials were purchased from either Sigma Aldrich or Fischer Scientific unless otherwise stated. Benzyl methacrylate (BzMA, >98.0%) was purchased from Tokyo Chemical Industries. Monomers were filtered through basic alumina before use.

1.2 Nuclear Magnetic Resonance Spectroscopy (NMR)

^{1}H NMR spectra were recorded on a Bruker Avance-300 and 500 MHz spectrometer using acetone-d_6 or $CDCl_3$ as the NMR solvent. Chemical shifts are given in ppm, downfield from tetramethylsilane (TMS) and referenced to residual solvent proton signals.

1.3 Size-Exclusion Chromatography (SEC)

SEC was measured on a Shimadzu equipment comprising a CBM-20A system controller, LC-20AD pump, SIL-20A automatic injector, 10.0 µm bead-size guard column (50 x 7.5 mm) followed by three KF-805-L columns (300 x 8 mm, bead size: 10 µm, pore size maximum: 5000 Å), SPD-20A ultraviolet detector, and RID-20A differential refractive index detector. The column temperature was maintained at 40 °C using a CTO-20A oven. The flow rate was set to 1 ml/min and with *N,N*-dimethylacetamide (DMAc, Acros, HPLC grade, with 0.03 w/v LiBr) as the eluent. Molecular weights were determined relative to poly(methyl methacrylate) standards wieth molecular weights ranging from 5000 to 1.5 x 10^6 g/mol (Agilent Technologies). All SEC samples were dissolved in DMAc and passed through 0.45 µm filters prior to analysis.

1.4 Mass Spectrometry (MS)

Mass spectrometry (MS) experiments were either performed on a Bruker Maxis I quadrupole-time-of-flight (QTOF) at 4500V emitter voltage with electrospray ionization (ESI) as ion source and direct injection or on a Thermo Scientific QExactive GC Orbitrap (GC-MS) with Ion-Trap (OrbiTrap) and electron impact (EI) as ion source.

1.5 Polymerization of MMA with DTB

Into a 25 ml round bottom flask, 448 mg of 2-cyanoprop-2-yl dithiobenzoate (DTB, 1.2 mmol, 1 equiv) were dissolved in 4 ml acetonitrile (ACN). A stock solution of AIBN (40 mg) was prepared in 2 ml ACN, and 1640 µL of this solution (32.8 mg, 199 µmol, 0.1 equiv) was transferred to the flask. Subsequently, 10.6 mL of MMA (10.0 g, 99.9 mmol, 50 equiv) and a stirrer bar were added, and the flask was sealed with a septum, prior to deoxygenation by nitrogen bubbling for 15 min. Polymerization was conducted in an oil bath at 70 °C for 4h with a 400-rpm stirring rates. Samples were taken every hour under a nitrogen blanket for ^{1}H-NMR analysis and passed through a syringe filter (0.45 µm PTFE membrane) prior to SEC analysis. Polymerization was stopped at 74 % conversion by removing the reaction from the oil bath and removing the septum. Monomer conversions were determined by NMR spectroscopy. The monomer vinyl signals were compared to the combined polymer and monomer ester signals.

1.6 Polymerization of MMA with TTC

Into a 25 ml round bottom flask, 690 mg of 2-cyanoprop-2-yl dodecyl trithiocarbonate (TTC, 1.99 mmol, 1 equiv) were dissolved in 4 ml acetonitrile (ACN). A stock solution of AIBN (40 mg) was prepared in 2 ml ACN, and 1640 µL of this solution (32.8 mg,

199 μmol, 0.1 equiv) was transferred to the flask. Subsequently, 10.6 mL of MMA (10.0 g, 99.9 mmol, 50 equiv) and a stirrer bar were added, and the flask was sealed with a septum, prior to deoxygenation by nitrogen bubbling for 15 min. Polymerization was conducted in an oil bath at 70 °C for 4h with a 400-rpm stirring rates. Samples were taken every hour under a nitrogen blanket for ^{1}H-NMR analysis and passed through a syringe filter (0.45 μm PTFE membrane) prior to SEC analysis. Polymerization was stopped at 54 % conversion by removing the reaction from the oil bath and removing the septum. Monomer conversions were determined by NMR spectroscopy. The monomer vinyl signals were compared to the combined polymer and monomer ester signals.

1.7 Polymerization of BzMA with DTB

Into a 25 ml round bottom flask, 255 mg of 2-cyanoprop-2-yl dithiobenzoate (DTB, 1.14 mmol, 1 equiv) were dissolved in 4 ml acetonitrile (ACN). A stock solution of AIBN (20 mg) was prepared in 1 ml ACN, and 932 μL of this solution (18.64 mg, 114 μmol, 0.1 equiv) was transferred to the flask. Subsequently, 10.6 mL of BzMA (10.6 g, 59.0 mmol, 52 equiv) and a stirrer bar were added, and the flask was sealed with a septum, prior to deoxygenation by nitrogen bubbling for 15 min. Polymerization was conducted in an oil bath at 70 °C for 4h with a 400-rpm stirring rates. Samples were taken every hour under a nitrogen blanket for ^{1}H-NMR analysis and passed through a syringe filter (0.45 μm PTFE membrane) prior to SEC analysis. Polymerization was stopped at 70 % conversion by removing the reaction from the oil bath and removing the septum. Monomer conversions were determined by NMR spectroscopy. The monomer vinyl signals were compared to the combined polymer and monomer methylene signal from benzyl group.

1.8 Purification of PMMA, PBzMA

Polymers were precipitated three times in a 3:2 mixture cold methanol:hexane and vacuum-filtered using a Buchner funnel. The precipitates were dried in a vacuum oven for at least 12h before use.

1.9 Depolymerization Procedure for PMMA and PBzMA

In a 125 ml Schlenk tube, 21.5 mg of PMMA was dissolved in 40 ml 1,4-dioxane (5.17 mM of MMA repeat unit). The Schlenk tube was sealed with a rubber septum and deoxygenated by nitrogen bubbling for 20 min. The Schlenk tube was then put into a 120 °C oil bath to start the reaction. The Schlenk tube was submerged into the oil bath until the surface of the solution inside was at the same height as the oil bath. To take samples, the reaction was periodically removed from the oil bath and quickly added to a cold water bath until the solution cooled to room temperature. The solution was then sampled under a nitrogen blanket. For SEC samples, 800 μL of the sample solution was blow-dried, dissolved in DMAc and passed through a syringe filter (0.45 μm PTFE membrane).

1.10 Polymerization Procedure for MMA using 1 as CTA

Into a 10 ml test tube, 5.95 mg of molecule **1** (18.5 μmol, 1 equiv) was added. A stock solution of AIBN (1.314 mg) was prepared in 4 ml ACN, and 925 μL of this solution (0.304 μg, 1.85 μmol, 0.1 equiv) was transferred to the tube. Subsequently, 0.986 mL of MMA (927 mg, 9.26 mmol, 500 equiv) and a stirrer bar were added, and the tube was sealed with a septum, prior to deoxygenation by nitrogen bubbling for 15 min.

Repolymerization was conducted in an oil bath at 70 °C for 8h with a 400-rpm stirring rates. Samples were taken every hour under a nitrogen blanket for ^{1}H-NMR analysis and passed through a syringe filter (0.45 µm PTFE membrane) prior to SEC analysis. Repolymerization was left until 22h and stopped at 60 % conversion by removing the reaction from the oil bath and removing the septum. Monomer conversions were determined by NMR spectroscopy. The monomer vinyl signals were compared to the combined polymer and monomer ester signals.

1.11 Polymerization Procedure for MMA using 2 as CTA

Into a 10 ml test tube, 2.2 mg of molecule **2** (9 µmol, 1 equiv) was added. A stock solution of AIBN (1.314 mg) was prepared in 4 ml ACN, and 458 µL of this solution (0.150 µg, 0.92 µmol, 0.1 equiv) was transferred to the tube. Subsequently, 0.487 mL of MMA (458 mg, 4.58 mmol, 500 equiv) and a stirrer bar were added, and the tube was sealed with a septum, prior to deoxygenation by nitrogen bubbling for 15 min. Repolymerization was conducted in an oil bath at 70 °C for 8h with a 400-rpm stirring rates. Samples were taken every hour under a nitrogen blanket for ^{1}H-NMR analysis and passed through a syringe filter (0.45 µm PTFE membrane) prior to SEC analysis. Repolymerization was left until 22h and stopped at 27 % conversion by removing the reaction from the oil bath and removing the septum. Increasing viscosity due to the large molecular weight led to unreliable data and thus data points after 6h were disregarded for further analysis. Monomer conversions were determined by NMR spectroscopy. The monomer vinyl signals were compared to the combined polymer and monomer ester signals.

1.12 Polymerization Procedure for MMA using 3 as CTA

Into a 10 ml test tube, 4.9 mg of molecule **3** (19 µmol, 1 equiv) was added. A stock solution of AIBN (1.314 mg) was prepared in 4 ml ACN, and 955 µL of this solution (0.314 µg, 1.91 µmol, 0.1 equiv) was transferred to the tube. Subsequently, 1.018 mL of MMA (957 mg, 9.56 mmol, 500 equiv) and a stirrer bar were added, and the tube was sealed with a septum, prior to deoxygenation by nitrogen bubbling for 15 min. Repolymerization was conducted in an oil bath at 70 °C for 8h with a 400-rpm stirring rates. Samples were taken every hour under a nitrogen blanket for ^{1}H-NMR analysis and passed through a syringe filter (0.45 µm PTFE membrane) prior to SEC analysis. Repolymerization was left until 22h and stopped at 57 % conversion by removing the reaction from the oil bath and removing the septum. Increasing viscosity due to the large molecular weight led to unreliable data and thus data points after 8h were disregarded for further analysis. Monomer conversions were determined by NMR spectroscopy. The monomer vinyl signals were compared to the combined polymer and monomer ester signals.

1.13 Determination of depolymerization conversion

Depolymerization conversions were determined in-situ by comparing the monomer vinyl signals to the polymer backbone -CH3 signals (simply taking a sample in dioxane and re-dissolving it in the deuterated solvent, either d_6-acetone or $CDCl_3$). To ensure accurate conversion calculation a second sample was prepared for SEC measurement. The polymer signal (RI detector) at sampling time (t=x) was compared to the signal at start (t=0h) and thus the depolymerization conversion was calculated. Specifically, an exact same sampling volume was required for the SEC measurement (see 1.9). Depolymerization conversions from the two methods deviated by < 10%.

1.14 Determination of small molecule fraction ratio

Small molecule fraction ratio was determined by ^{1}H-NMR spectroscopy. Samples were taken under a nitrogen blanket and then blow-dried. The sample was re-dissolving it in the deuterated solvent (either d_6-acetone or $CDCl_3$). The ratio was determined by comparing the unobstructed, characteristic NMR peaks from the small molecules. For molecule **1** this corresponds to signal **10a** (2.56 ppm) and/or 10b (2.44 ppm) with 1H each. For molecule **2** this corresponds to signal **6** (6.12 ppm) with 1H. For molecule **3** this corresponds to signal **7** (5.61 ppm) with 2H.

2. Structure Elucidation

2.1 NMR and MS data

(1) Methyl 4-cyano-2,4-dimethyl-2-((phenylcarbonothioyl)thio)pentanoate
Red solid, $C_{16}H_{19}NO_2S_2$, 321.45 g/mol.
^{13}C NMR (126 MHz, d_6-Acetone): δ (ppm) 227.39 (**1**, C_{dithio}), 171.96 (**2**, $C_{carbonyl}$), 145.95 (**3**, C_q, ipso), 133.80 (**4**, 1x CH_{arom}, para), 129.59 (**5**, 2x CH_{arom}, meta), 127.51 (**6**, 2x CH_{arom}, ortho), 125.24 (**7**, $C_{nitrile}$), 59.55 (**8**, C_q), 53.43 (**9**, O-CH_3), 46.65 (**10**, CH_2), 31.77 (**11**, C_q), 28.21 (**12**, 2x CH_3), 23.29 (**13**, CH_3).
^{1}H NMR (500 MHz, d_6-Acetone): δ (ppm) 7.93 (**6**, m, $2H_{ortho}$), 7.63 (**4**, $1H_{para}$), 7.47 (**5**, $2H_{meta}$), 3.70 (**9**, s, 3H), 2.56 (**10a**, d, *J* = 15.1 Hz, 1H), 2.44 (**10b**, d, *J* = 15.1 Hz, 1H), 2.02 (**13**, s, 3H), 1.49 (**12**, d, *J* = 3.8 Hz, 6H).
MS (m/z): 322.09 (M+H), 121.06 (Ph-C≡S).

(2) 1,4-Dioxan-2y-yl benzodithioate
Red Solid, $C_{11}H_{12}O_2S_2$, 240.34 g/mol.
^{13}C NMR (126 MHz, d_6-Acetone): δ (ppm) 228.21 (**1**, C_{dithio}), 146.34 (**2**, C_q, ipso), 133.92 (**3**, CH_{arom}, para), 129.57 (**4**, 2x CH_{arom}, meta), 127.76 (**5**, 2x CH_{arom}, ortho), 85.90 (**6**, CH), 70.62 (**7**, CH_2), 67.40 (**8**, CH_2), 65.05 (**9**, CH_2).
^{1}H NMR (500 MHz, d_6-Acetone): δ (ppm) 8.04 (**5**, m, $2H_{ortho}$), 7.66 (**3**, m, $1H_{para}$), 7.51 (**4**, m, $2H_{meta}$), 6.12 (**6**, t, *J* = 2.8 Hz, 1H), 4.14 (**7a**, dd, *J* = 12.3, 2.8 Hz, 1H), 4.04 (**9a**, dt, *J* = 11.9, 3.4 Hz, 1H), 3.97 (**7b**, dd, *J* = 12.3, 2.8 Hz, 1H), 3.81 (**8**, m, 2H), 3.76 (**9b**, dt, *J* = 11.9, 3.4 Hz, 1H).
MS (m/z): 240.03 (M+), 121.01 (Ph-C≡S), 87.04 (Dioxane-H), 77.04 (Ph-H).

(3) (2-(Formyloxy)ethoxy)methyl benzodithioate
Red Solid, $C_{11}H_{12}O_3S_2$, 256.33 g/mol.
^{13}C NMR (126 MHz, $CDCl_3$): δ (ppm) 227.50 (**1**, C_{dithio}), 160.90 (**2**, $C_{carbonyl}$), 145.16 (**3**, C_q, ipso), 133.05 (**4**, CH_{arom}, para), 128.58 (**5**, 2x CH_{arom}, meta), 127.19 (**6**, 2x CH_{arom}, ortho), 76.28 (**7**, CH_2), 68.05 (**8**, CH_2), 62.63 (**9**, CH_2).
^{1}H NMR (500 MHz, $CDCl_3$): δ (ppm) 8.08 (**2**, s, 1H), 8.2 (**6**, m, $2H_{ortho}$), 7.56 (**4**, m, $1H_{para}$), 7.41 (**5**, m, $2H_{meta}$), 5.61 (**7**, s, 2H), 4.34 (**9**, t, *J* = 4.9 Hz, 2H), 3.86 (**8**, t, *J* = 4.9, 2H).
MS (m/z): 279.01 (M+Na), 121.01 (Ph-C≡S), 105.03 (H_2S-CH_2-OC=C=O), 73.02 (HO=CH-O-CH=CH_2).

(4) Methyl 4-cyano-2-(((dodecylthio)carbonothioyl)thio)-2,4-dimethlypentanoate
Yellow Solid, $C_{22}H_{39}NO_2S_3$, 445.74 g/mol.
^{13}C NMR (126 MHz, d_6-Acetone): analysis to be done.
^{1}H NMR (500 MHz, d_6-Acetone): analysis to be done.
MS (m/z): 386.48 ($C_{20}H_{34}S_3$), 201.17 ($C_{12}H_{25}S$), 168.19 ($C_9H_{14}NO_2$), 139.19 ($C_{10}H_{19}$), 125.13 (C_9H_{17}), 111.12 (C_8H_{15}), 97.10 (C_7H_{13}), 41.04 (C_3H_5).

(5) 1,4-Dioxan-2-yl dodecyl carbonotrithioate
Yellow Solid, $C_{17}H_{32}O_2S_3$, 364.62 g/mol.
^{13}C NMR (126 MHz, d_6-Acetone): δ (ppm) 223.69 (**1**, C_{dithio}), 85.62 (**2**, CH), 70.66 (**3**, CH_2), 67.43 (**4**, CH_2), 64.16 (**5**, CH_2), 37.62 (**6**, CH_2), 32.76 (**7**, 8x CH_2), 30.08 (**8**, 8x CH_2), 29.25 (**9**, CH2), 28.78 (**10**, CH_2), 23.45 (**11**, 8x CH_2), 14.48 (**12**, CH_3).

^{1}**H NMR** (500 MHz, d_6-Acetone): δ (ppm) 6.27 (**2**, t, *J* = 2.7 Hz, 1H) 4.06 (**3a**, dd, *J* = 12.3, 2.7 Hz, 1H), 3.99 (**5a**, dt, *J* = 11.7, 3.4 Hz, 1H), 3.88 (**3b**, dd, *J* = 12.3, 2.7 Hz, 1H), 3.74 (**4**, m, 2H), 3.71 – 3.65 (**5b**, dt, *J* = 11.7, 3.4 Hz, 1H), 3.42 (**6**, t, *J* = 7.3 Hz, 2H), 1.72 (**10**, q, *J* = 7.4 Hz, 2H), 1.43 (**9**, m, 2H), 1.30 (**7**,**8**,**11**, m, 16H), 0.88 (**12**, t, *J* = 7.2 Hz, 3H).
MS (m/z): 281.05 ($C_{11}H_{21}O_2S_3$), 202.18 ($C_{12}H_{26}S$), 168.19 ($C_{12}H_{24}$), 140.16 ($C_{10}H_{20}$).

(6) 2-((((dodecylthio)carbonothioyl)thio)methocy)ethyl formate
Yellow Solid, $C_{17}H_{32}O_3S_3$, 380.62 g/mol.
^{13}**C NMR** (126 MHz, d_6-Acetone): δ (ppm) 224.11 (**1**, C_{dithio}), 161.96 (**2**, $C_{carbonyl}$), 76.64 (**3**, CH_2), 68.63 (**4**, CH_2), 63.08 (**5**, CH_2), 37.67 (**6**, CH_2), 32.74 (**7**, 8x CH_2), 28.76 (**8**, CH_2), 27.90 (**9**, CH2), 23.45 (**10**, 8x CH_2), 14.47 (**11**, CH_3).
^{1}**H NMR** (500 MHz, d_6-Acetone): δ (ppm) 8.13 (**2**, s, 1H), 5.64 (**3**, s, 2H), 4.28 (**5**, t, *J* = 4.6 Hz, 2H), 3.81 (**4**, t, *J* = 4.6 Hz, 2H), 3.43 (**6**, t, *J* = 7.5 Hz, 2H), 1.72 (**9**, q, *J* = 6.5 Hz, 3H), 1.42 (**8**, m, 2H), 1.29 (**7**,**10**, m, 16H), 0.88 (**11**, t, *J* = 6.6 Hz, m, 3H).
MS (m/z): 403.14 (M+Na).

(7) benzyl 4-cyano-2,4-dimethyl-2-((phenylcarbonothioyl)thio)pentanoate
Red Solid, $C_{22}H_{23}NO_2S_2$, 397.55 g/mol.
^{13}**C NMR** (126 MHz, d_6-Acetone): δ (ppm) 225.50* (**1**, C_{dithio}), 171.24 (**2**, $C_{carbonyl}$), 146.11 (**3**, C_q, ipso), 136.56 (**4**, C_q, ipso), 133.66 (**5**, $CH_{arom,}$ para), 129.55 (**6**, CH_{arom}, para), 129.40 (**7** 2x CH_{arom}, meta)129.37 (**8**, 2x CH_{arom}, meta), 129.17 (**9** 2x CH_{arom}, ortho), 127.43 (**10**, 2x CH_{arom}, ortho), 125.31 (**11**, C_{nitril}), 68.70 (**12**, CH_2), 59.57 (**13**, C_q), 47.02 (**14**, C_q), 46.49 (**15**, CH_2), 28.26 (**16**, CH_3), 23.31 (**17**, 1x CH_3).
^{1}**H NMR** (500 MHz, d_6-Acetone): δ (ppm) 7.88 (**10**, m, H_{ortho}, 2H), 7.62 (**5**, m, H_{para}, 1H), 7.54 (**6**, m, H_{para}, 1H), 7.46 (**7**, m, H_{meta}, 2H), 7.39 (**8**, m, H_{meta}, 2H), 7.34 (**9**, m, H_{ortho}, 2H), 5.14 (**12**, d, *J* = 2.4 Hz, 2H), 2.61 (**15a**, d, *J* = 15.1 Hz, 1H), 2.45 (**15b**, d, *J* = 15.1 Hz, 1H), 2.04 (**16**, s, 2x 3H), 1.48 (**17**, d, *J* =12.9 Hz, 6H).
MS (m/z): 420.11 (M+Na), 398.13 (M+H), 262.14, 251.24, 91.05.

(8) 4-methtylbenzyl benzodithioate
Orange Solid, $C_{15}H_{14}S$, 258.40 g/mol.
^{13}**C NMR** (126 MHz, d_6-Acetone): δ (ppm) 229.20 (**1**, C_{dithio}), 145.76 (**2**, C_q, ipso), 138.35 (**3**, C_q, ipso), 133.65 (**4**, CH_{arom}, para), 133.04 (**5**, CH_{arom}, para), 130.30 (**6**, 2x CH_{arom}, meta), 130.24 (**7**, 2x CH_{arom}, ortho), 129.53 (**8**, 2x CH_2, meta), 127.65 (**9**, 2x CH_{arom}, ortho), 42.54 (**10**, CH_2), 21.24 (**11**, CH_3).
^{1}**H NMR** (500 MHz, d_6-Acetone): δ (ppm) 8.01 (**9**, m, 2H), 7.61 (**4**, m, 1H), 7.46 (**8**, m, 2H), 7.35 (**7**, m, 2H), 7.17 (**6**, m, 2H), 4.61 (**10**, s, 2H), 2.31 (**11**, s, 3H).
MS (m/z): 258.05 (M+), 121.01 (Ph-C≡S), 105.07 (*p*-Xylylene+H).

3. Polymerization & Depolymerization of PMMA-DTB
3.1 Synthesis of PMMA-DTB

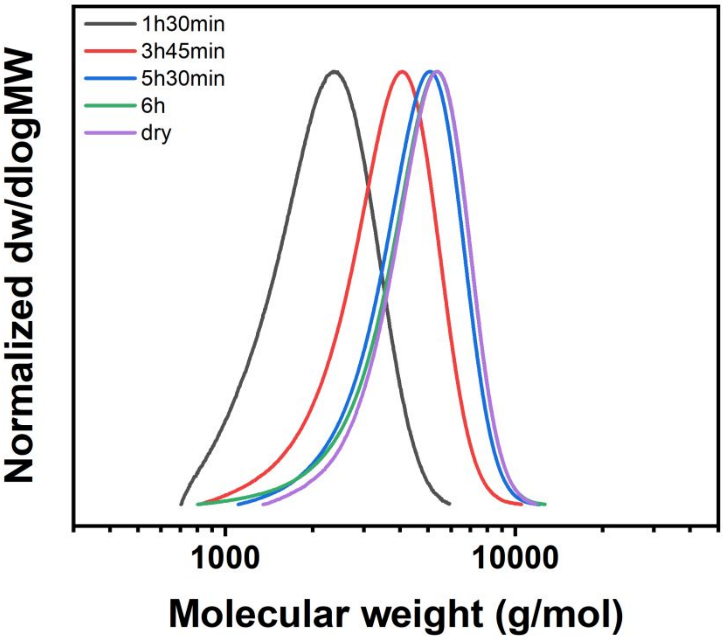

Figure S1. SEC trace of PMMA synthesized via RAFT polymerization with DTB as the chain transfer agent ([MMA]:[CTA]:[AIBN] = 50:1:0.1).

Table S1. Characterization of PMMA-DTB.

Entry	Monomer	[M]:[DTB]:[AIBN]	Time (h)	Conversion (%)	M_n^{theo} (g/mol)	M_n^{SEC} (g/mol)	*Đ*
1	MMA	50:1:0.1	6h	74	3,900	4,600	1.11

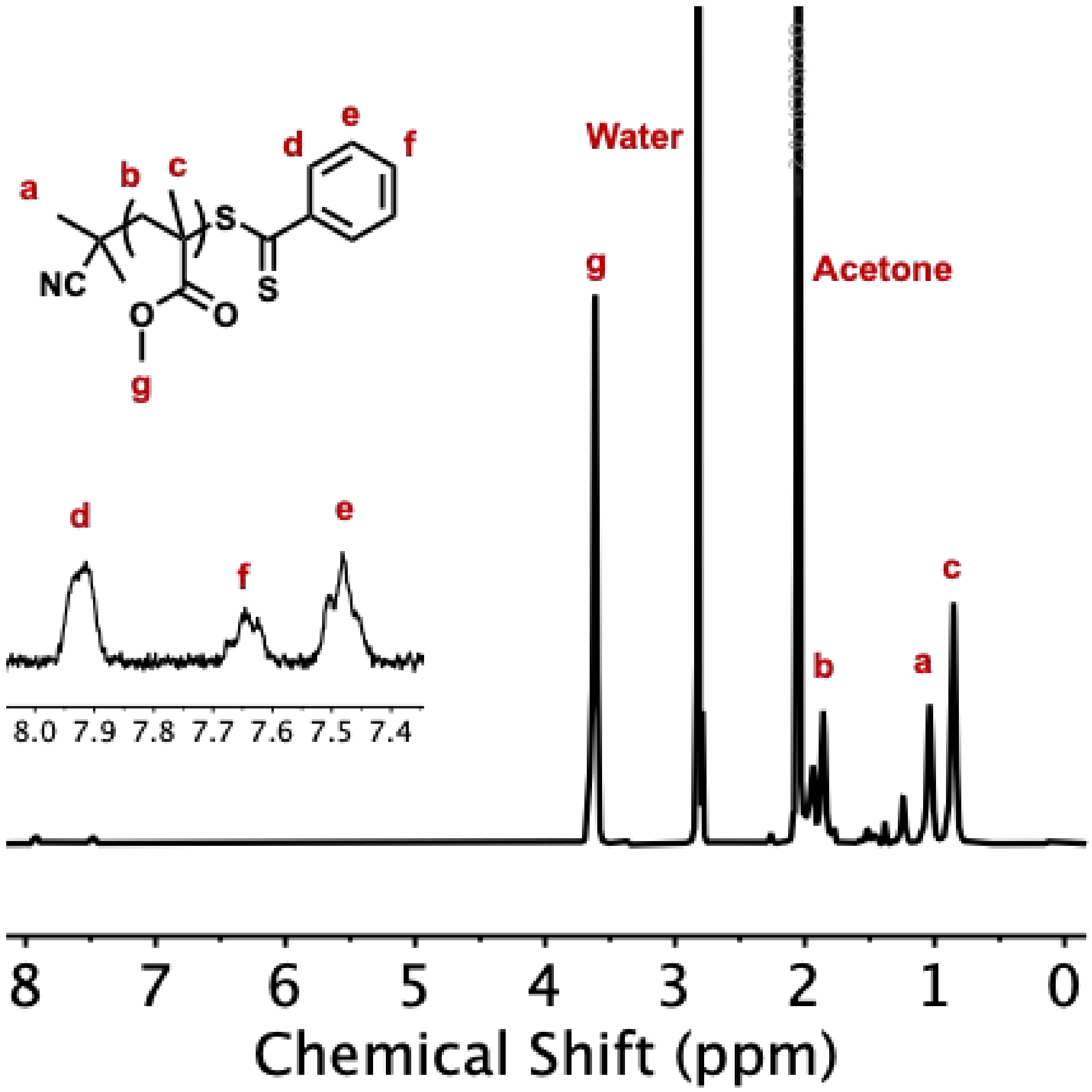

Figure S2. ^{1}H-NMR spectrum of purified PMMA-DTB prior to depolymerization.

3.2 Depolymerization of PMMA-DTB

Table S2. Characterization of PMMA-DTB depolymerization.

Entry	Conc. (mM)	Temp. (°C)	Time (h)	Conversion (%)
1	25	120	6	64

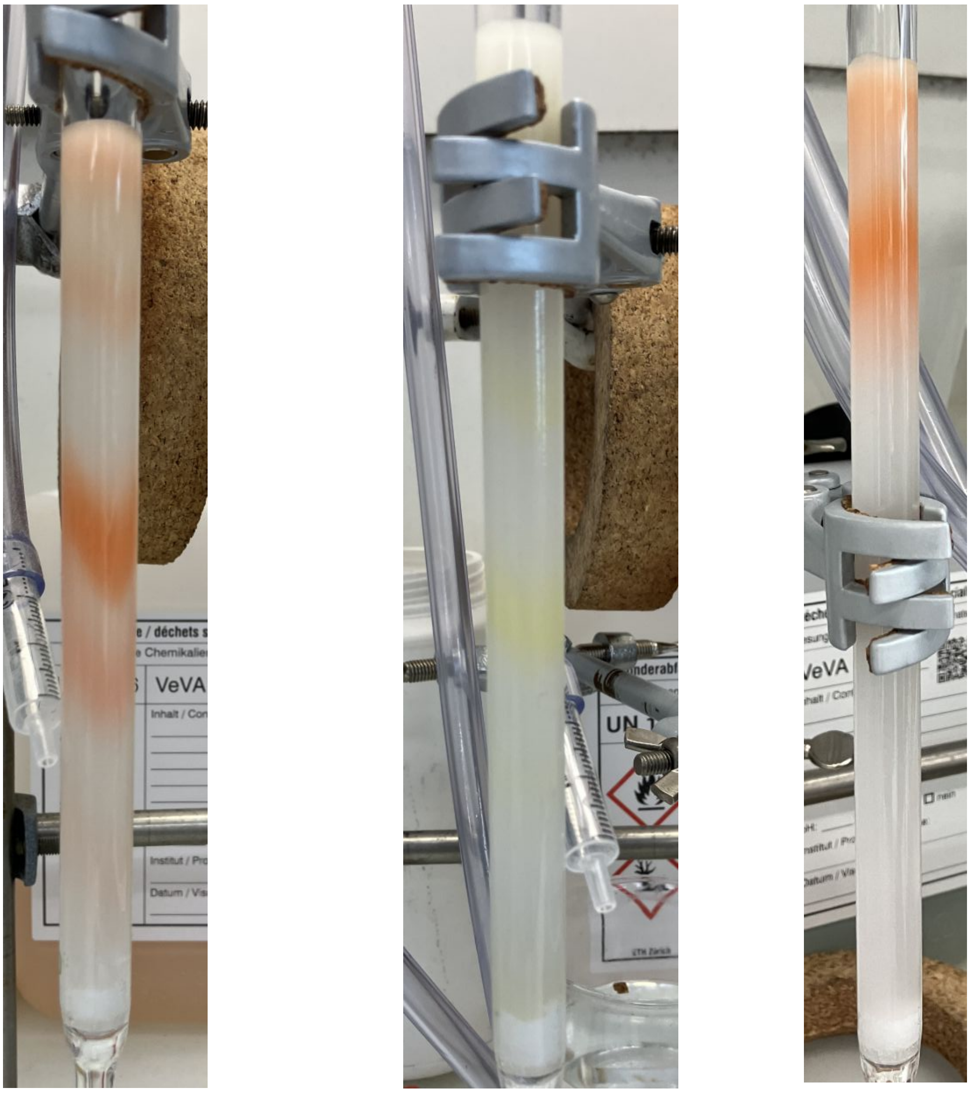

Figure S3. Photo of the flash column chromatography performed to purify the depolymerization reaction products from PMMA-DTB (left), PMMA-TTC (middle) and PBzMA (right).

1 Methyl 4-cyano-2,4-dimethyl-2-((phenylcarbonothioyl)thio)pentanoate

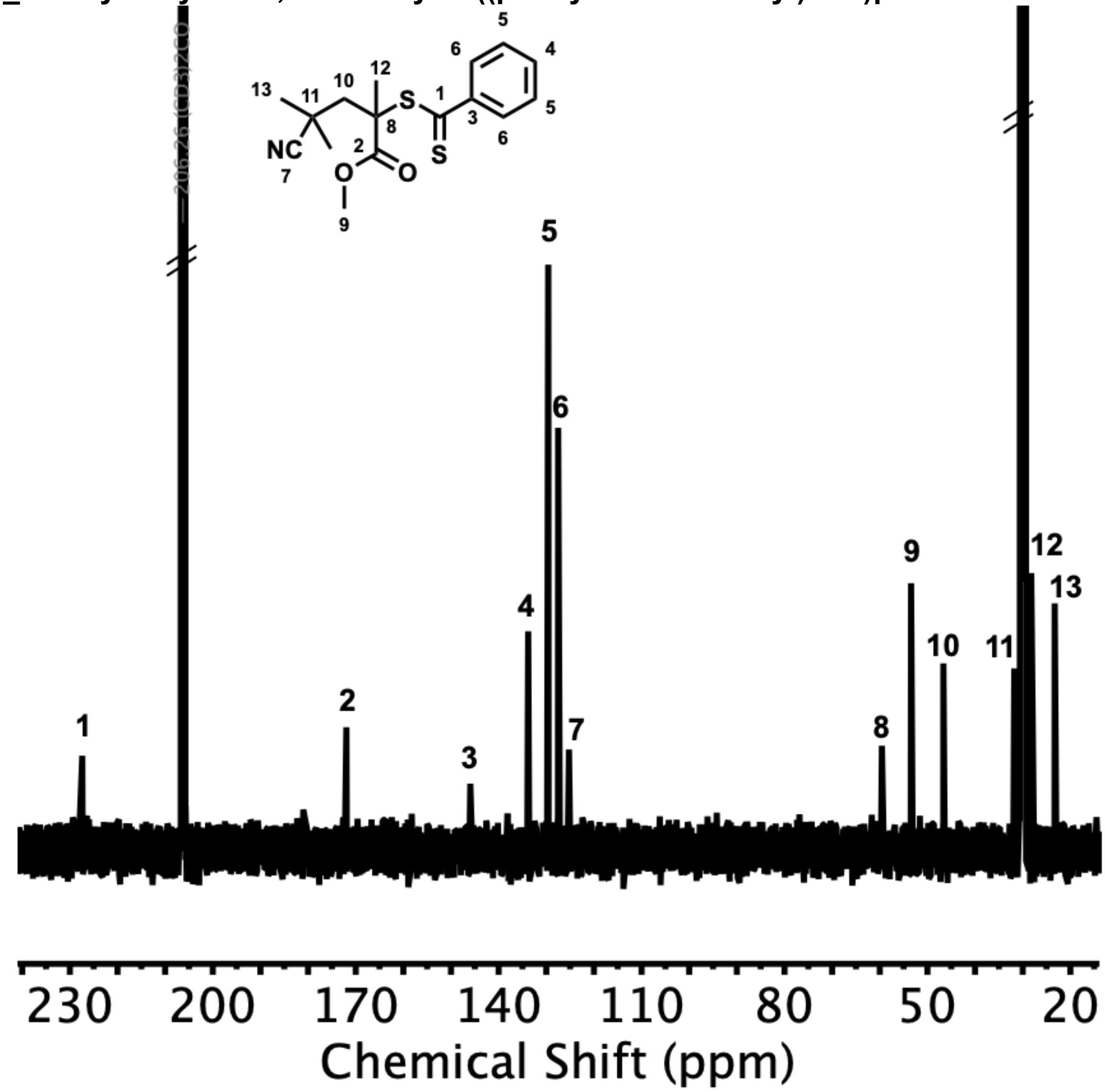

Figure S4. ^{13}C NMR of molecule **1** with indicated carbon atoms.

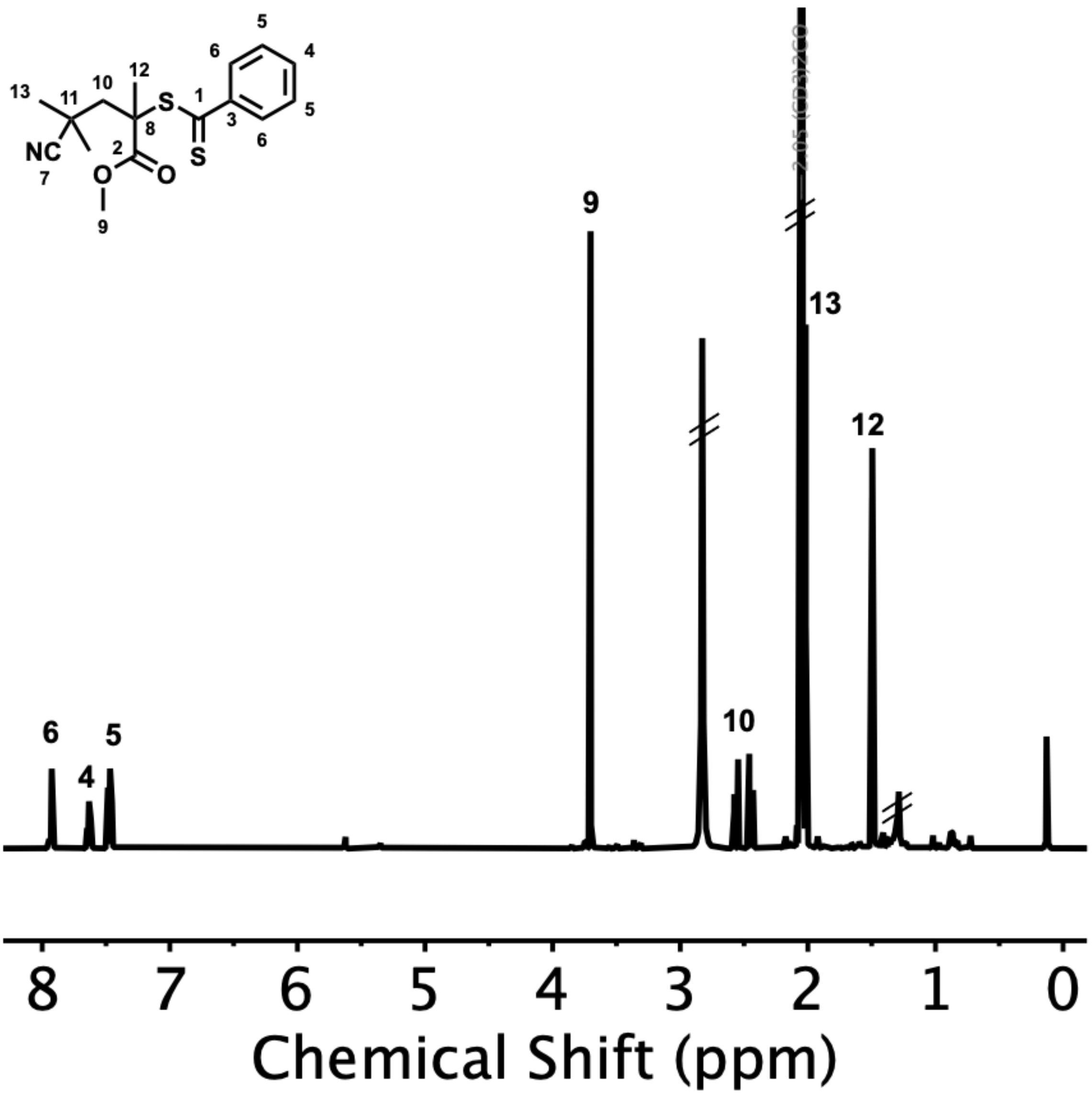

Figure S5. ^{1}H NMR of molecule **1** with indicated hydrogen atoms.

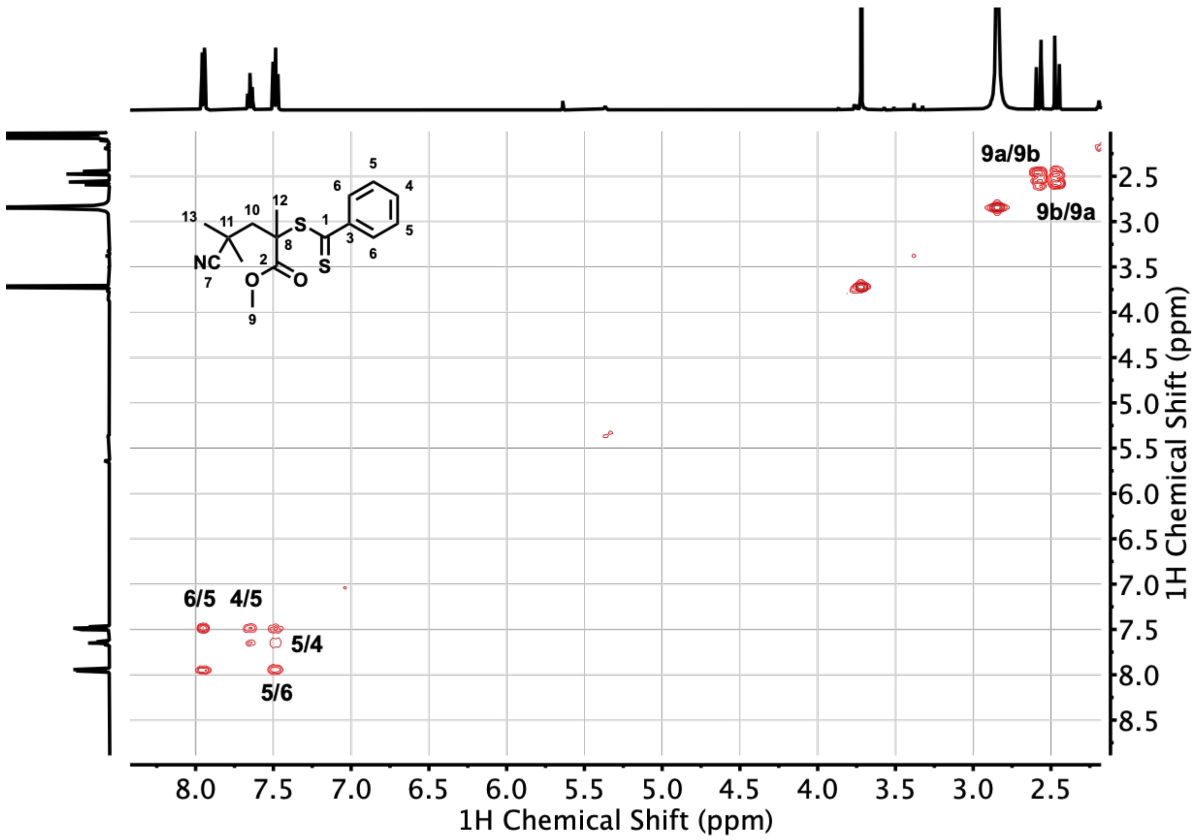

Figure S6. 2D NMR (COSY) of molecule **1** with indicated H-H correlations.

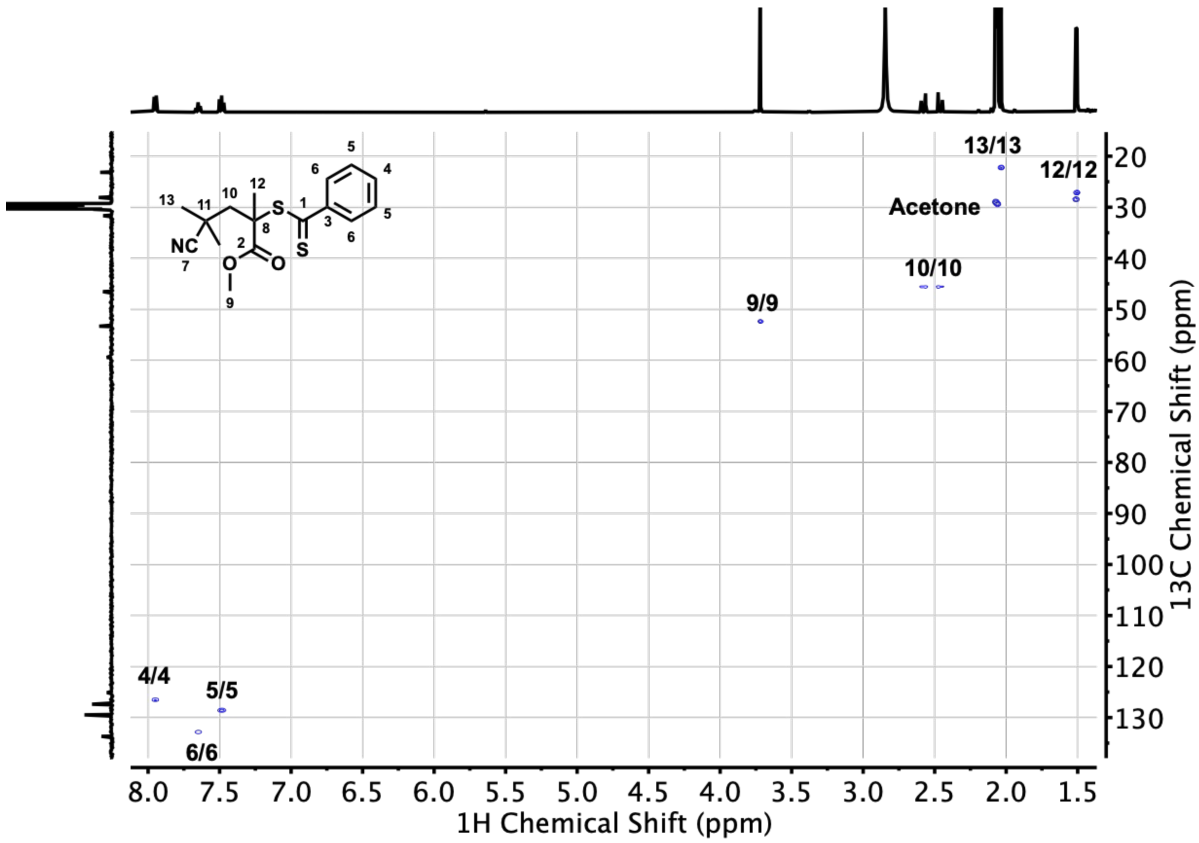

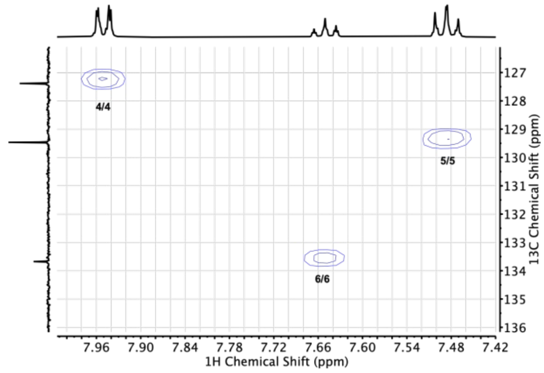

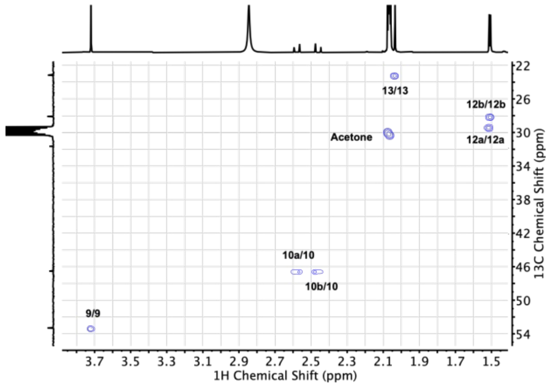

Figure S7. 2D NMR (HSQC) of molecule **1** with indicated H-C correlations.

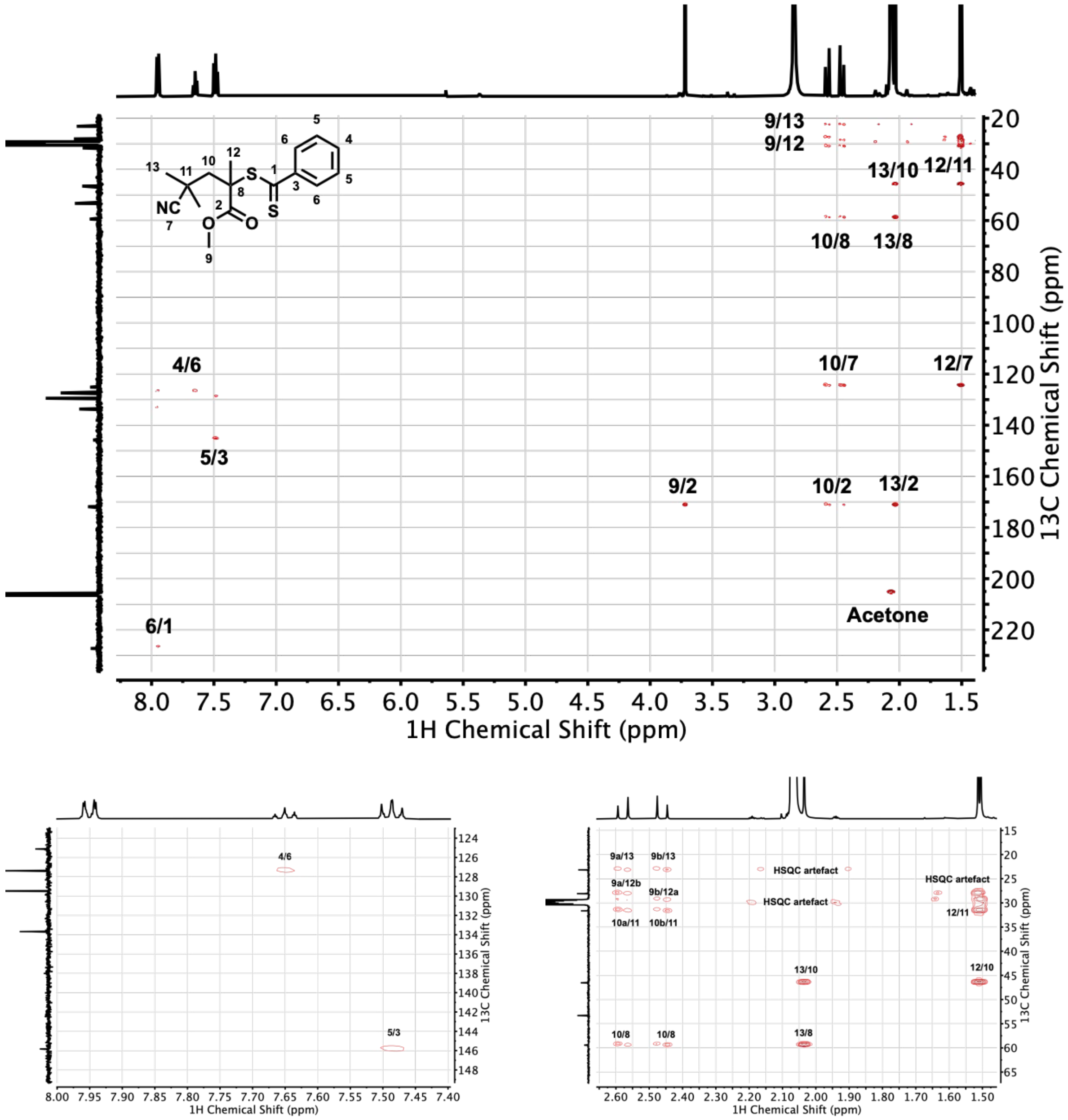

Figure S8. 2D NMR (HMBC) of molecule **1** with indicated H-C correlations.

<u>1</u> Methyl 4-cyano-2,4-dimethyl-2-((phenylcarbonothioyl)thio)pentanoate

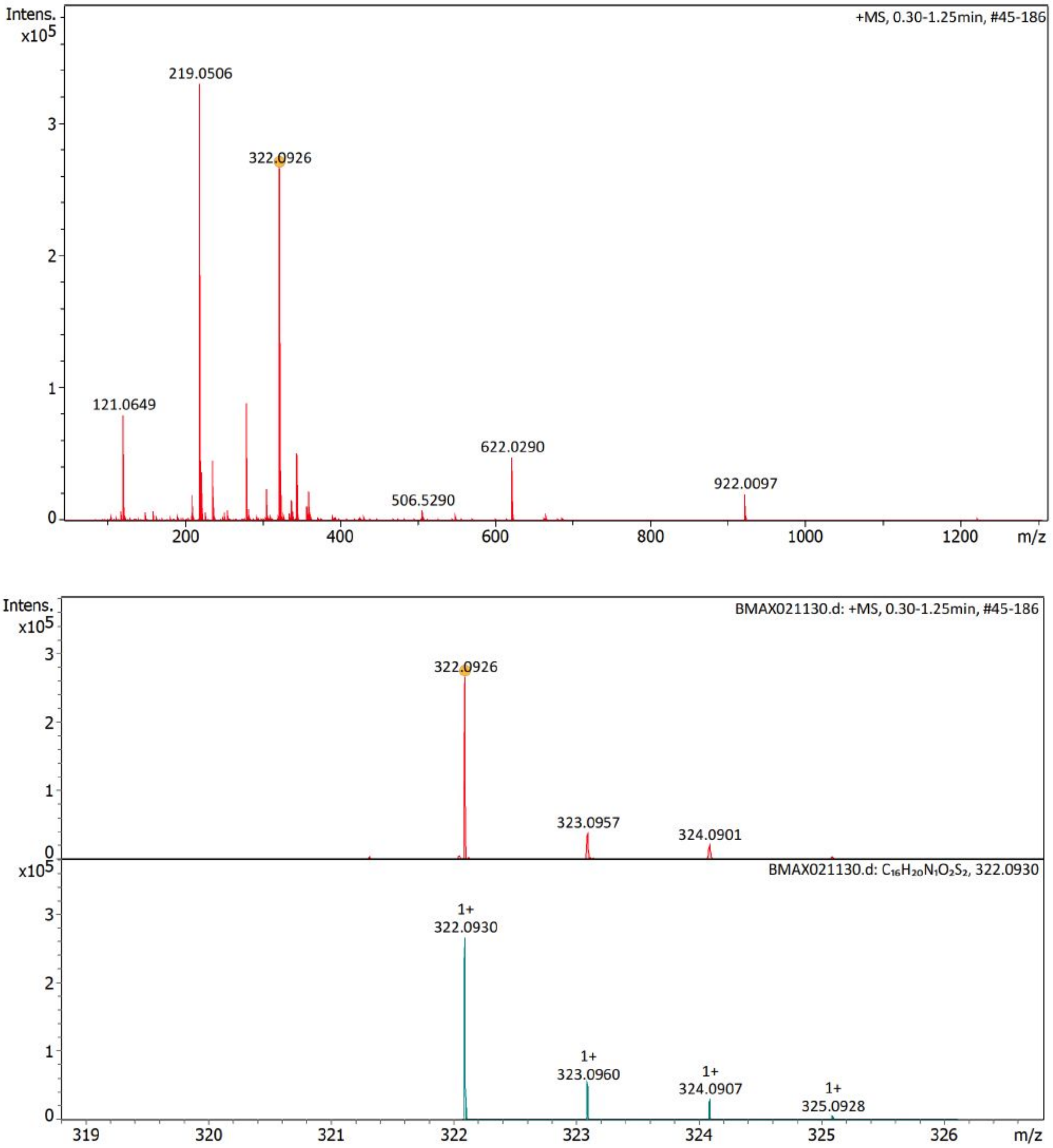

Figure S9. Full MS spectrum of molecule **1** (top), experimental isotope pattern (middle) and predicted isotope pattern (bottom).

2 1,4-Dioxan-2-yl benzodithioate

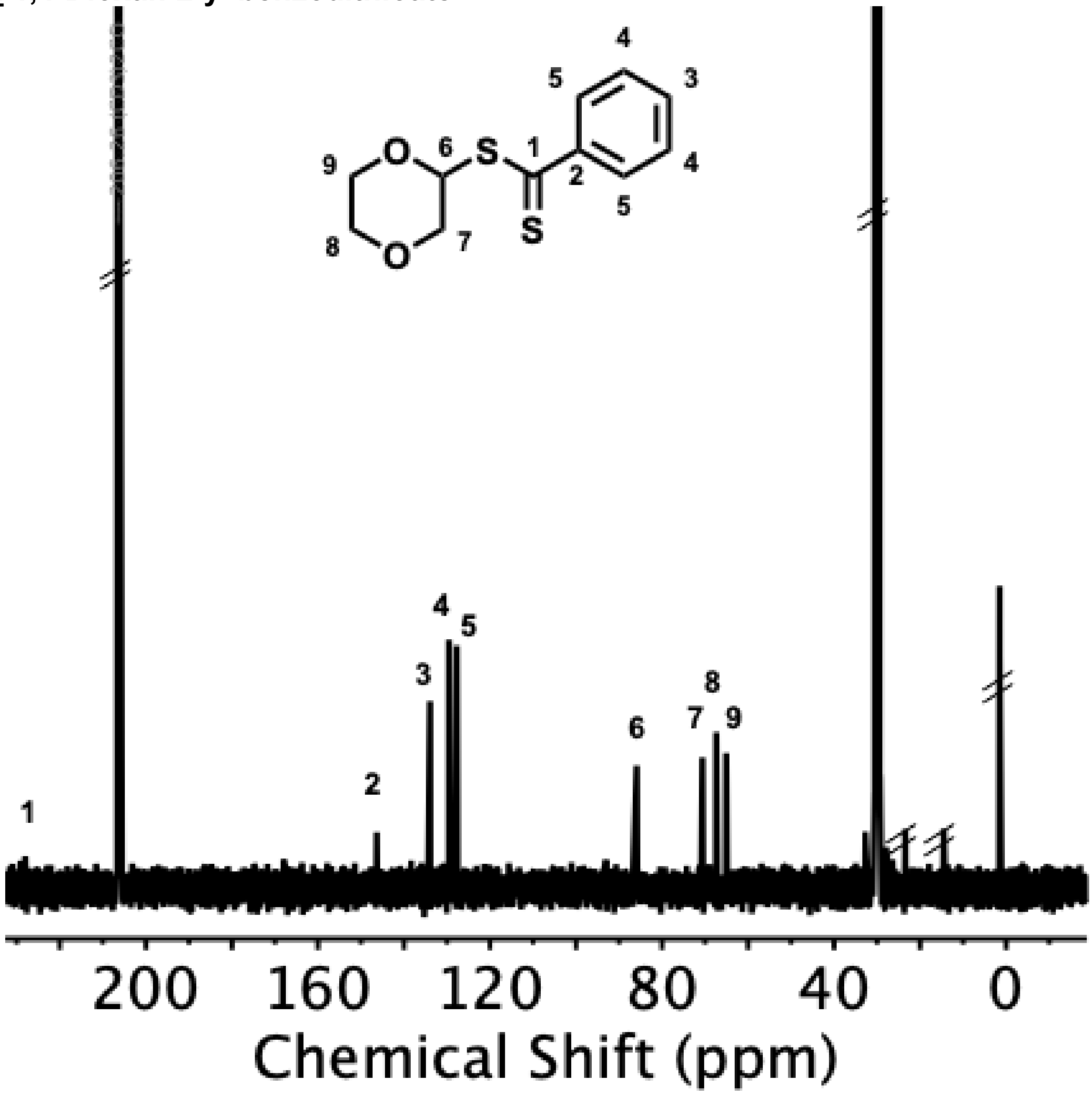

Figure S10. ^{13}C NMR of molecule **2** with indicated carbon atoms.

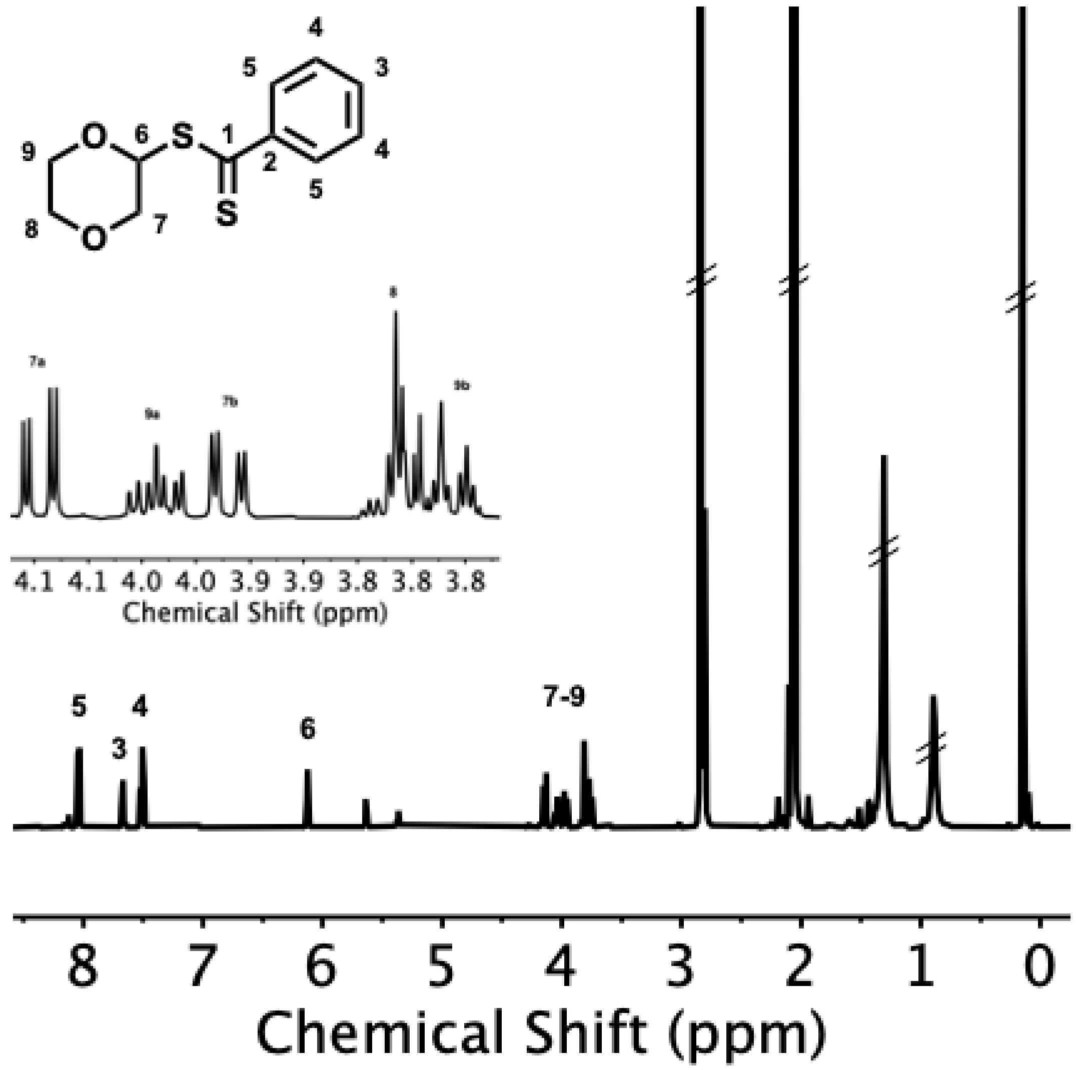

Figure S11. ^{1}H NMR of molecule **2** with indicated hydrogen atoms.

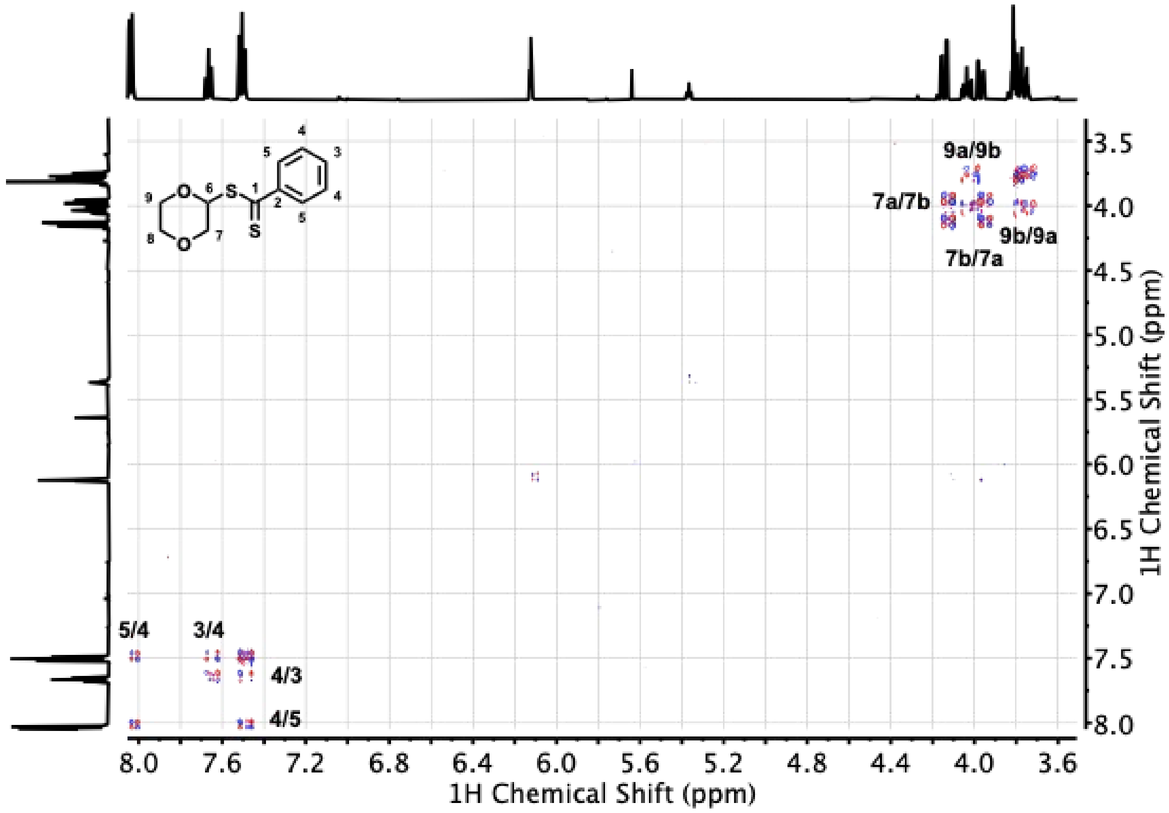

Figure S12. 2D NMR (COSY) of molecule **2** with indicated H-H correlations.

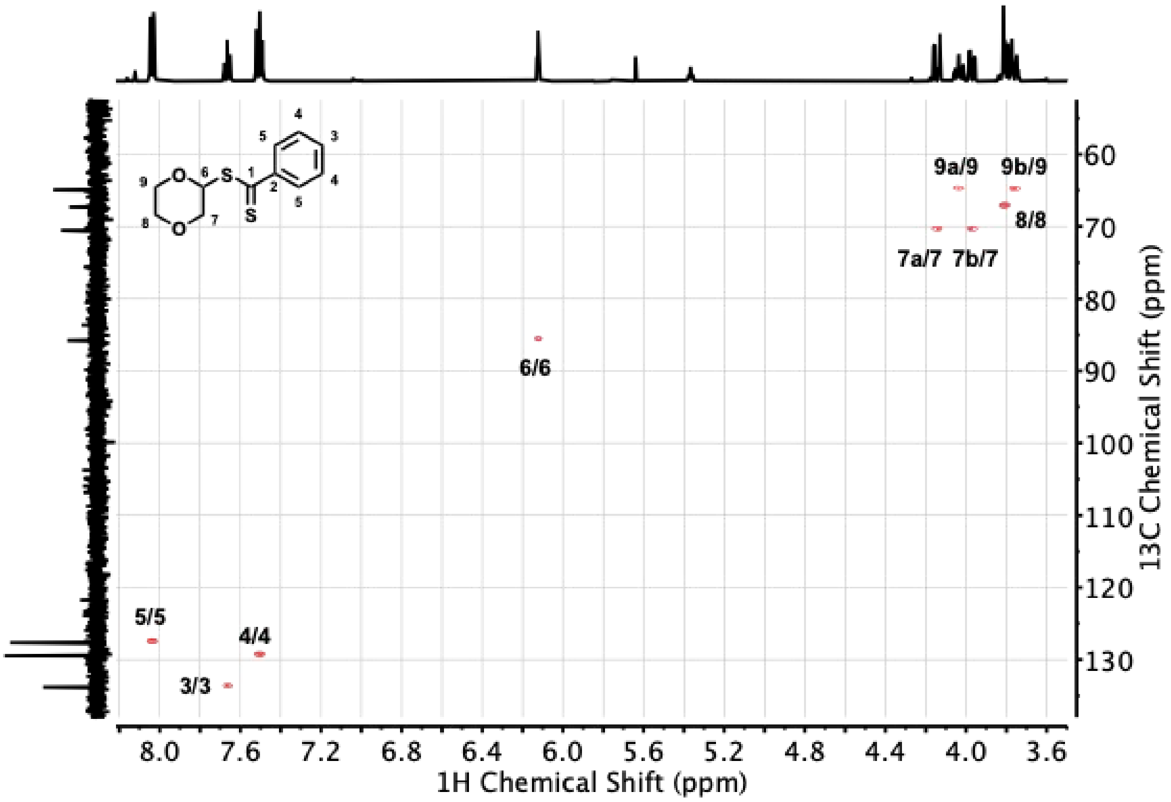

Figure S13. 2D NMR (HSQC) of molecule **2** with indicated H-C correlations.

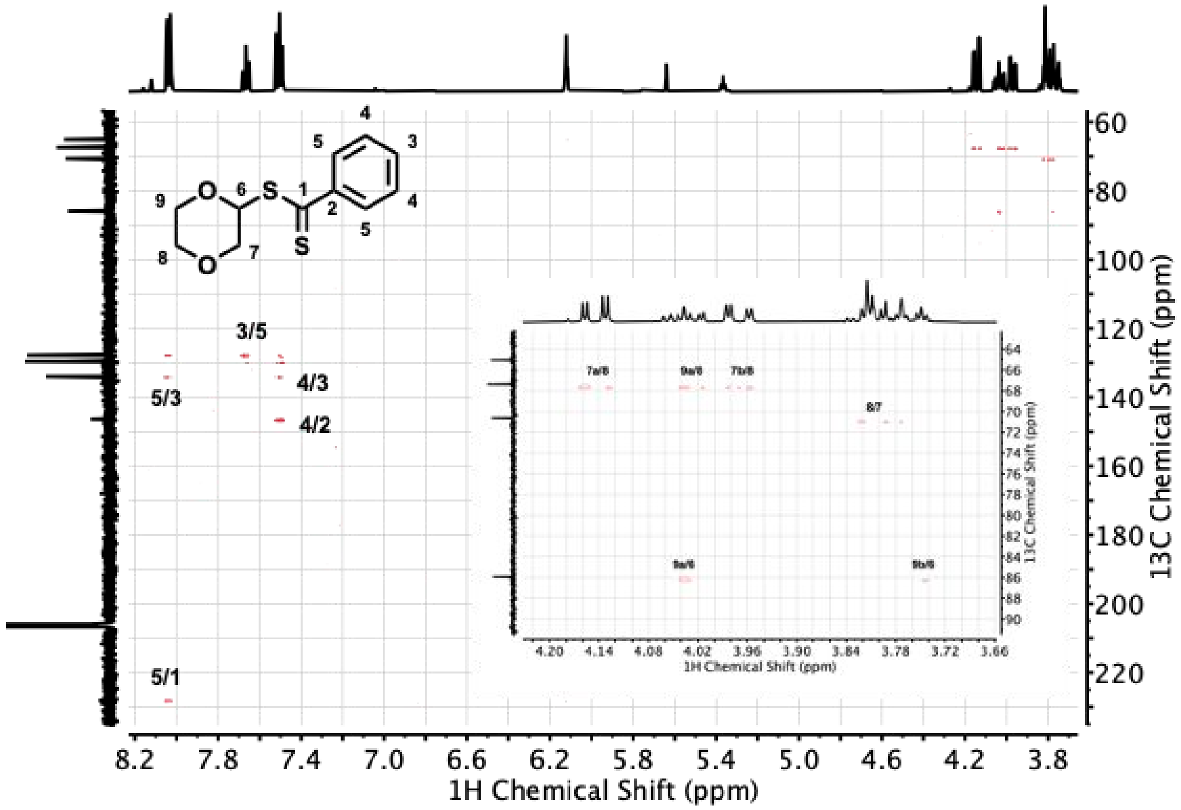

Figure S14. 2D NMR (HMBC) of molecule **2** with indicated H-C correlations.

2 1,4-Dioxan-2y-yl benzodithioate

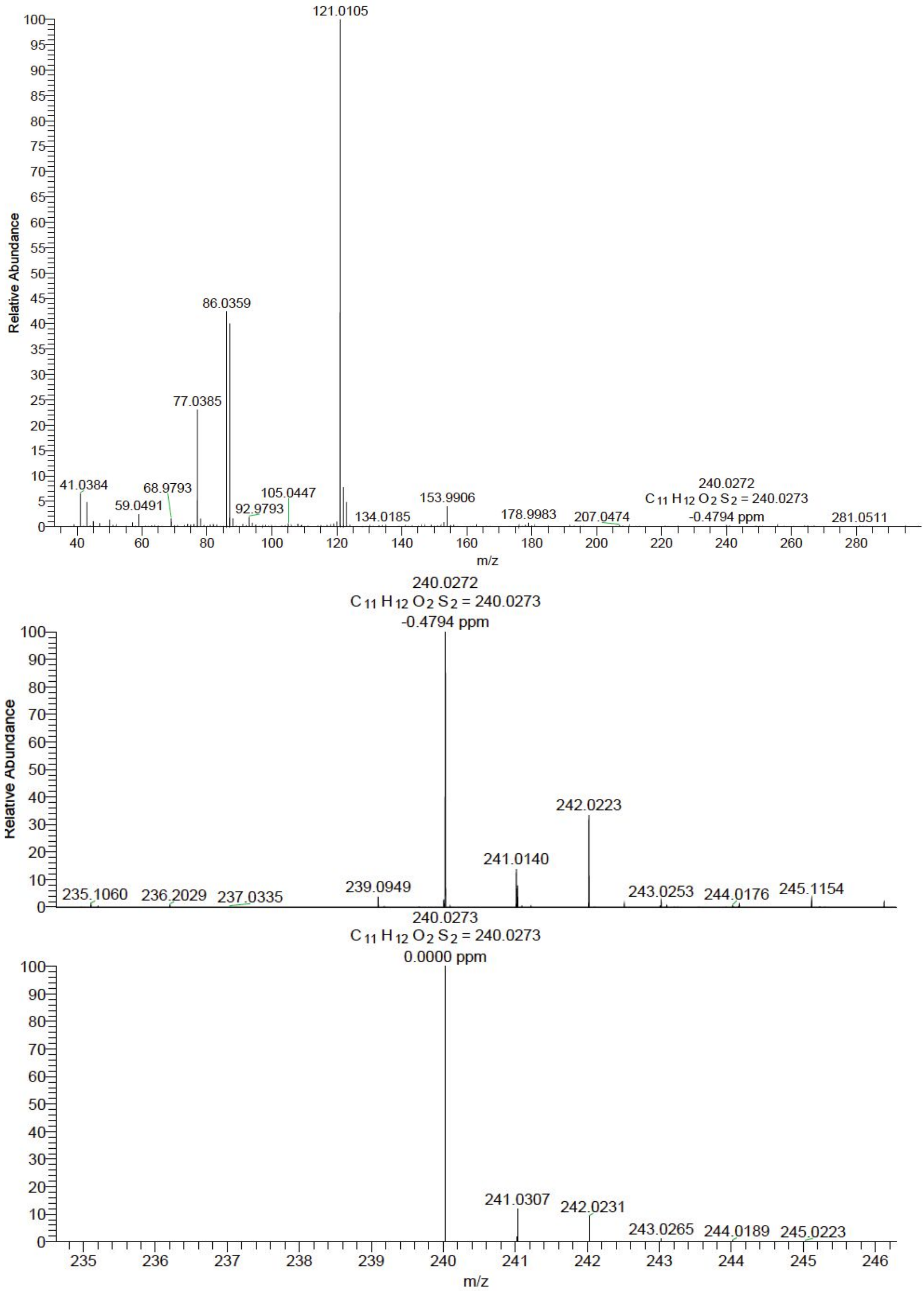

Figure S15. Full MS spectrum of molecule **2** (top), experimental isotope pattern (middle) and predicted isotope pattern (bottom).

<u>3</u> **(2-(Formyloxy)ethoxy)methyl benzodithioate**

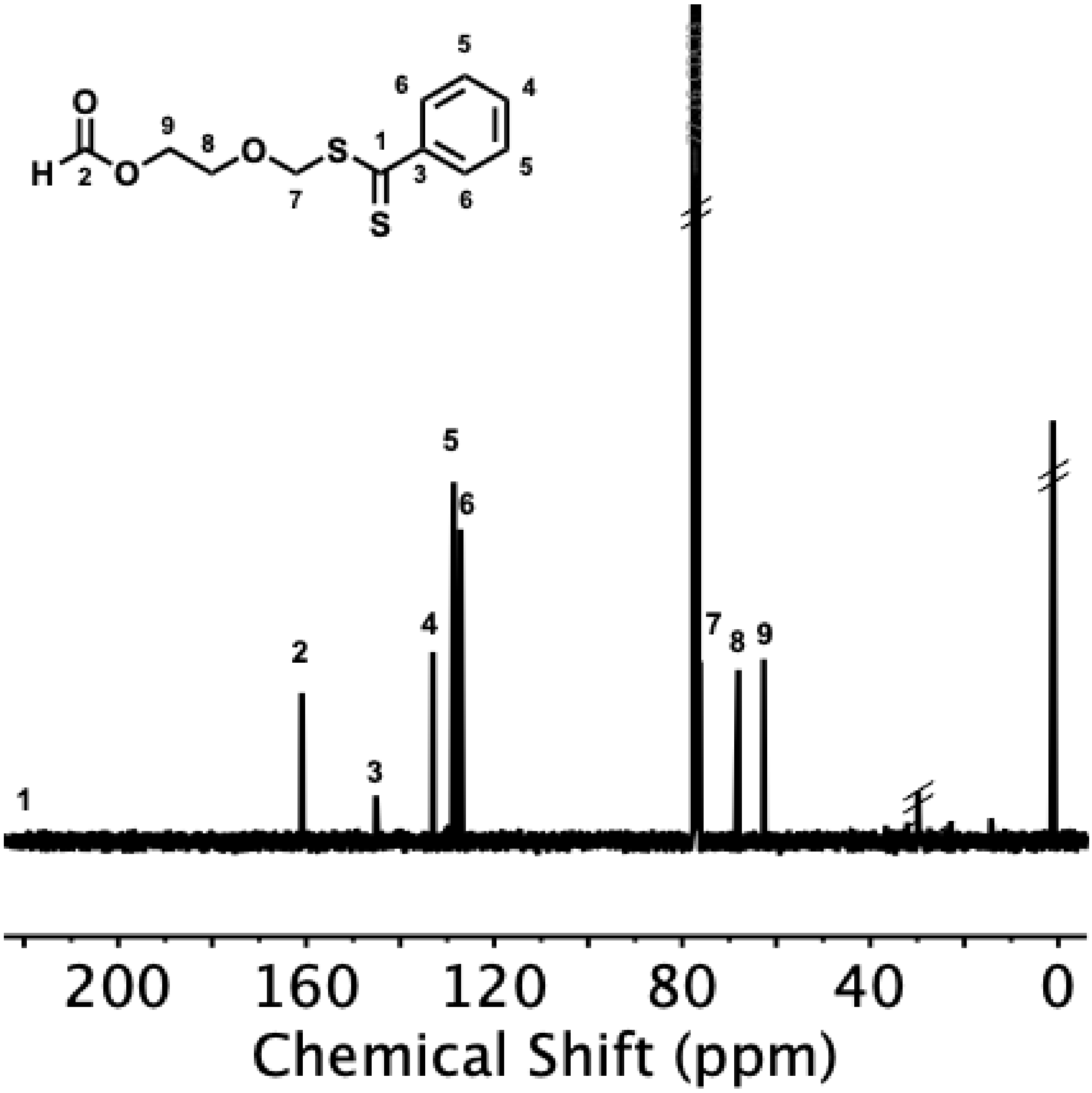

Figure S16. ^{13}C NMR of molecule **3** with indicated carbon atoms.

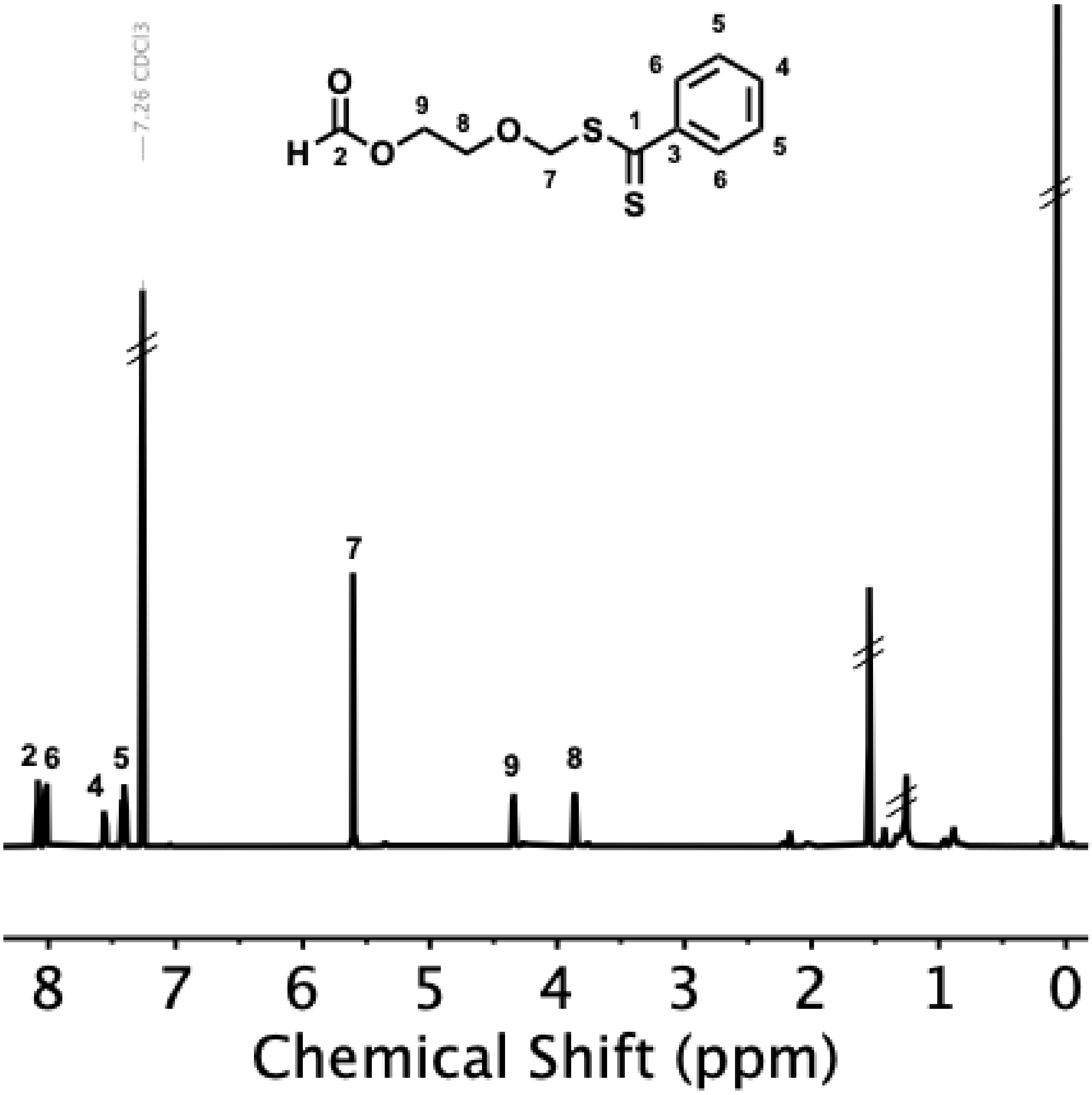

Figure S17. ^{1}H NMR of molecule **3** with indicated hydrogen atoms.

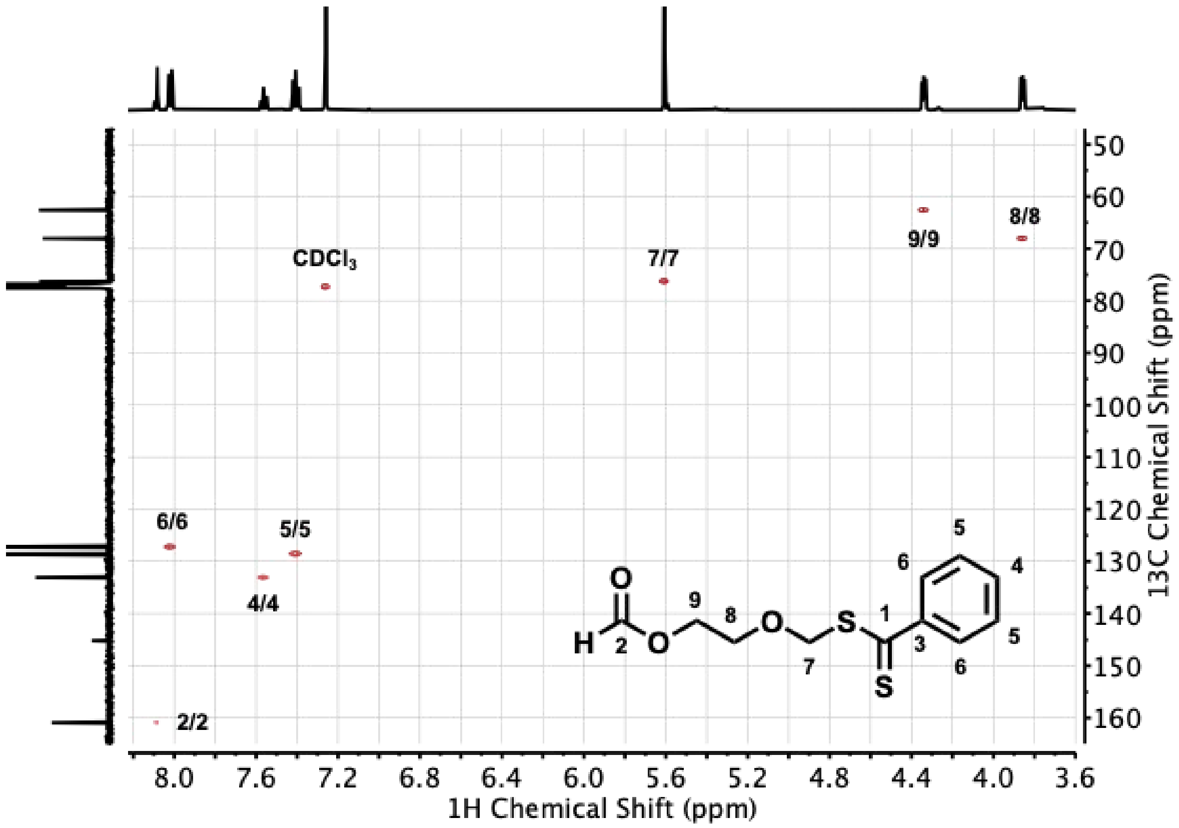

Figure S18. 2D NMR (HSQC) of molecule **3** with indicated H-C correlations.

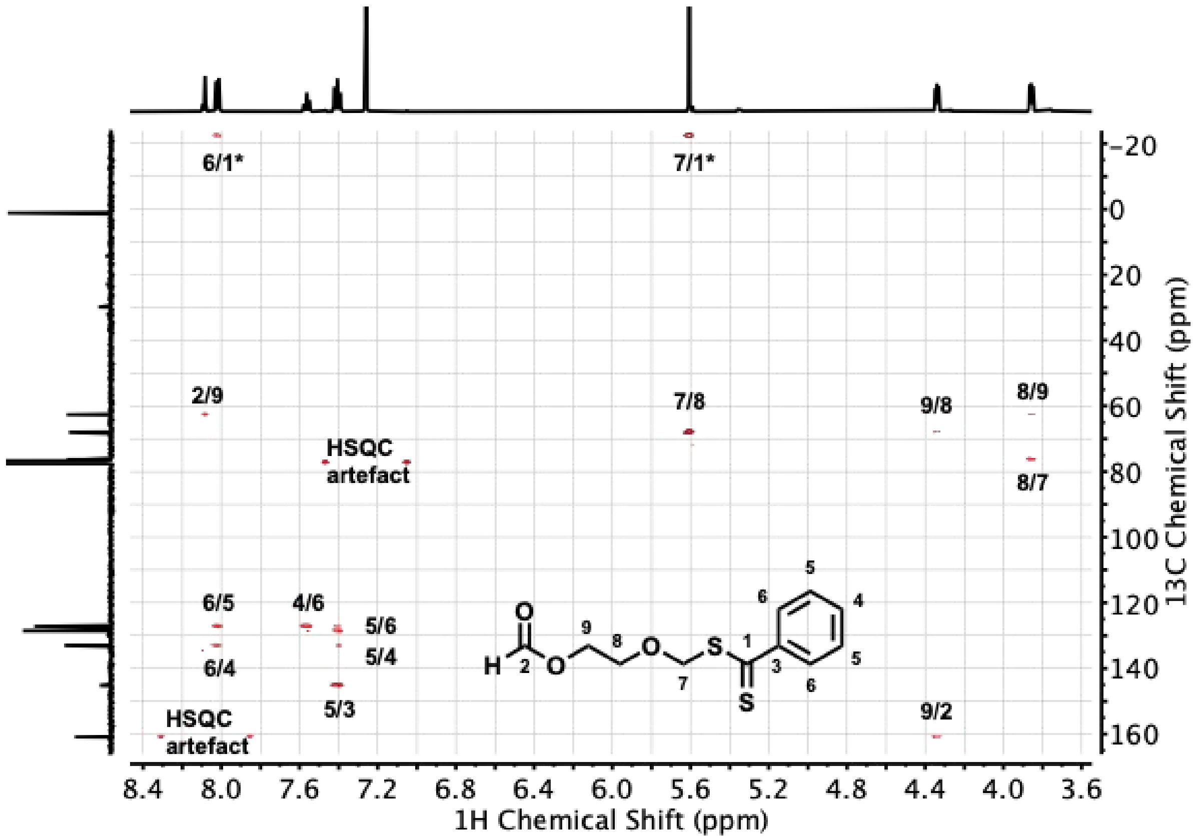

Figure S19. 2D NMR (HMBC) of molecule **3** with indicated H-C correlations.

<u>3</u> (2-(Formyloxy)ethoxy)methyl benzodithioate

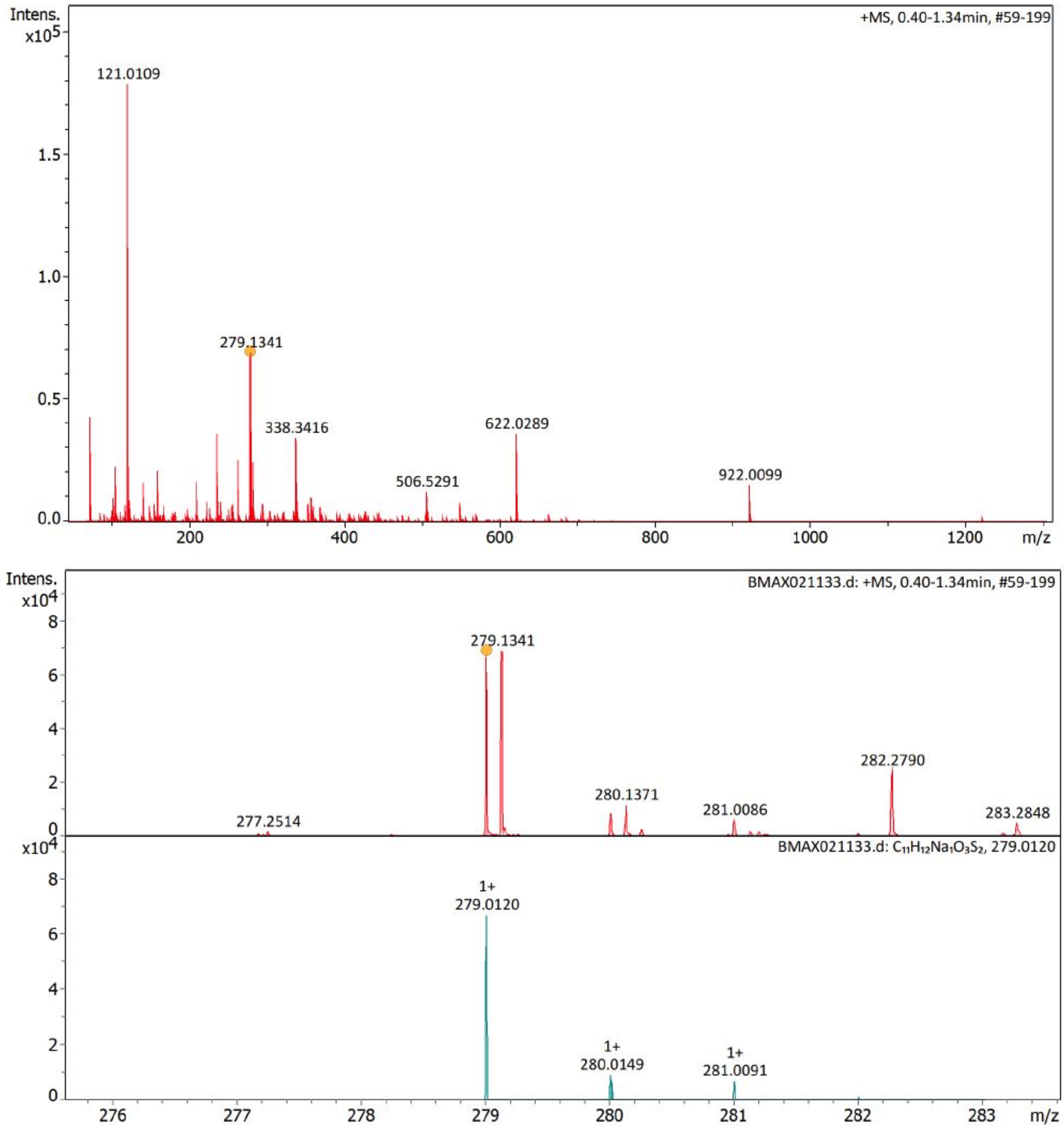

Figure S20. Full MS spectrum of molecule **3** (top), experimental isotope pattern (middle) and predicted isotope pattern (bottom).

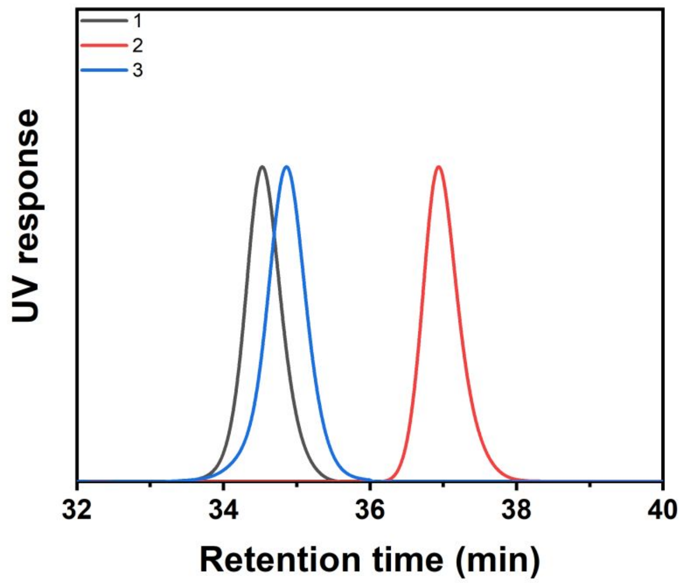

Figure S21. UV-SEC traces of molecules **1**, **2**, and **3**.

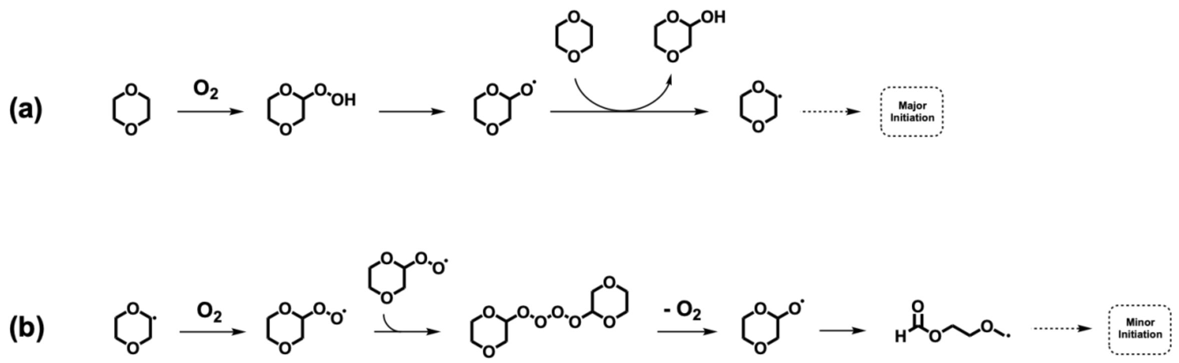

Figure S22. Formation of solvent-based radicals. (a) Proposed mechanism leading to 1,4-dioxanyl radical. (b) Proposed mechanism leading to 2-methoxyethyl formate radical.

Characteristic NMR signals

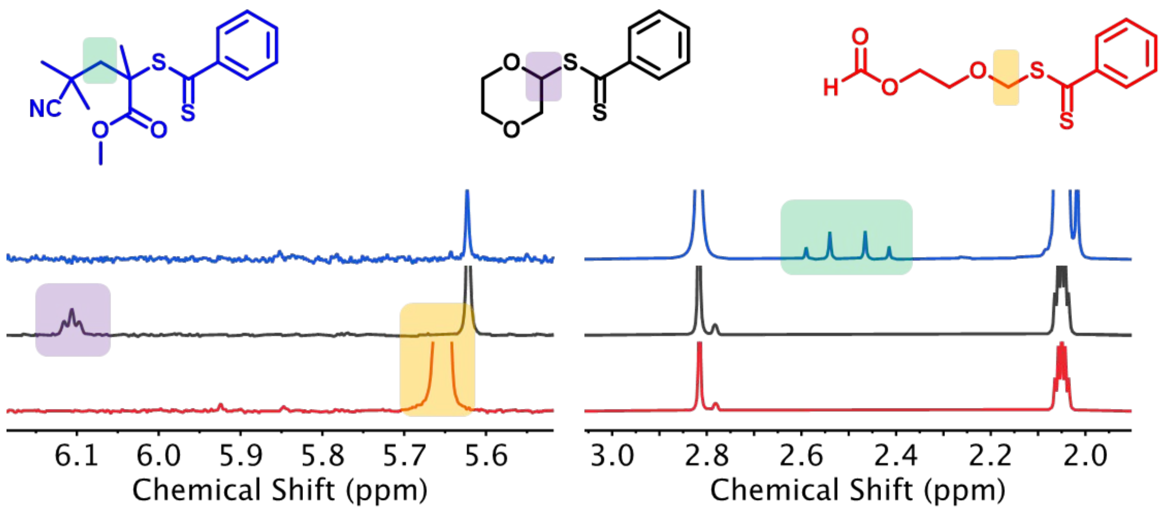

Figure S23. Characteristic ^{1}H NMR used for the calculation of the relative fraction of the end-group derived molecules..

3.3 PMMA-DTB 5 mM kinetics interval

Table S3. Kinetic Data on the depolymerization of PMMA-DTB in 1,4-dioxane at 5 mM and 120 °C. The Relative fraction ratios have been normalized to 1.

Time (min)	Conversion (%)	**1**	**2**	**3**
10	10	0.203	0.656	0.141
30	25	0.513	0.455	0.032
60	46	0.605	0.332	0.063
90	65	0.648	0.279	0.074
120	75	0.673	0.248	0.079
150	80	0.671	0.235	0.094
180	83	0.662	0.225	0.013
210	84	0.617	0.234	0.150
240	86	0.587	0.260	0.153
600	89	0.259	0.299	0.441

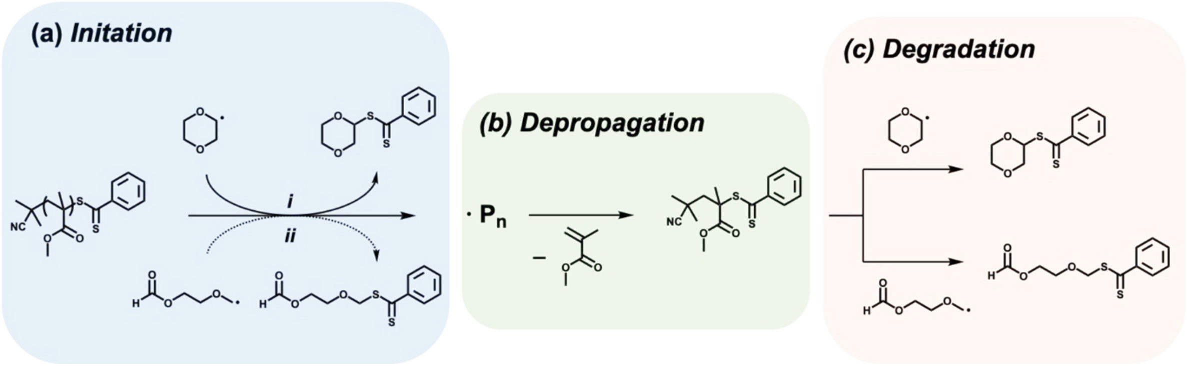

Figure S24. Proposed mechanism of solvent-initiated thermal RAFT depolymerization in 1,4-dioxane. (a) Initiation of the depolymerization achieved through 1,4-dioxane-based radicals with pathway *i* leading to **2** and pathway *ii* leading to **3**. (b) Depropagation generating monomer and unimer **1**. (c) Degradation of unimer **1** to form **2** and **3**.

4. Polymerization using depolymerization products

4.1 Polymerization of MMA using 1 as CTA

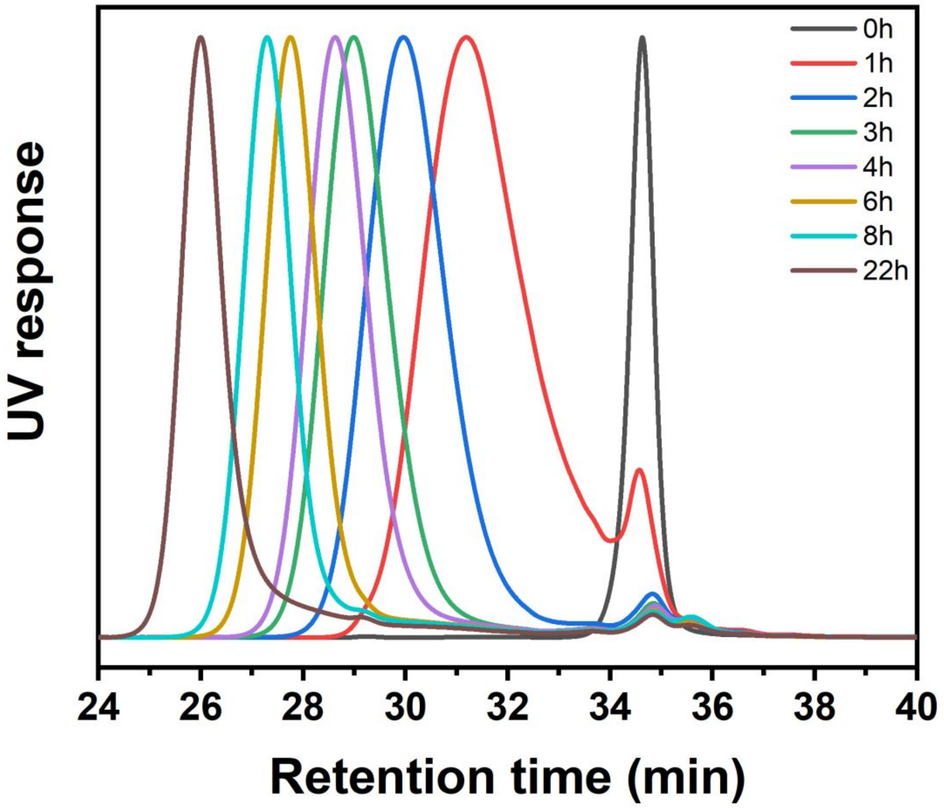

Figure S25. SEC trace (UV detector) of repolymerization using **1** as a CTA. Fast CTA consumption can be observed as well as incorporation of the chromophore in the resulting PMMA polymer.

Table S4. Characterization of repolymerization polymer using **1** as CTA. The data shows good agreement of the observed molecular weight with the theoretical M_n.

Entry	Monomer	[M]:[DTB]:[AIBN]	Time (h)	Conversion (%)	M_n^{theo} (g/mol)	M_n^{SEC} (g/mol)	$Đ$
1	MMA	608:1:0.1	1	3	2,100	2,400	1.17
2			2	7	4,600	4,100	1.16
3			3	11	7,000	6,900	1.14
4			4	14	8,800	8,400	1.13
5			6	22	13,700	13,800	1.10
6			8	27	16,800	17,500	1.11
7			22	60	36,800	34,500	1.10

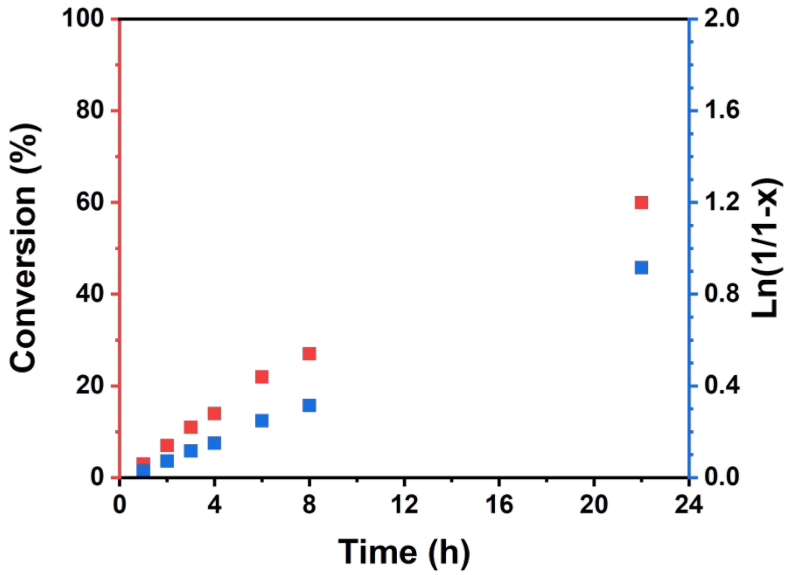

Figure S26. Plot of monomer conversion and ln(1/(1-x)) versus time during the polymerization of MMA with **1** as the CTA.

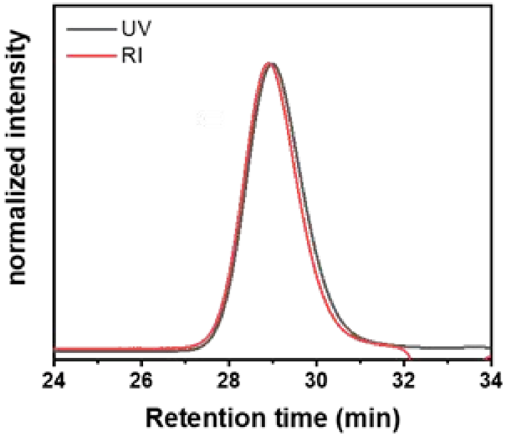

Figure S27. UV and RI SEC traces after 3 h of MMA polymerization in the presence of **1** as the CTA.

4.2 Polymerization of MMA using CPDB as CTA

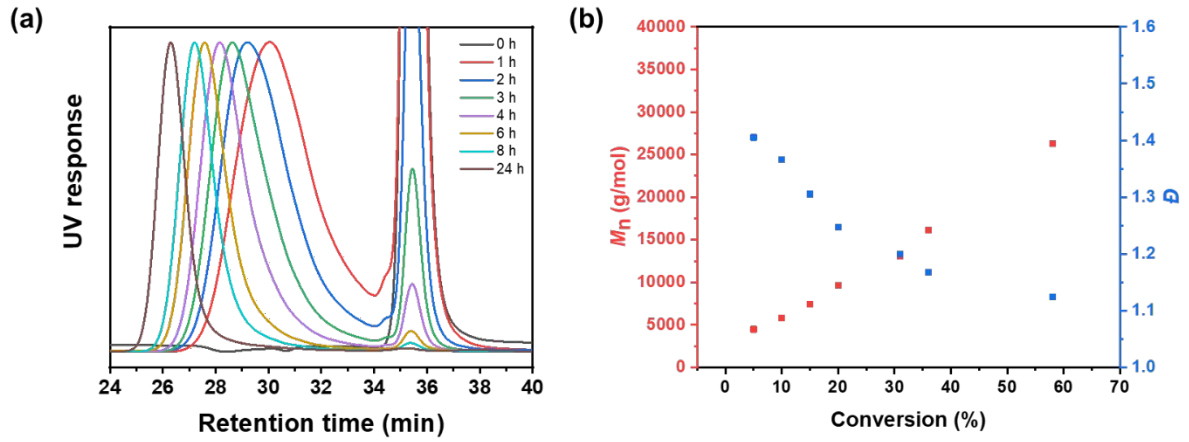

Figure S28. (a) SEC trace (UV detector) of repolymerization using CPDB as a CTA. (b) Plot of M_n and dispersity as a function of conversion. ([CTA]:[MMA]:[AIBN] = 1:500:0.1)

4.3 Polymerization of MMA in the presence of 2

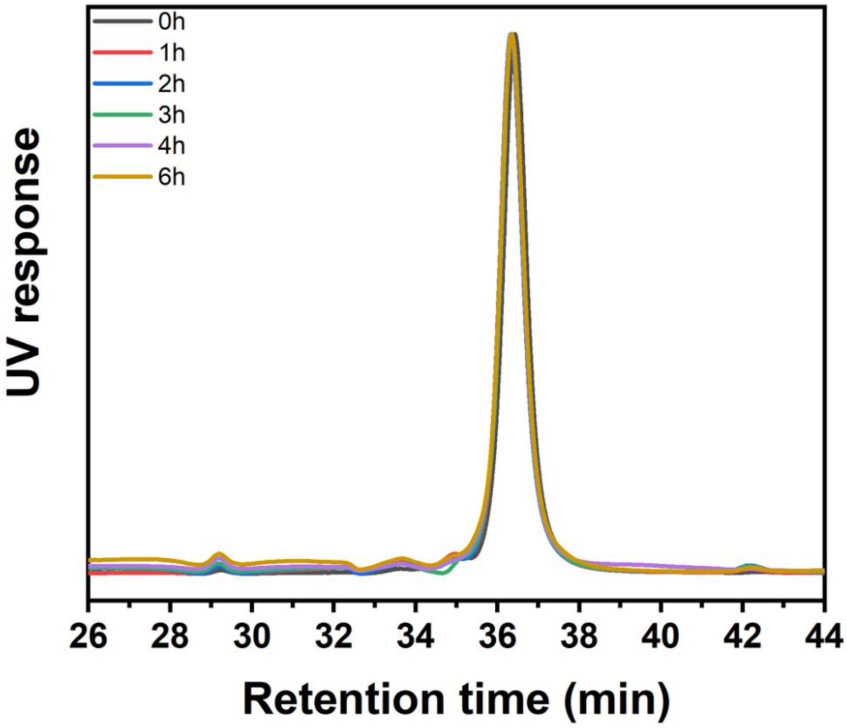

Figure S29. SEC trace (UV detector) of MMA polymerization in the presence of **2.**

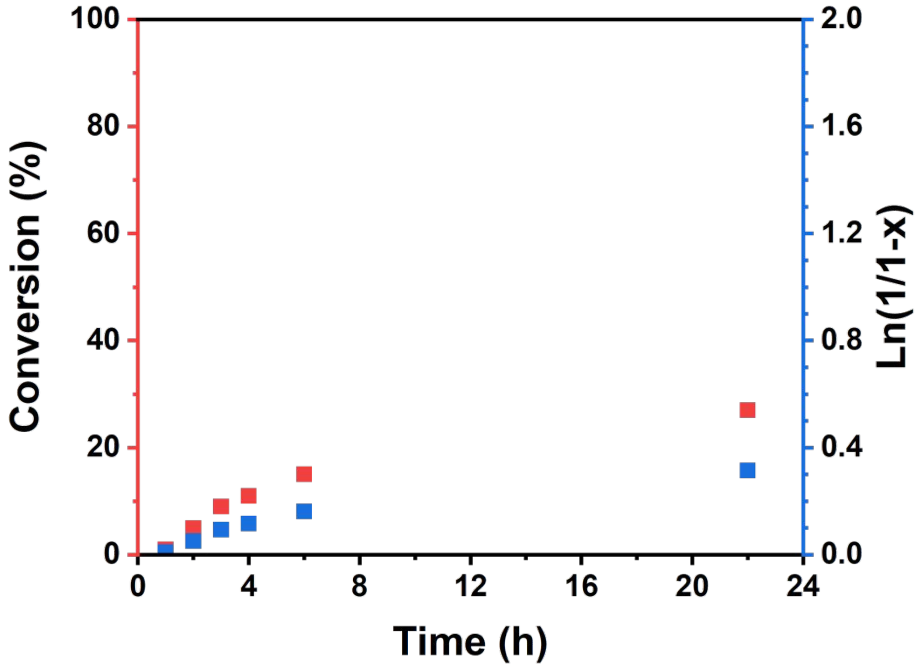

Figure S30. Plot of monomer conversion and ln(1/(1-x)) versus time during the polymerization of MMA in the presence of **2**.

Table S5. Characterization of repolymerization polymer in the presence of **2**.

Entry	Monomer	[M]:[DTB]:[AIBN]	Time (h)	Conversion (%)	M_n^{theo} (g/mol)	M_n^{SEC} (g/mol)	*Đ*
1	MMA	707:1:0.1	2	4	3,400	116,700	1.43
2	MMA	707:1:0.1	3	9	6,900	155,700	1.47
3	MMA	707:1:0.1	4	11	8,300	156,400	1.45
4	MMA	707:1:0.1	6	15	10,500	153,800	1.49

4.4 Polymerization of MMA in the presence of 3

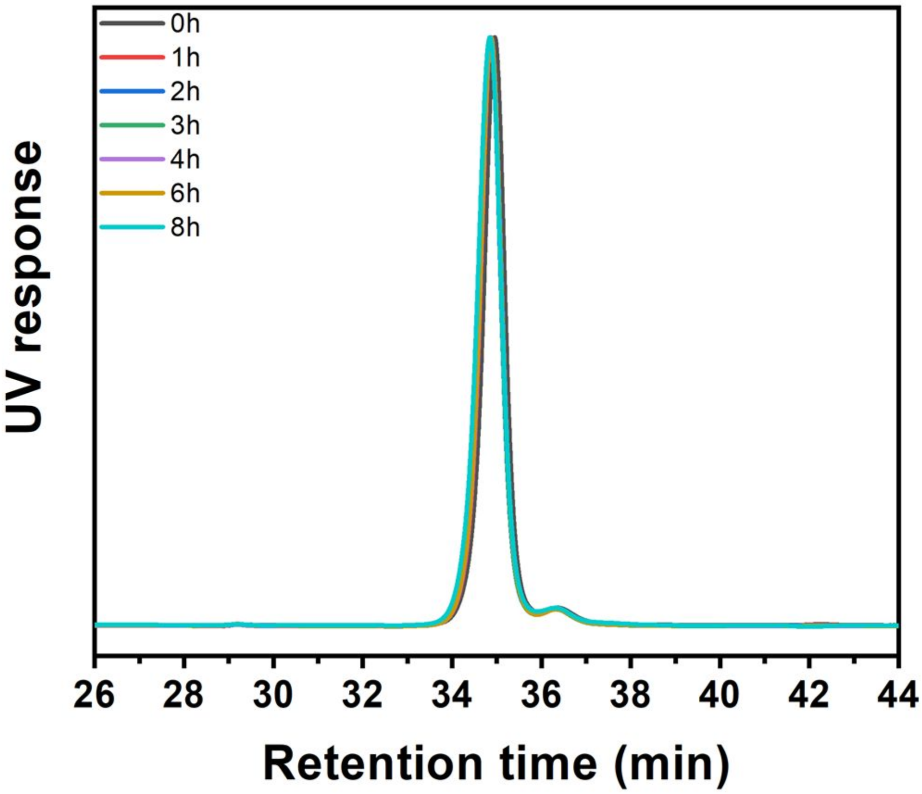

Figure S31. SEC trace (UV detector) of MMA polymerization in the presence of **3.**

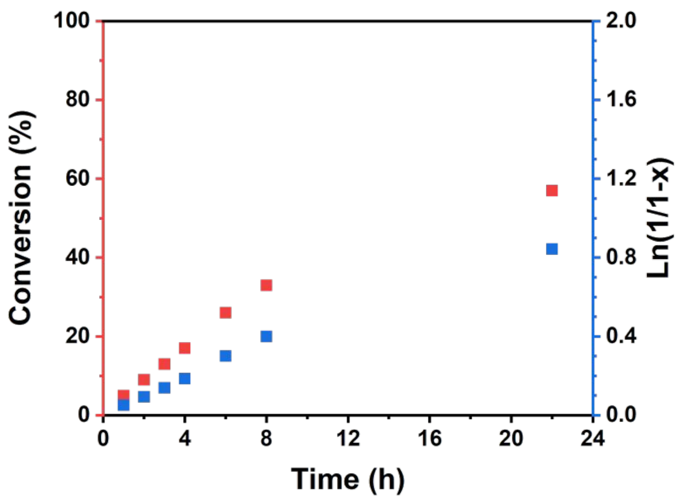

Figure S32. Plot of monomer conversion and ln(1/(1-x)) versus time during the polymerization of MMA in the presence of **3**.

Table S6. Characterization of repolymerization polymer in the presence of **3**.

Entry	Monomer	[M]:[DTB]:[AIBN]	Time (h)	Conversion (%)	M_n^{theo} (g/mol)	M_n^{SEC} (g/mol)	$Đ$
1	MMA	591:1:0.1	1	5	3,200	170,400	1.55
2	MMA	591:1:0.1	2	9	5,700	192,300	1.47
3	MMA	591:1:0.1	3	13	8,200	203,000	1.56
4	MMA	591:1:0.1	4	17	10,400	215,700	1.63
5	MMA	591:1:0.1	6	26	15,700	222,100	1.50
6	MMA	591:1:0.1	8	33	19,900	259,700	1.59

4.5 Free Radical Polymerization of MMA

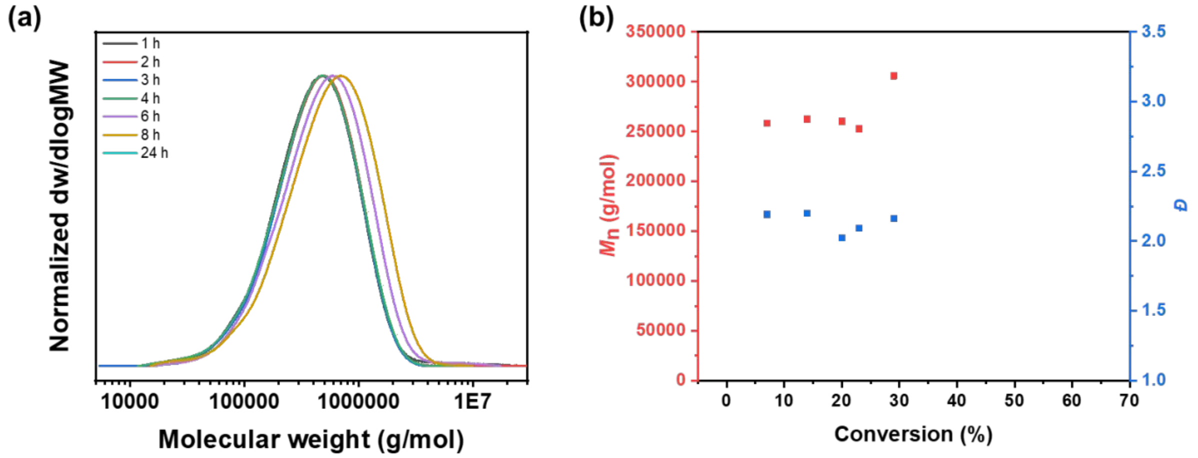

Figure S33. (a) SEC trace (RI detector) and (b) Plot of M_n and dispersity versus monomer conversion during the free radical polymerization of MMA.

5. Polymerization & Depolymerization of PMMA-TTC

5.1 Synthesis of PMMA-TTC

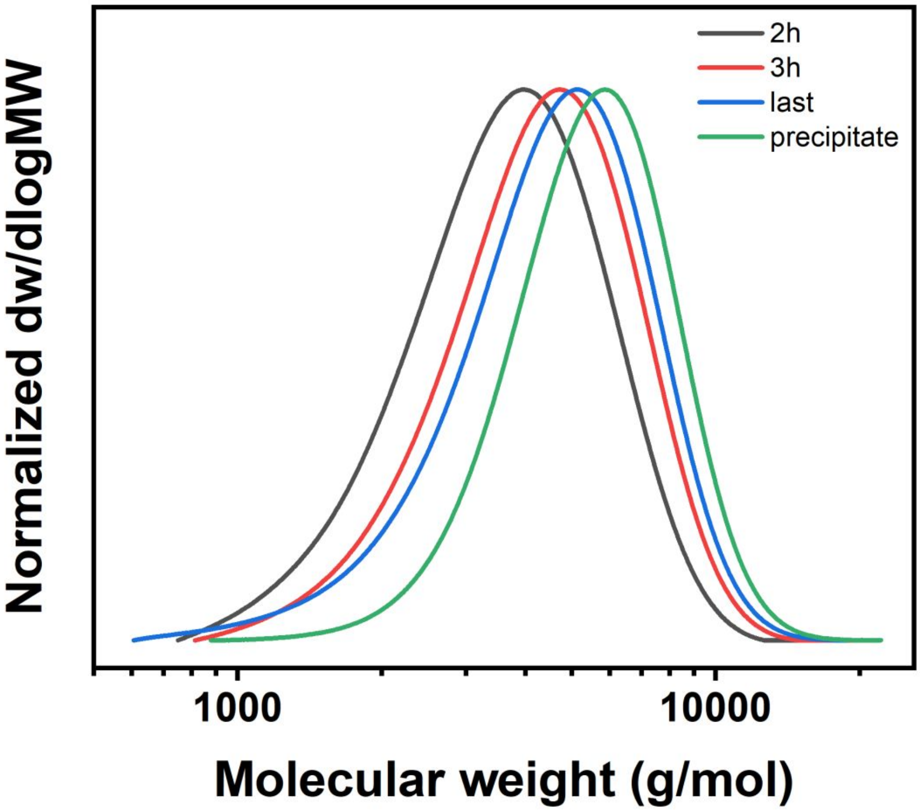

Figure S34. SEC trace of PMMA synthesized via RAFT polymerization with TTC as the chain transfer agent ([MMA]:[CTA]:[AIBN] = 50:1:0.1).

Table S7. Characterization of PMMA-TTC.

Entry	Monomer	[M]:[DTB]:[AIBN]	Time (h)	Conversion (%)	M_n^{theo} (g/mol)	M_n^{SEC} (g/mol)	*Đ*
1	MMA	50:1:0.1	4	54	3,000	5,600	1.14

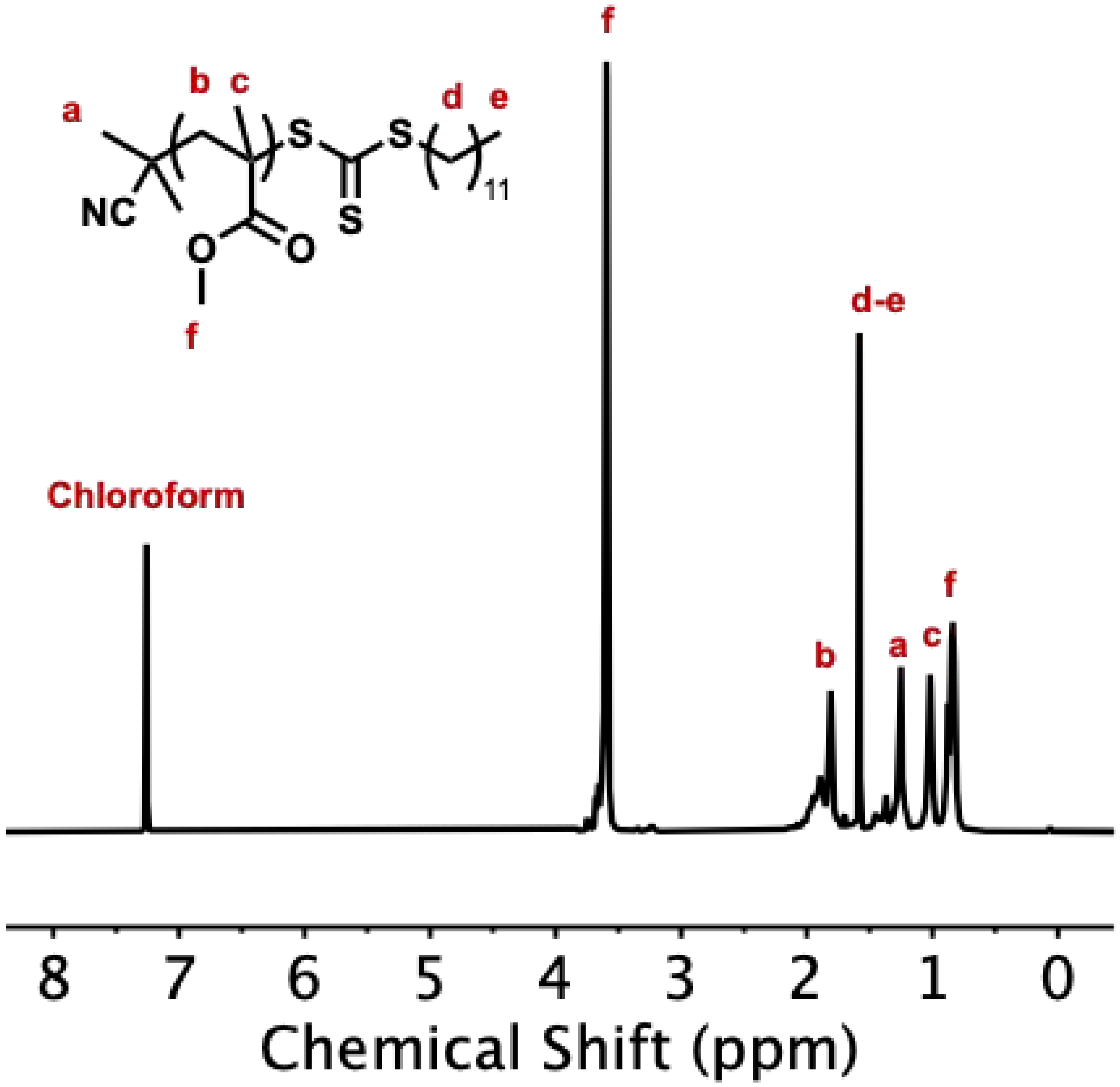

Figure S35. ^{1}H-NMR spectrum of purified PMMA-TTC prior to depolymerization.

5.2 Depolymerization of PMMA-TTC

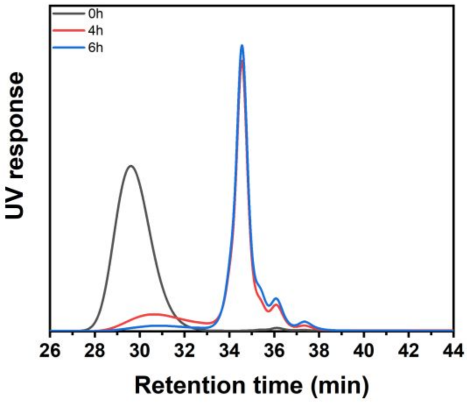

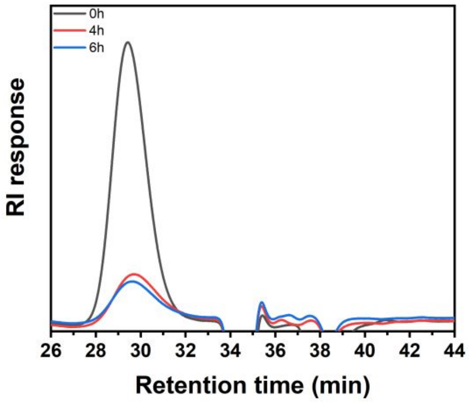

Figure S36. SEC trace of PMMA-TTC depolymerization at 120 °C at 25 mM. UV-Vis (left) and RI (right).

Table S8. Characterization of PMMA-TTC depolymerization.

Entry	Conc. (mM)	Temp. (°C)	Time (h)	Conversion (%)
1	25	120	6	80

4 Methyl 4-cyano-2-(((dodecylthio)carbonothioyl)thio)-2,4-dimethlypentanoate

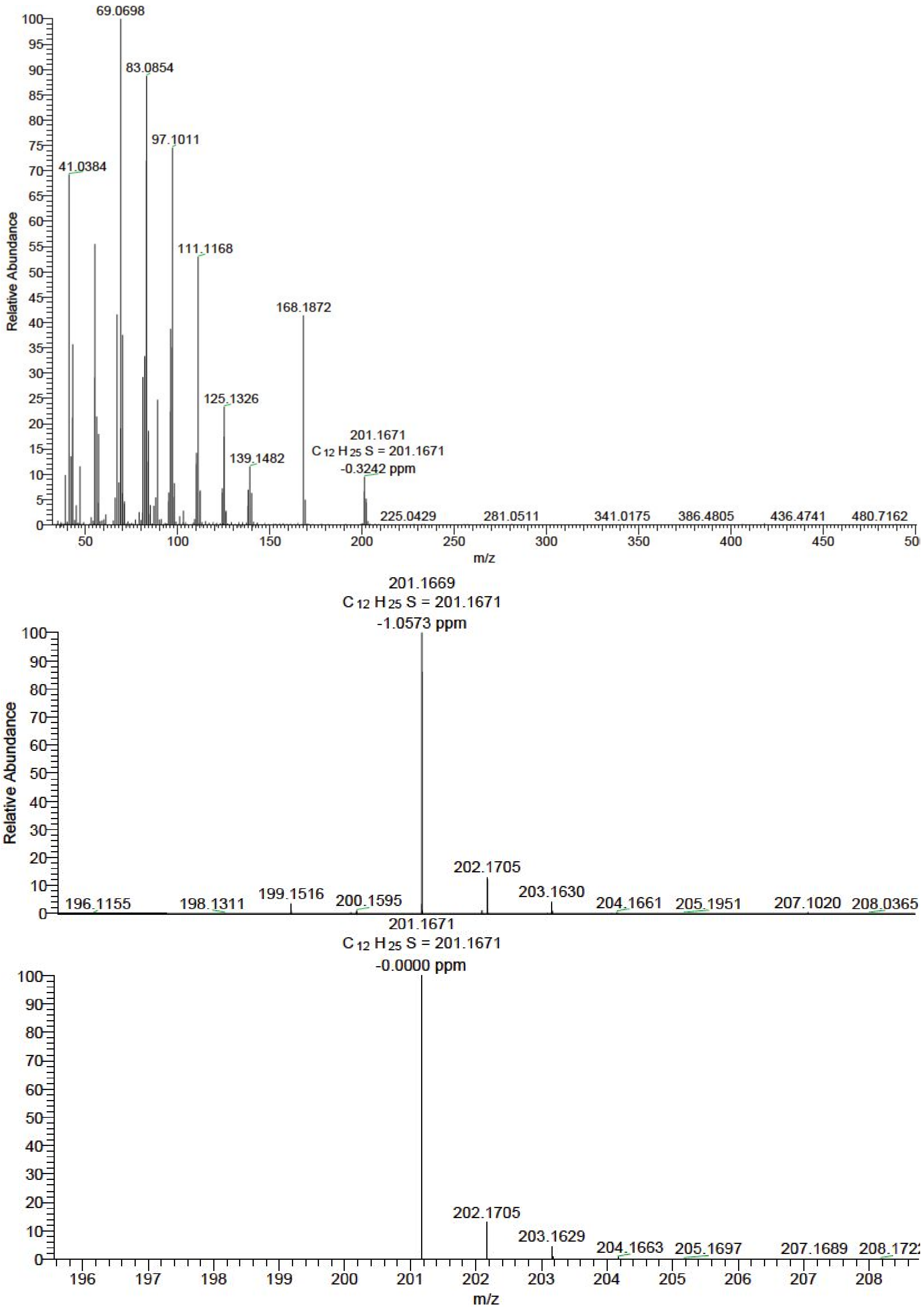

Figure S37. Full MS spectrum of molecule **4** (top), experimental isotope pattern (middle) and predicted isotope pattern (bottom).

5 1,4-Dioxan-2-yl dodecyl carbonotrithioate

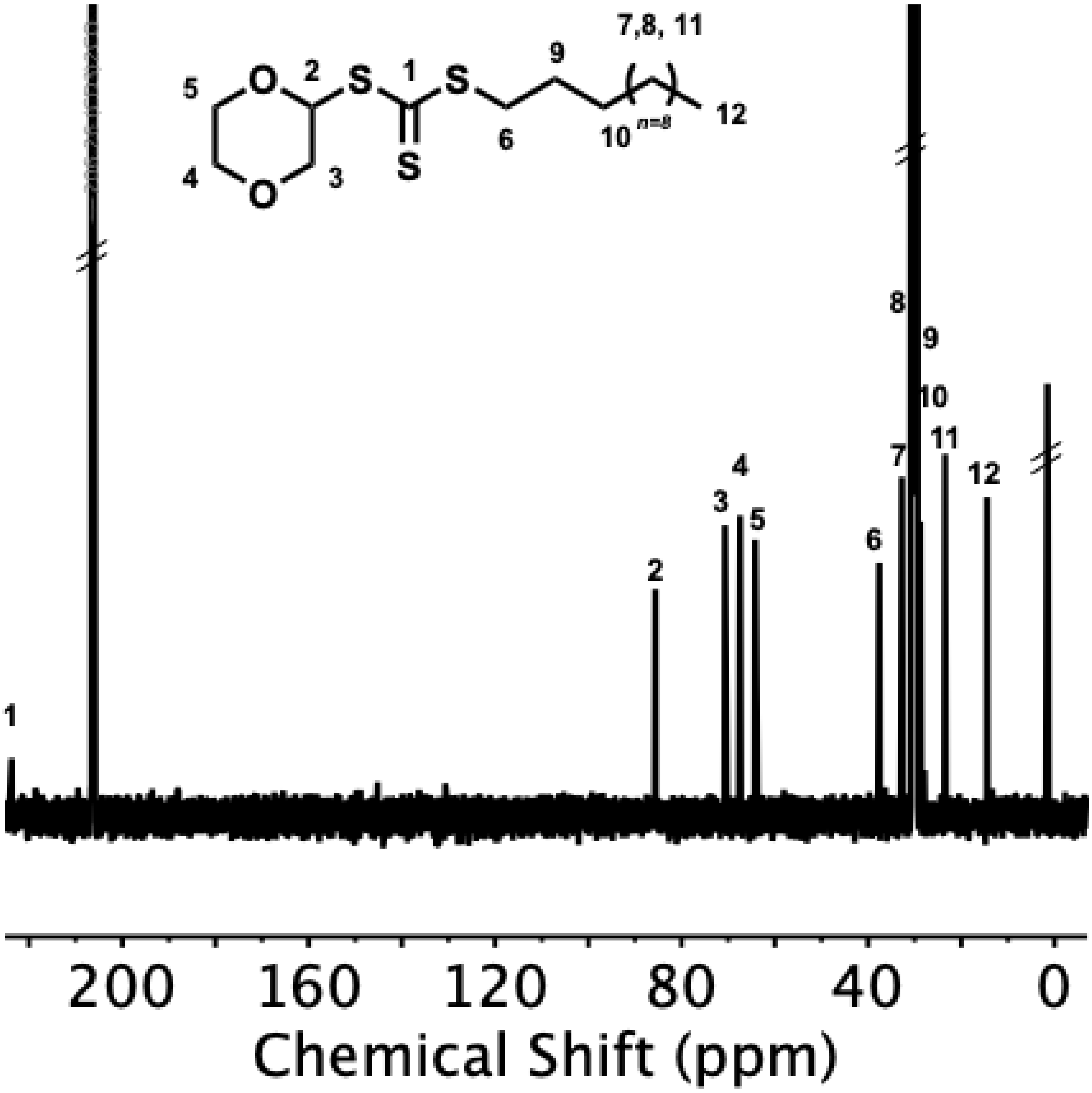

Figure S38. ^{13}C NMR of molecule **5** with indicated carbon atoms.

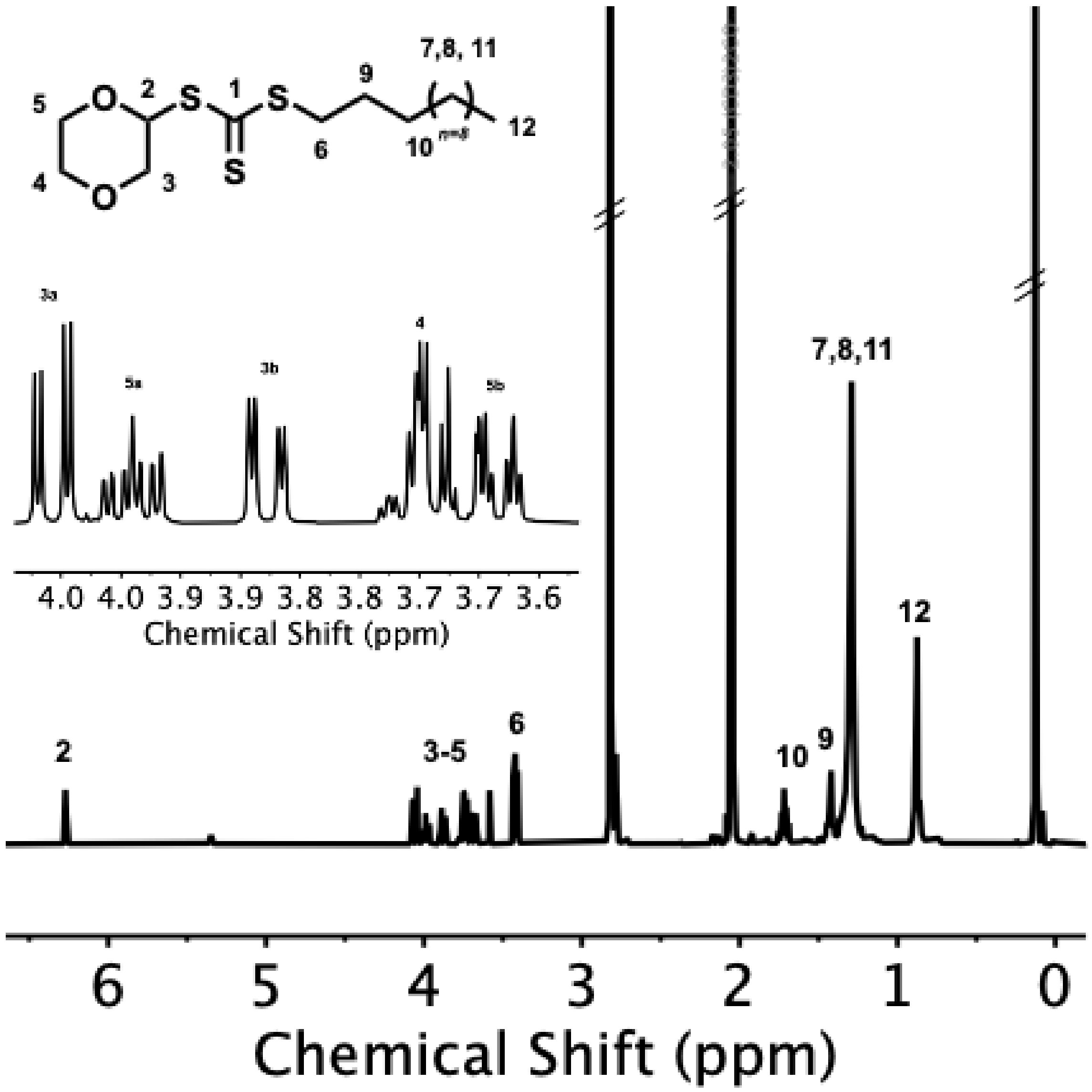

Figure S39. ^{1}H NMR of molecule **5** with indicated hydrogen atoms.

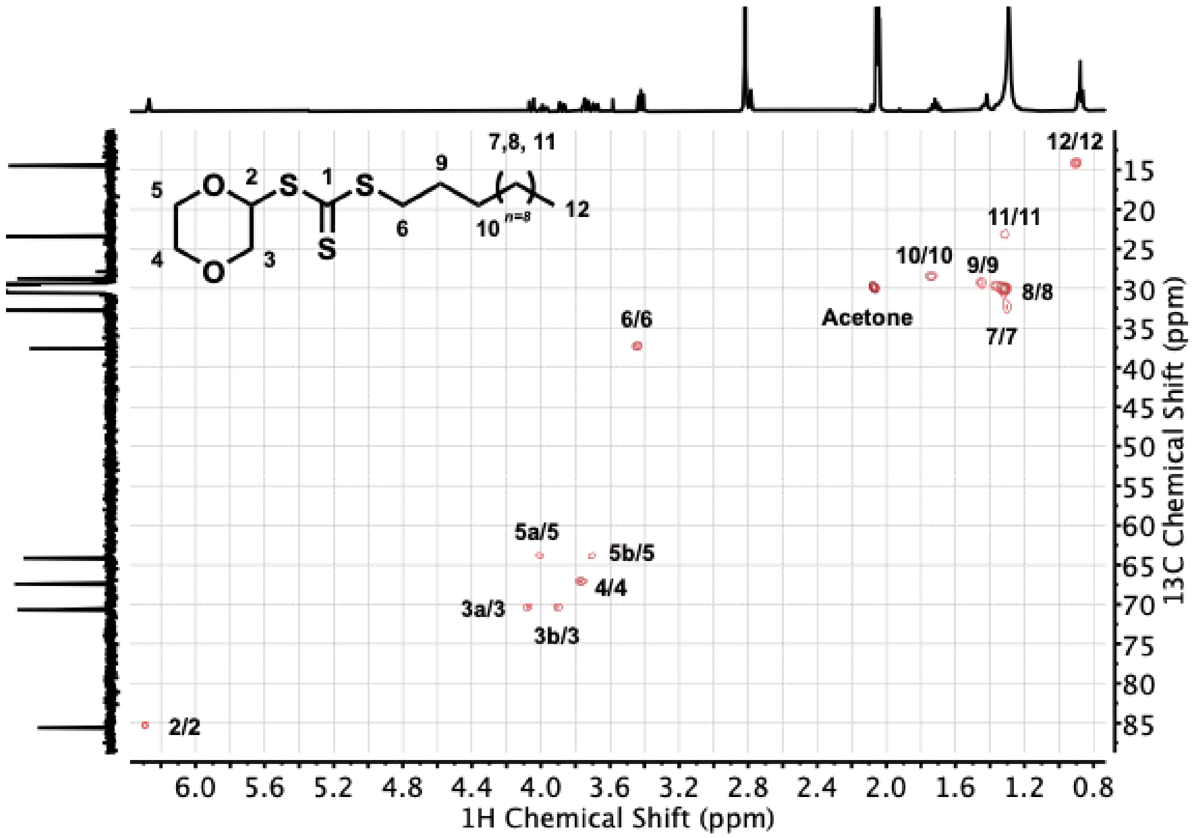

Figure S40. 2D NMR (HSQC) of molecule **5** with indicated H-C correlations.

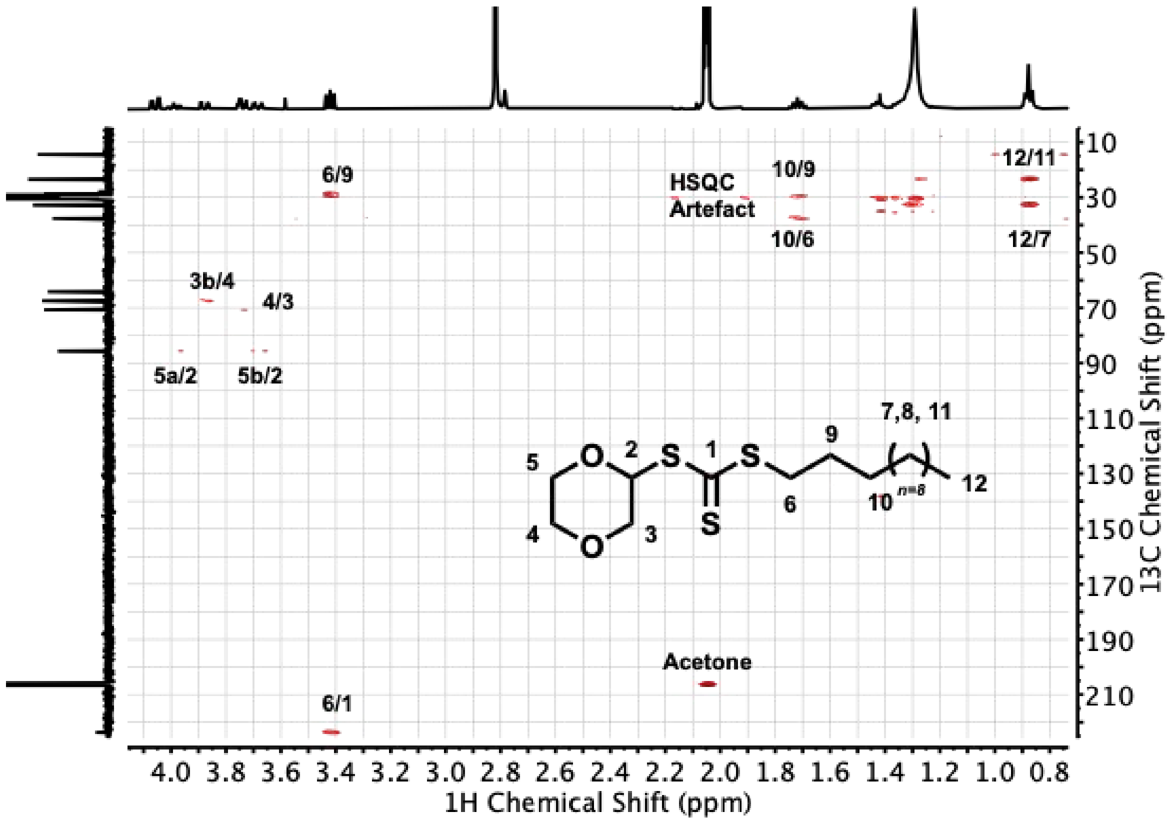

Figure S41. 2D NMR (HMBC) of molecule **5** with indicated H-C correlations.

5 **1,4-Dioxan-2-yl dodecyl carbonotrithioate**

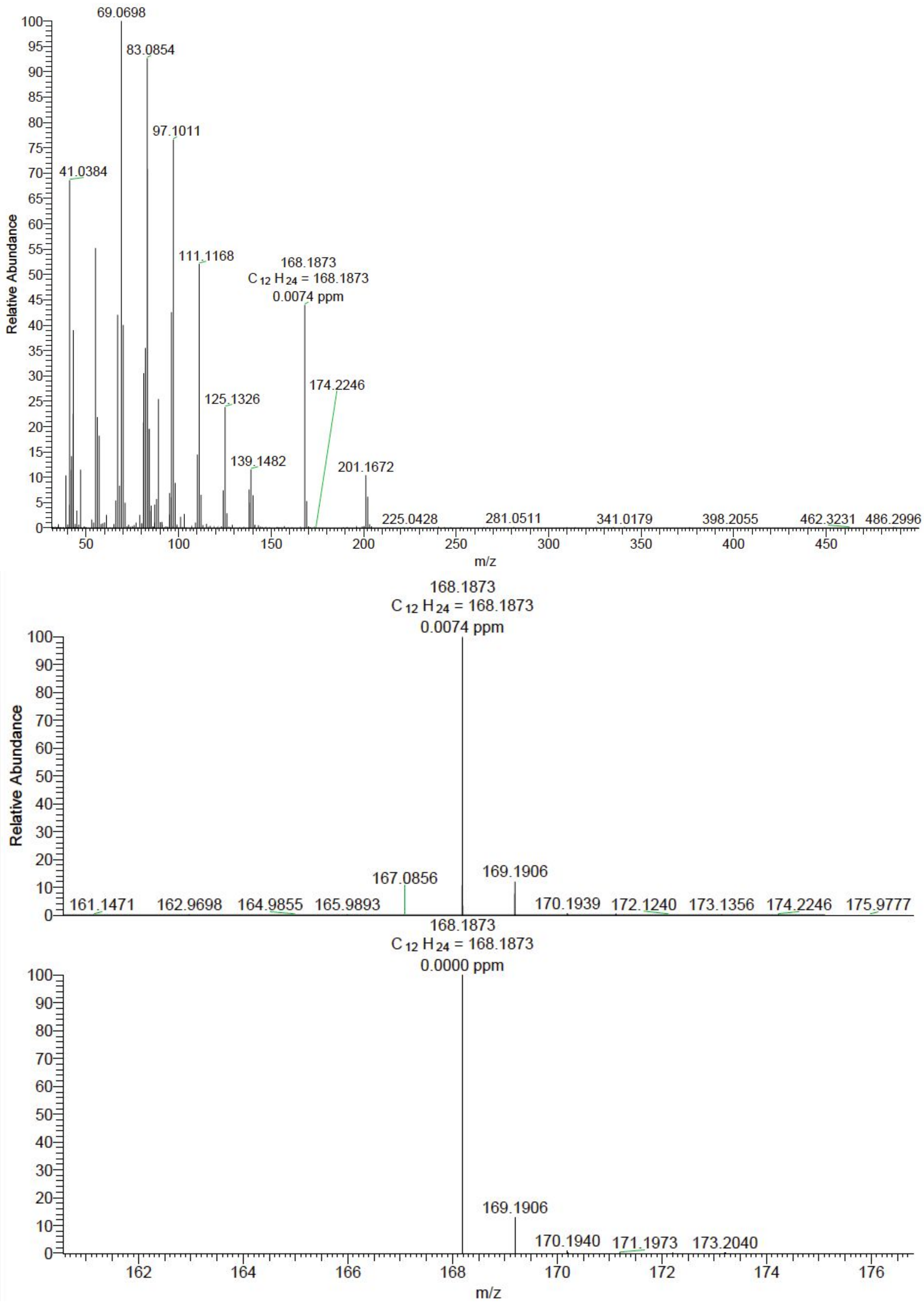

Figure S42. Full MS spectrum of molecule **5** (top), experimental isotope pattern (middle) and predicted isotope pattern (bottom).

6 2-((((dodecylthio)carbonothioyl)thio)methocy)ethyl formate

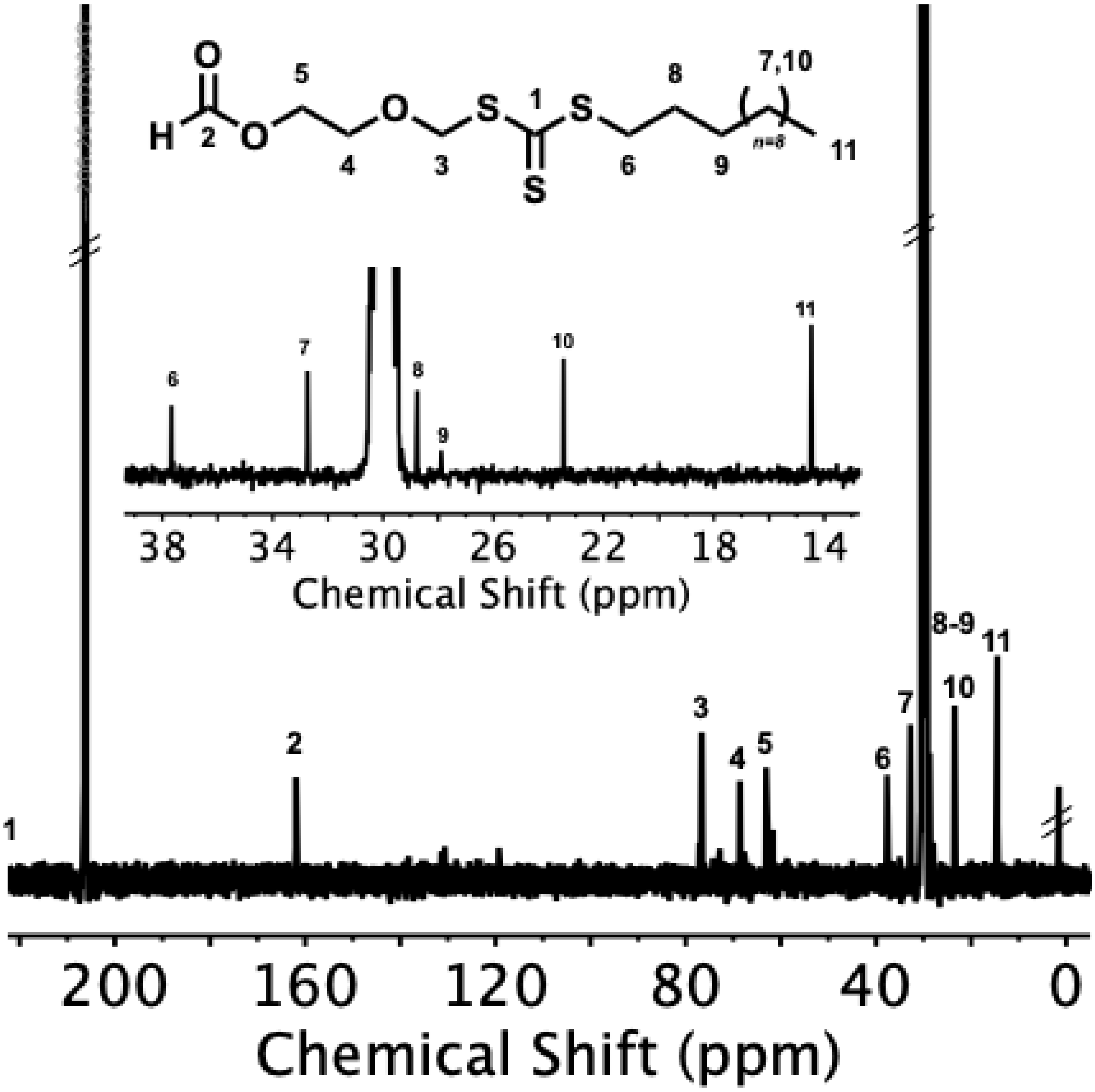

Figure S43. ^{13}C NMR of molecule **6** with indicated carbon atoms.

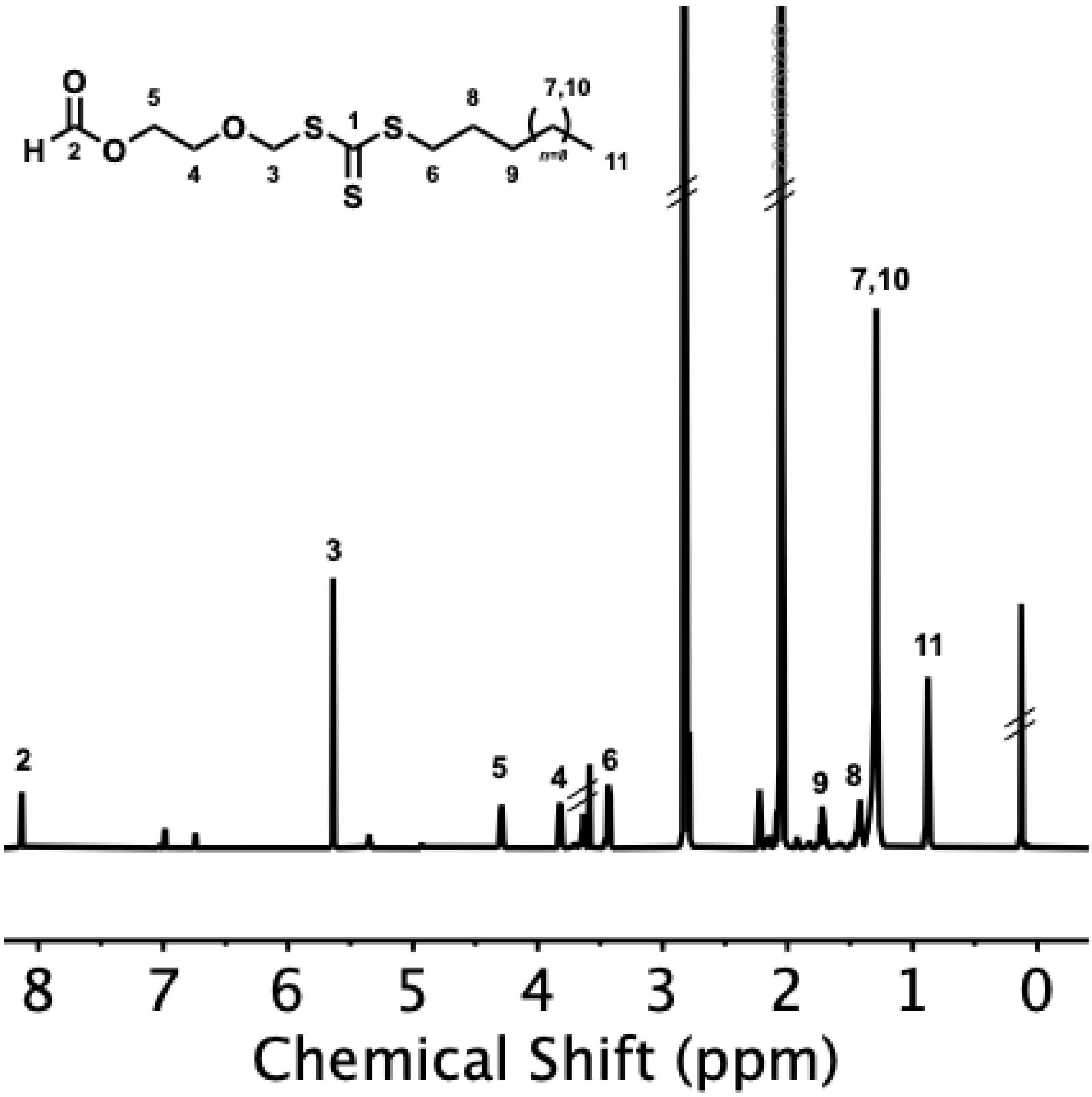

Figure S44. ^{1}H NMR of molecule **6** with indicated hydrogen atoms.

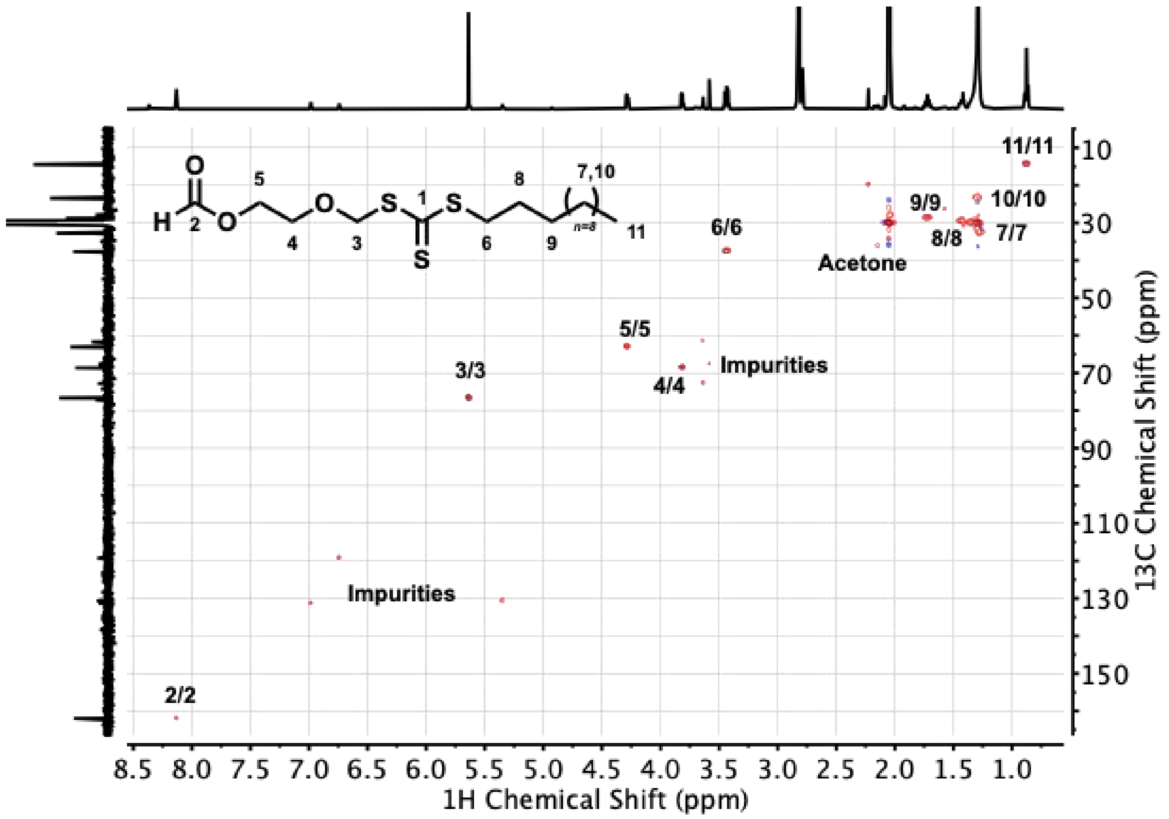

Figure S45. 2D NMR (HSQC) of molecule **6** with indicated H-C correlations.

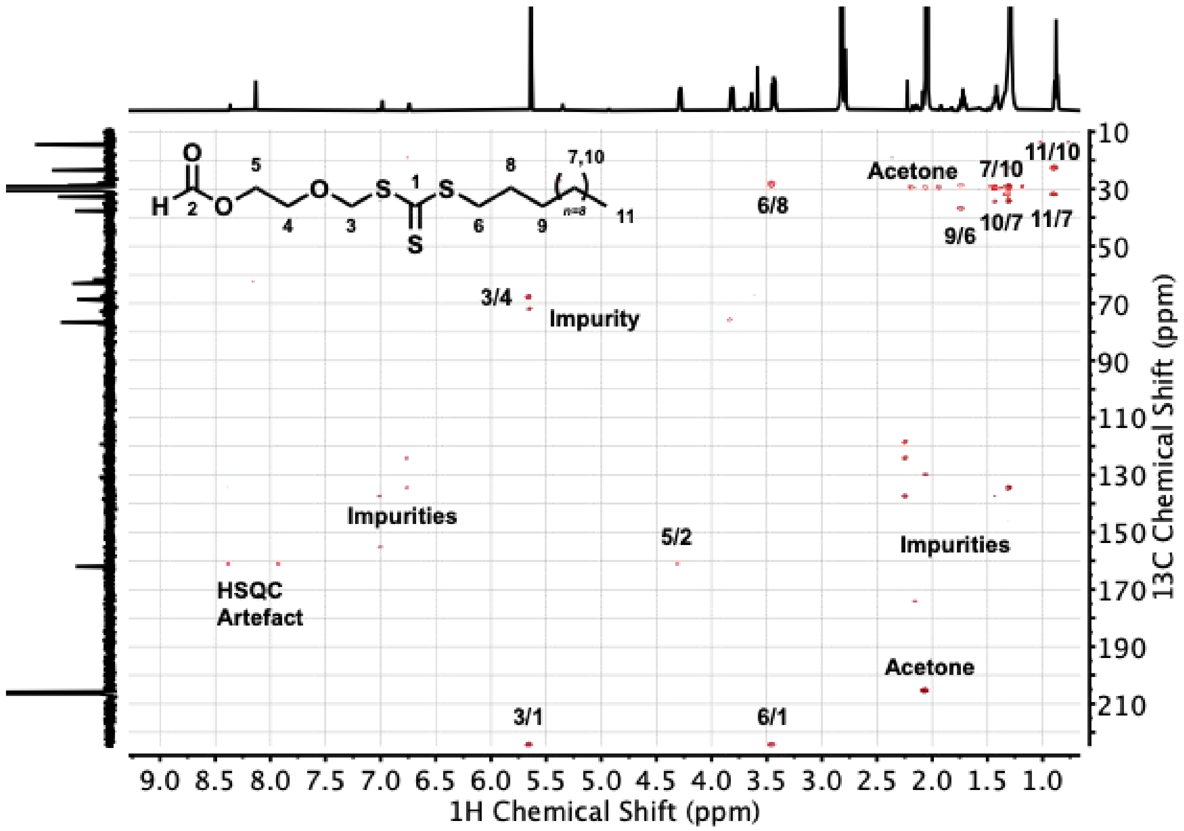

Figure S46. 2D NMR (HMBC) of molecule **6** with indicated H-C correlations.

6 2-((((dodecylthio)carbonothioyl)thio)methocy)ethyl formate

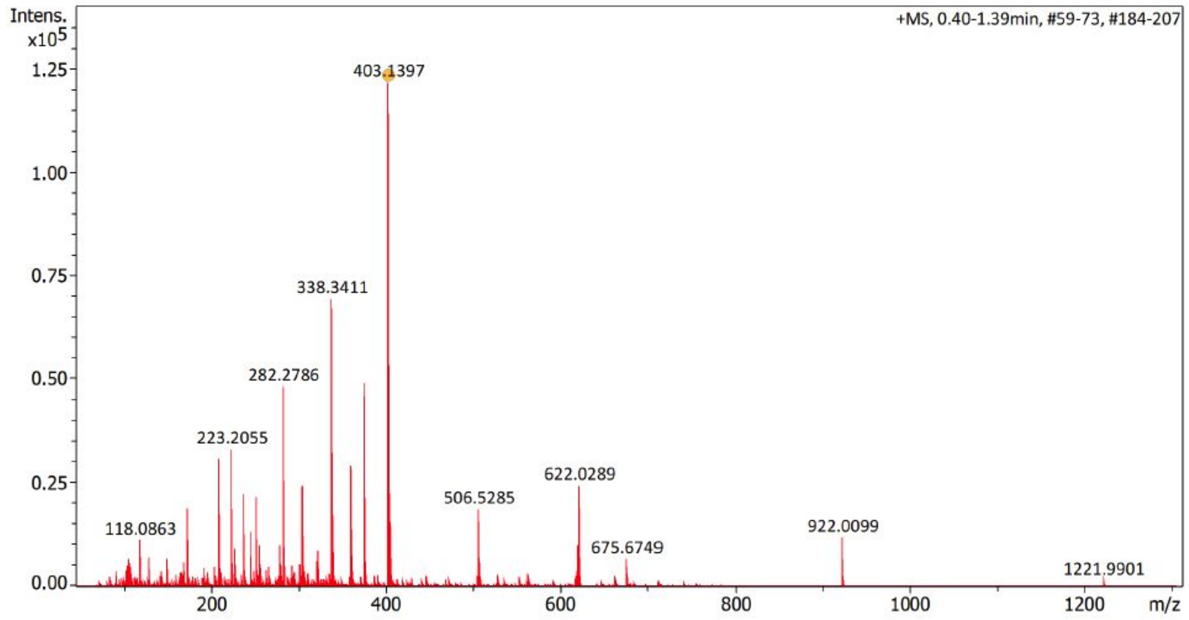

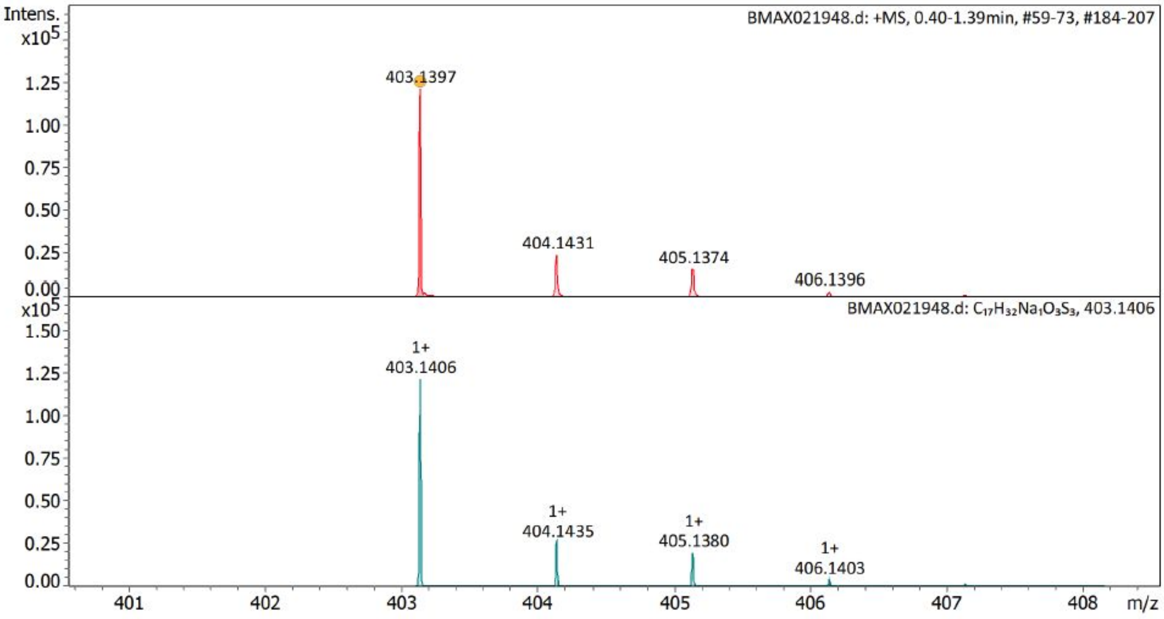

Figure S47. Full MS spectrum of molecule **6** (top), experimental isotope pattern (middle) and predicted isotope pattern (bottom).

6. Polymerization & Depolymerization of PBzMA-DTB

6.1 Synthesis of PBzMA-DTB

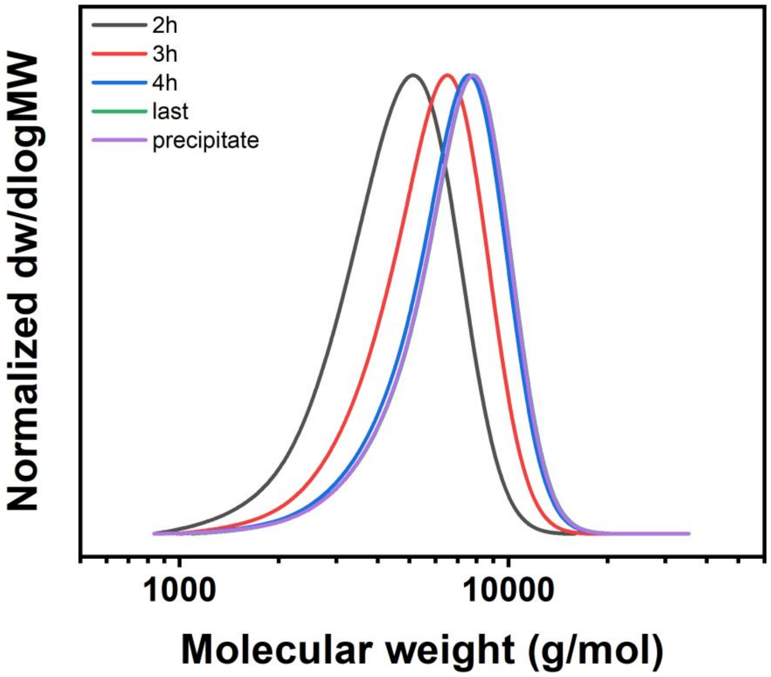

Figure S48. SEC trace of PBzMA synthesized via RAFT polymerization with DTB as the chain transfer agent ([MMA]:[CTA]:[AIBN] = 52:1:0.1).

Table S9. Characterization of PBzMA-DTB.

Entry	Monomer	[M]:[DTB]:[AIBN]	Time (h)	Conversion (%)	M_n^{theo} (g/mol)	M_n^{SEC} (g/mol)	*Đ*
1	BzMA	52:1:0.1	4h	70	6,600	6,300	1.16

6.2 Depolymerization of PBzMA-DTB

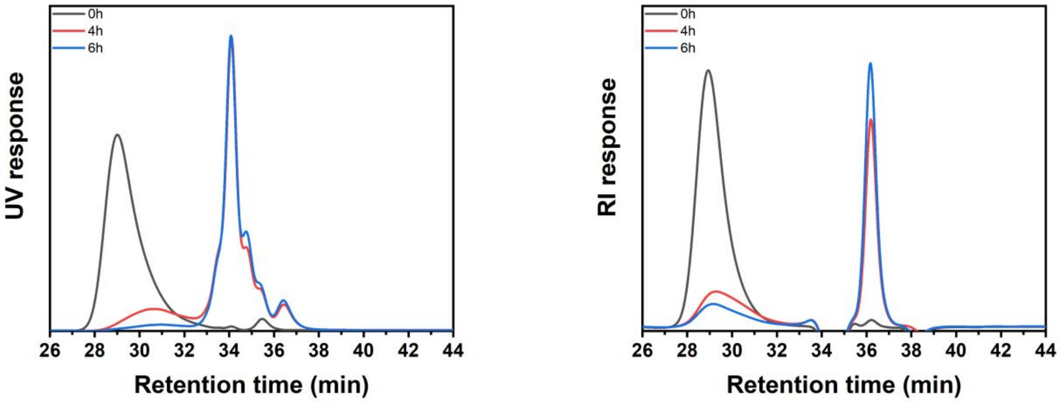

Figure S49. SEC trace of PBzMA-DTB depolymerization at 120 °C at 25 mM. UV-Vis (left) and RI (right).

Table S10. Characterization of PBzMA-DTB depolymerization.

Entry	Conc. (mM)	Temp. (°C)	Time (h)	Conversion (%)
1	25	120	6	85

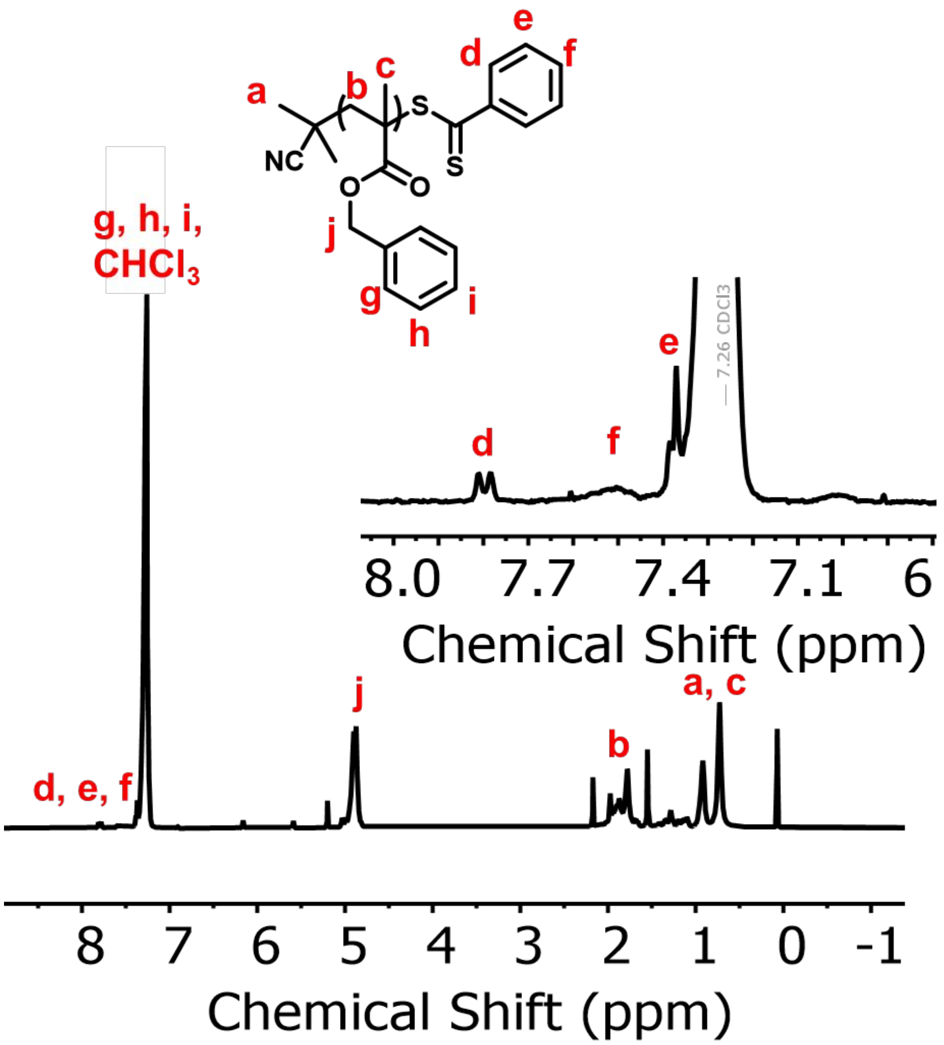

Figure S50. ^{1}H-NMR spectrum of purified PBzMA-DTB prior to depolymerization.

7 benzyl 4-cyano-2,4-dimethyl-2-((phenylcarbonothioyl)thio)pentanoate

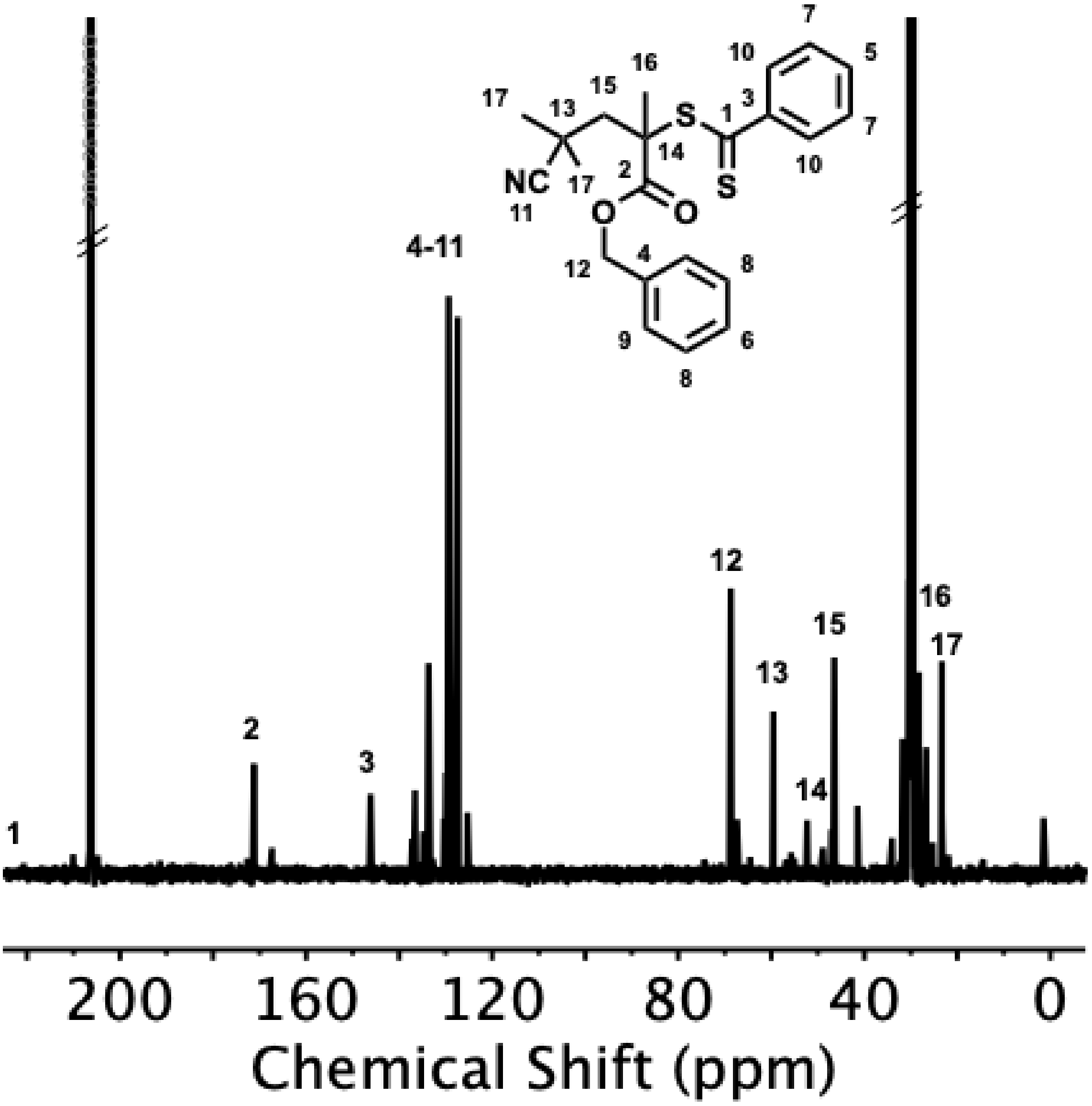

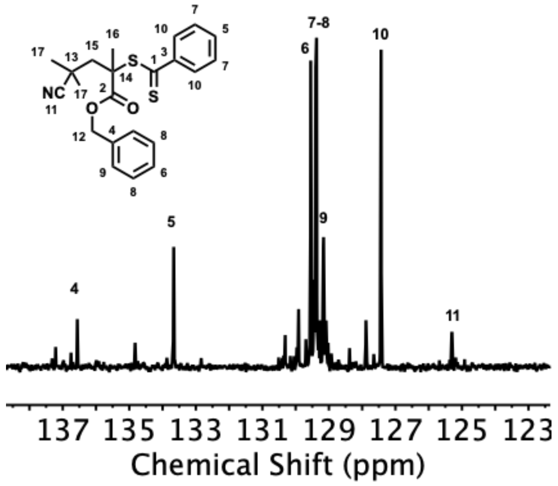

Figure S51. ^{13}C NMR of molecule **7** with indicated carbon atoms.

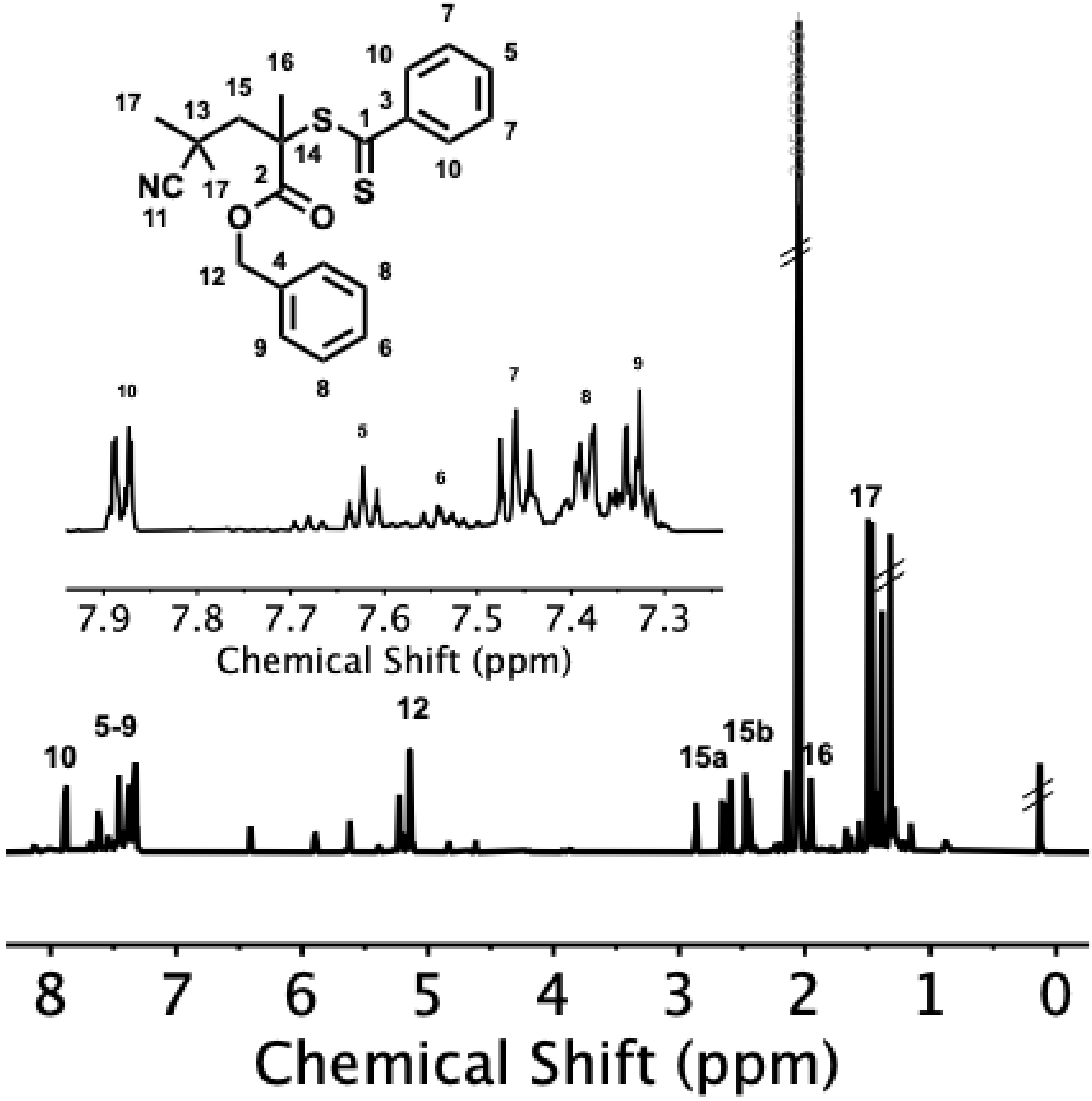

Figure S52. ^{1}H NMR of molecule **7** with indicated hydrogen atoms.

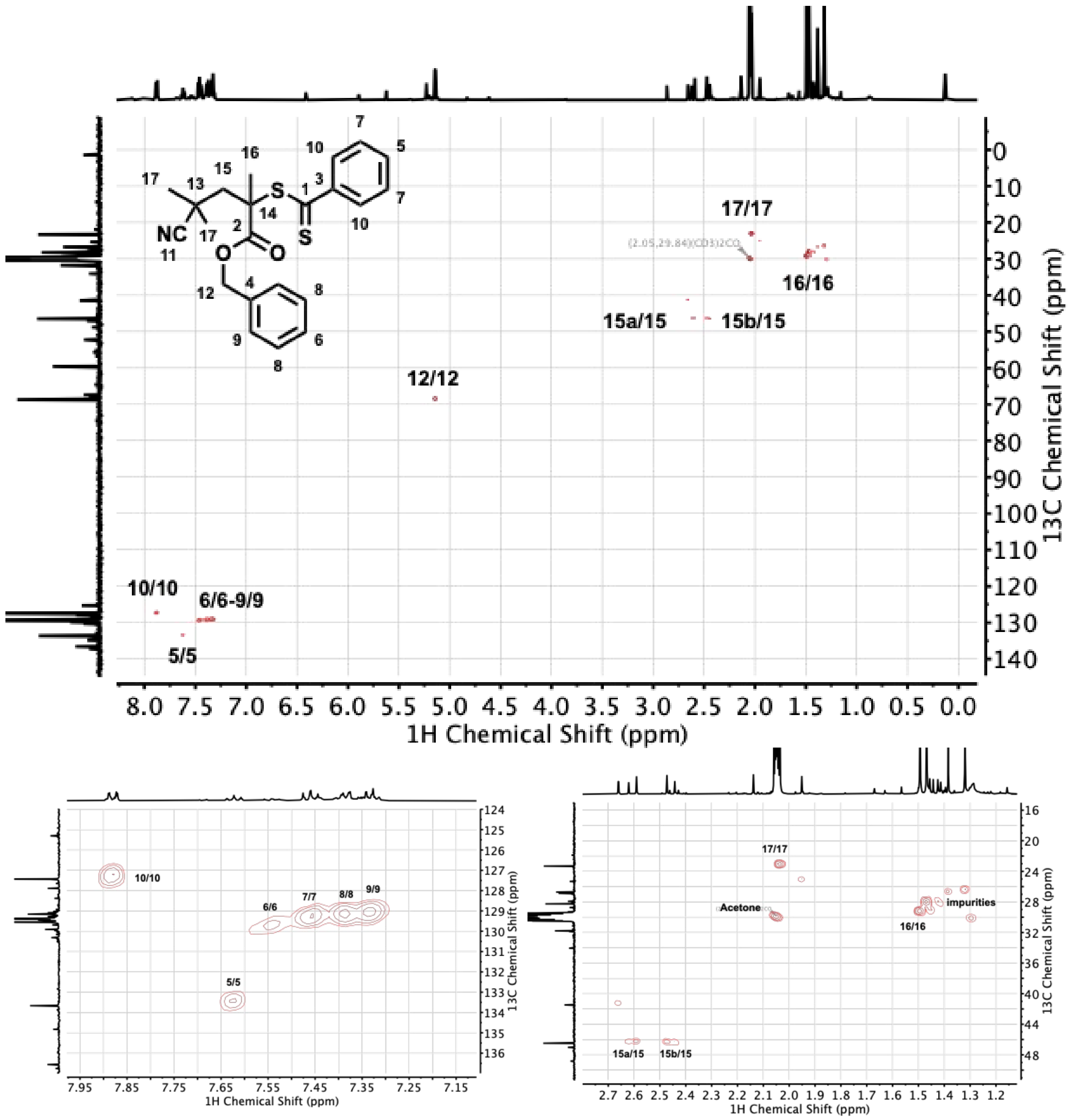

Figure S53. 2D NMR (HSQC) of molecule **7** with indicated H-C correlations.

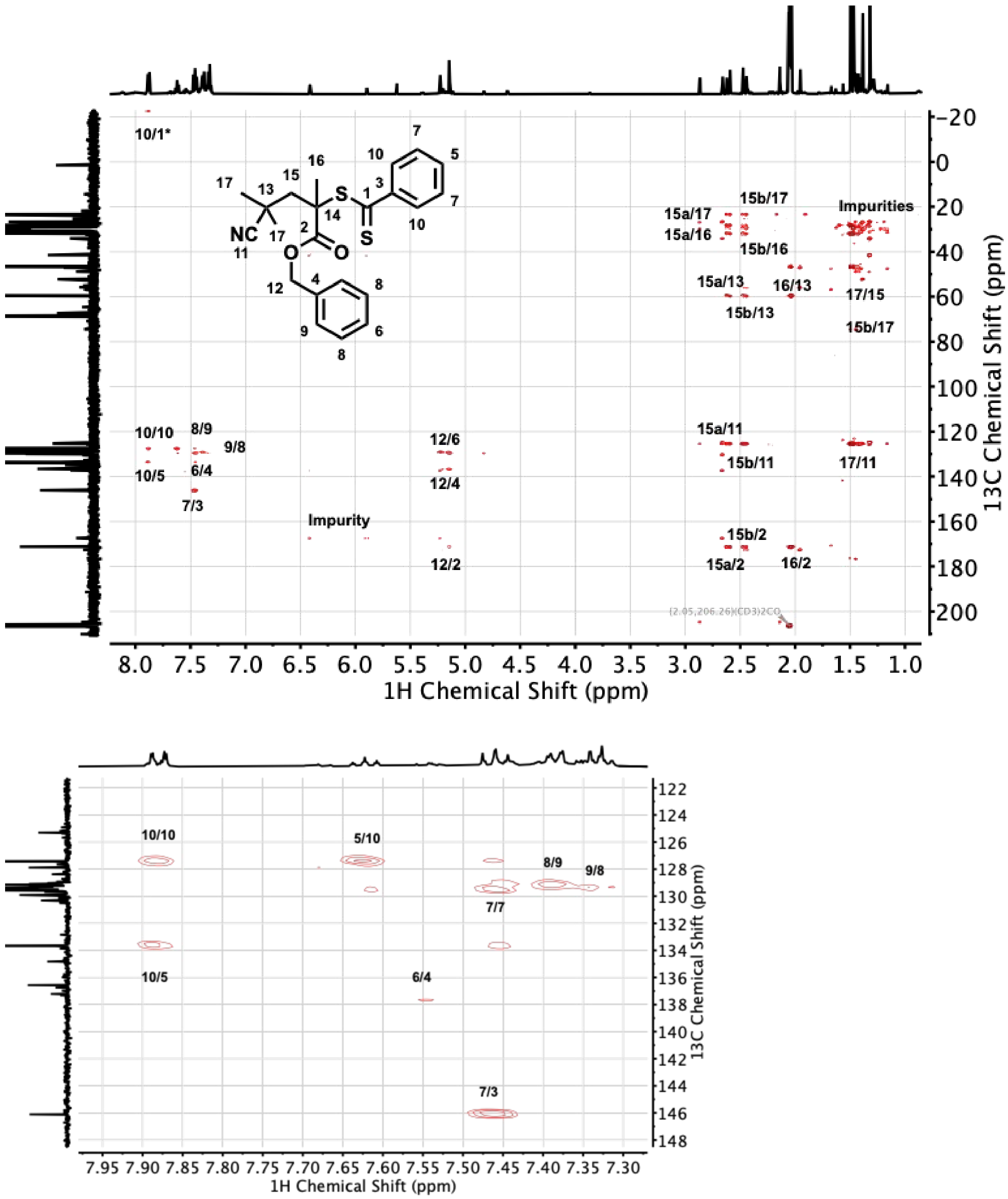

Figure S54. 2D NMR (HMBC) of molecule **7** with indicated H-C correlations.

7 benzyl 4-cyano-2,4-dimethyl-2-((phenylcarbonothioyl)thio)pentanoate

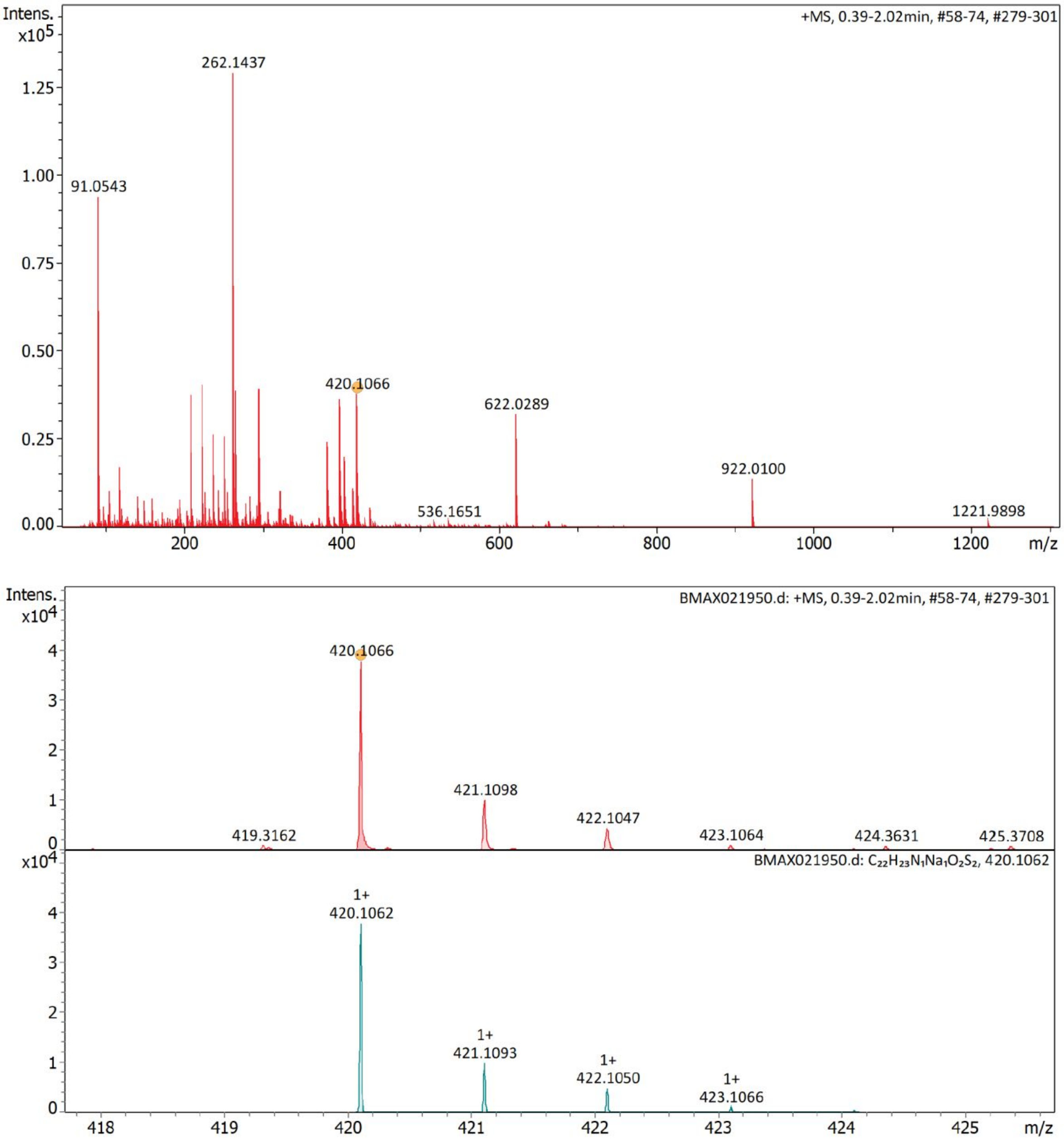

Figure S55. Full MS spectrum of molecule **7** (top), experimental isotope pattern (middle) and predicted isotope pattern (bottom).

7. Depolymerization of PMMA-DTB in *p*-xylene

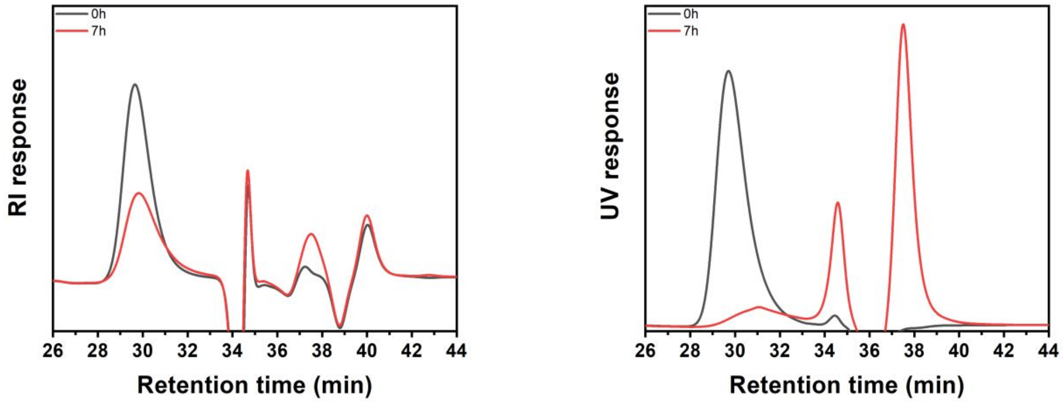

Figure S56. SEC trace of PMMA-DTB in *p*-xylene depolymerization at 120 °C at 25 mM. UV-Vis (left) and RI (right).

Table S11. Characterization of PMMA-TTC depolymerization.

Entry	Conc. (mM)	Temp. (°C)	Time (h)	Conversion (%)
1	25	120	6	39

<u>8</u> **4-methtylbenzyl benzodithioate**

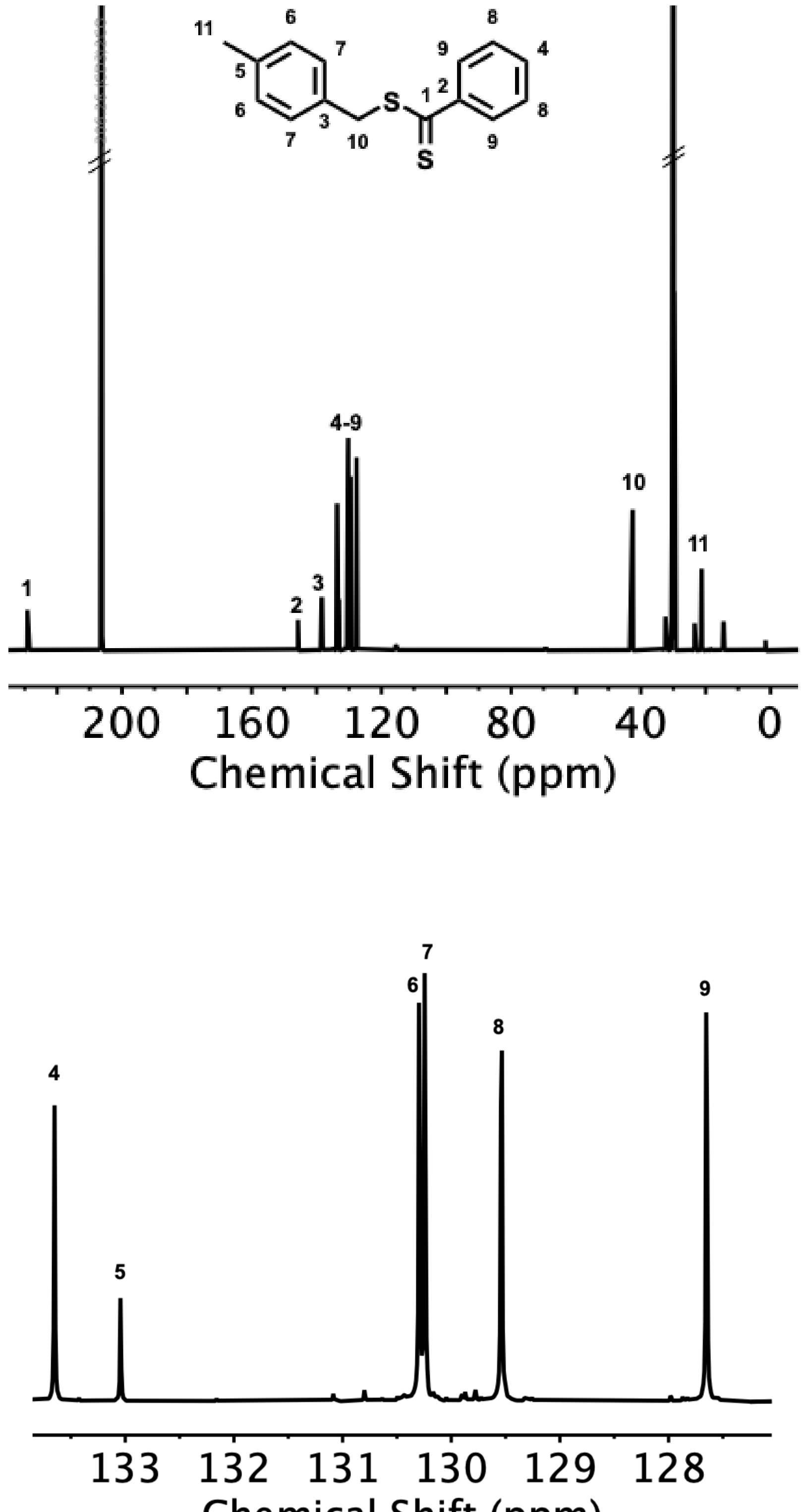

Figure S57. ^{13}C NMR of molecule **8** with indicated carbon atoms.

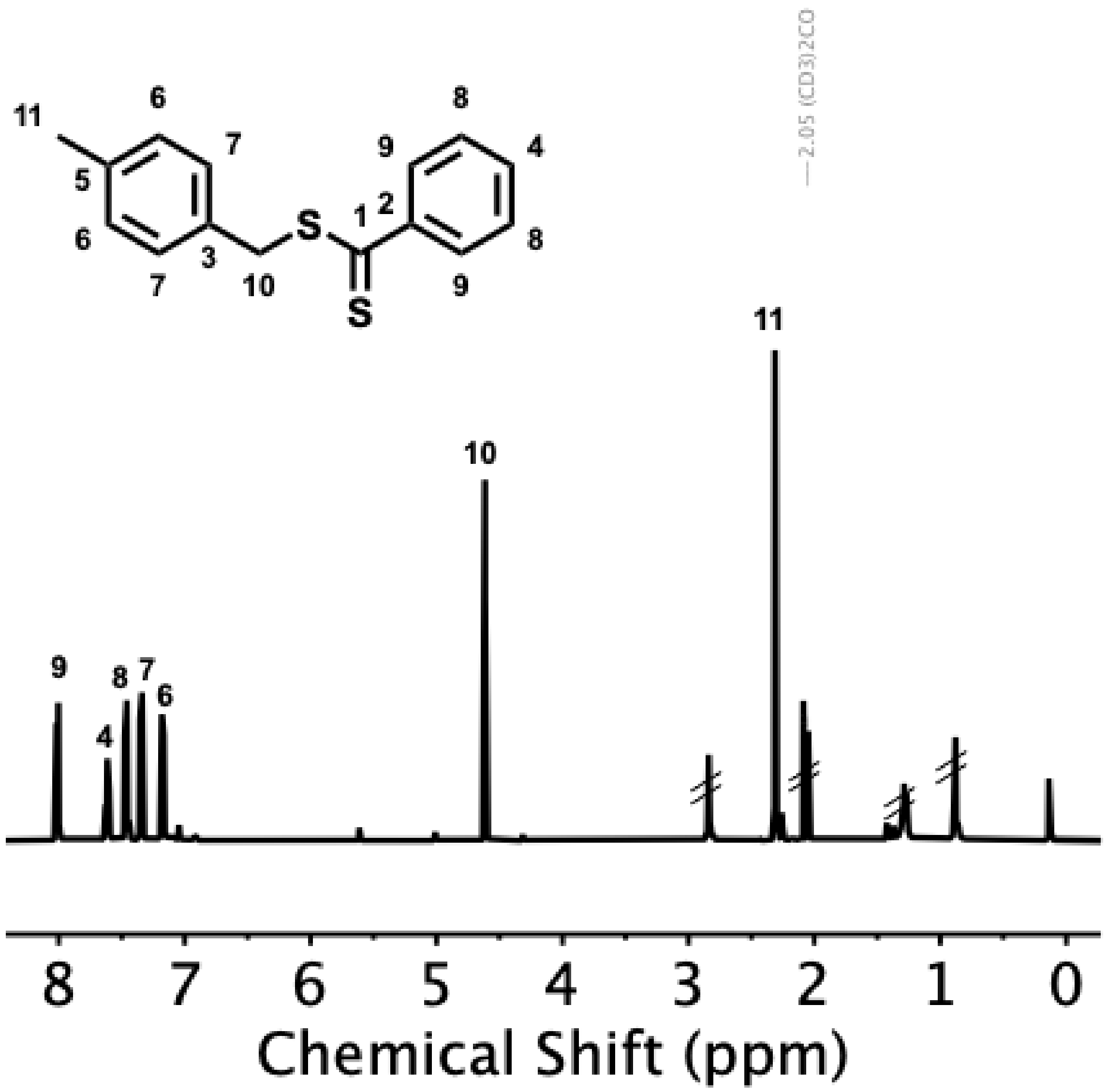

Figure S58. ^{1}H NMR of molecule **8** with indicated hydrogen atoms.

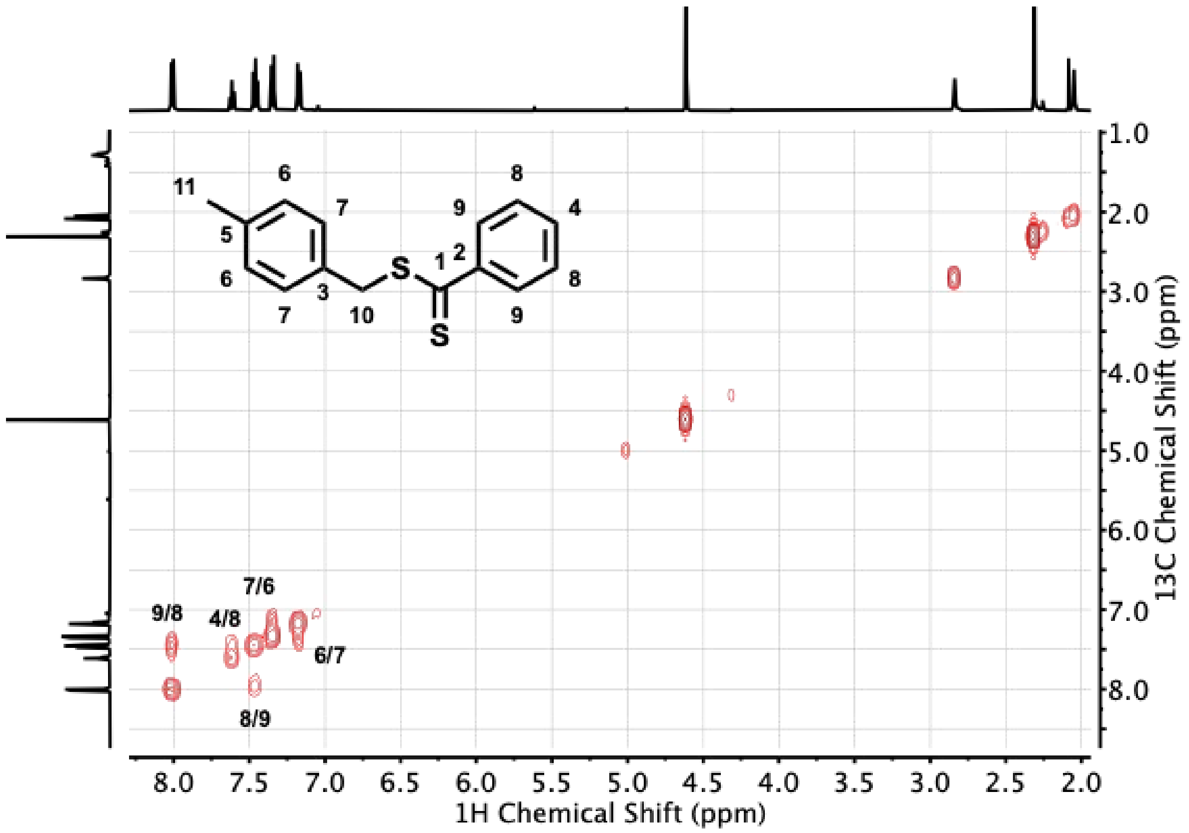

Figure S59. 2D NMR (COSY) of molecule **8** with indicated H-C correlations.

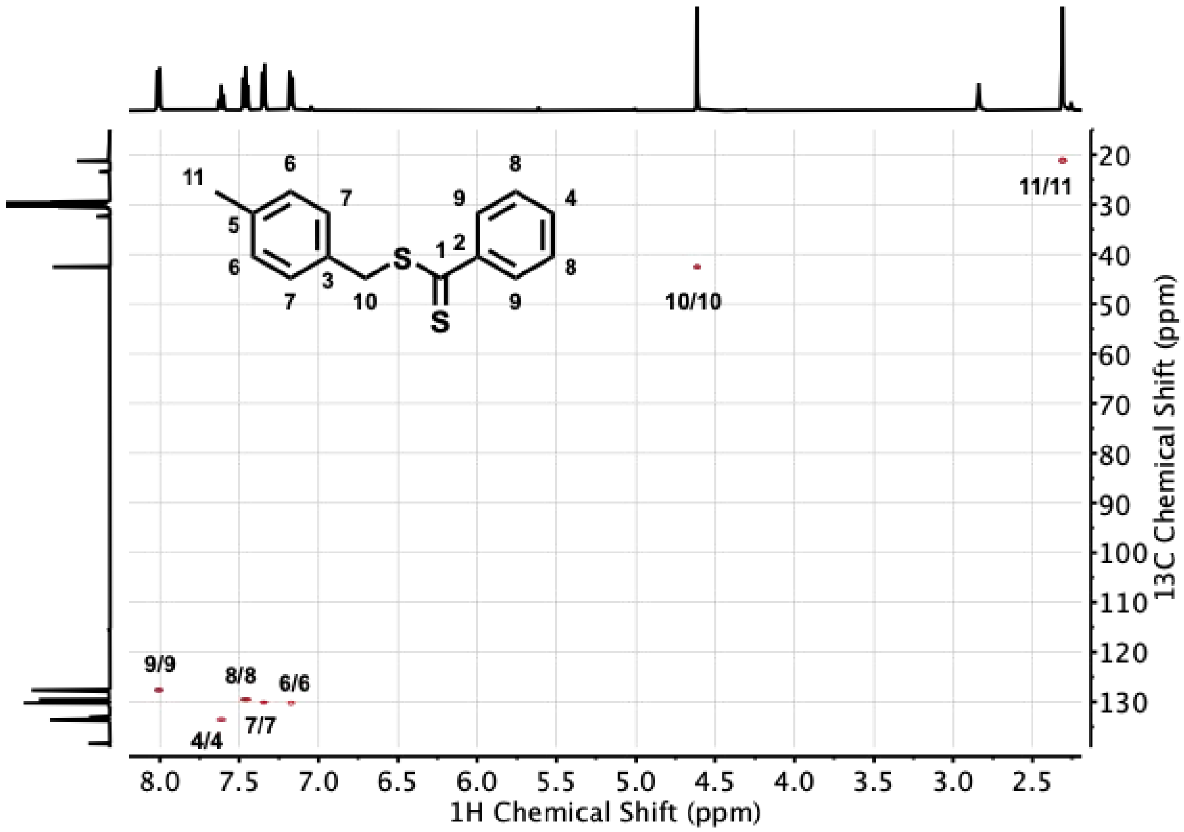

Figure S60. 2D NMR (HSQC) of molecule **8** with indicated H-C correlations.

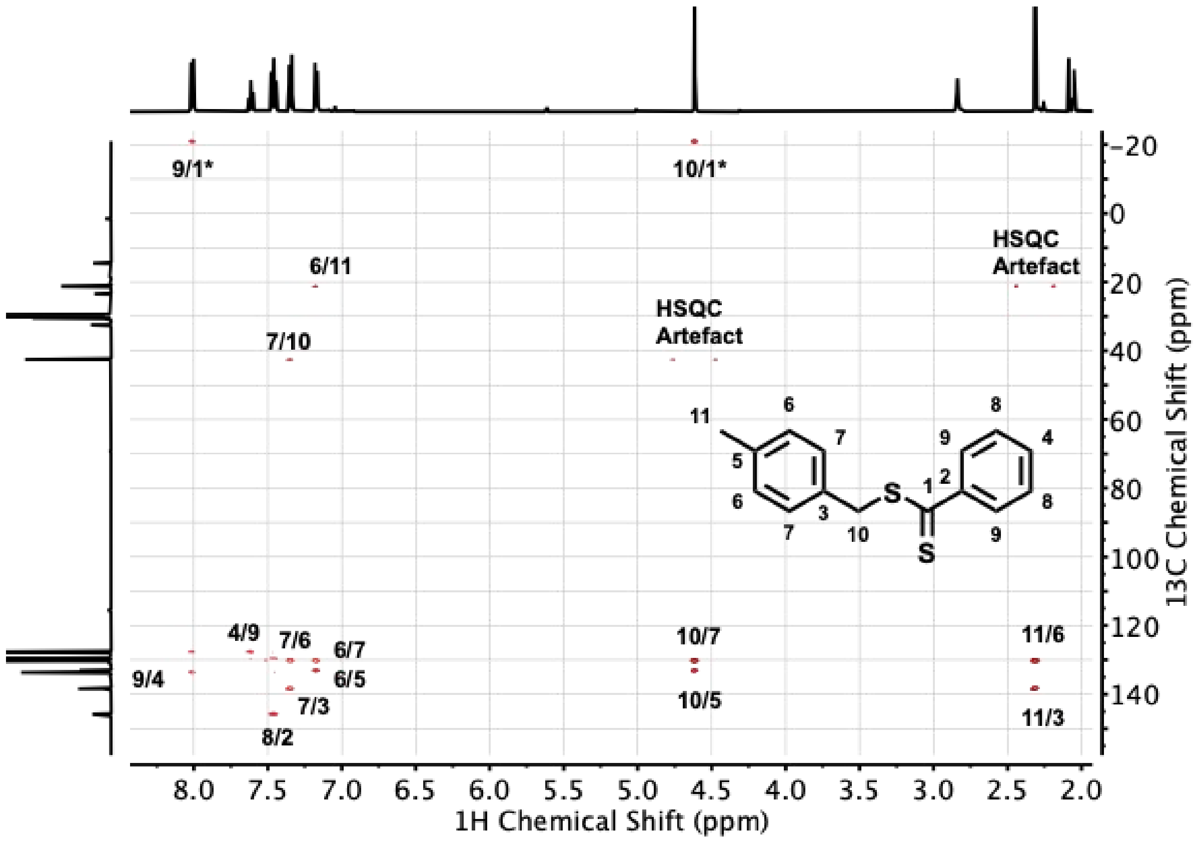

Figure S61. 2D NMR (HMBC) of molecule **8** with indicated H-C correlations.

8 4-methtylbenzyl benzodithioate

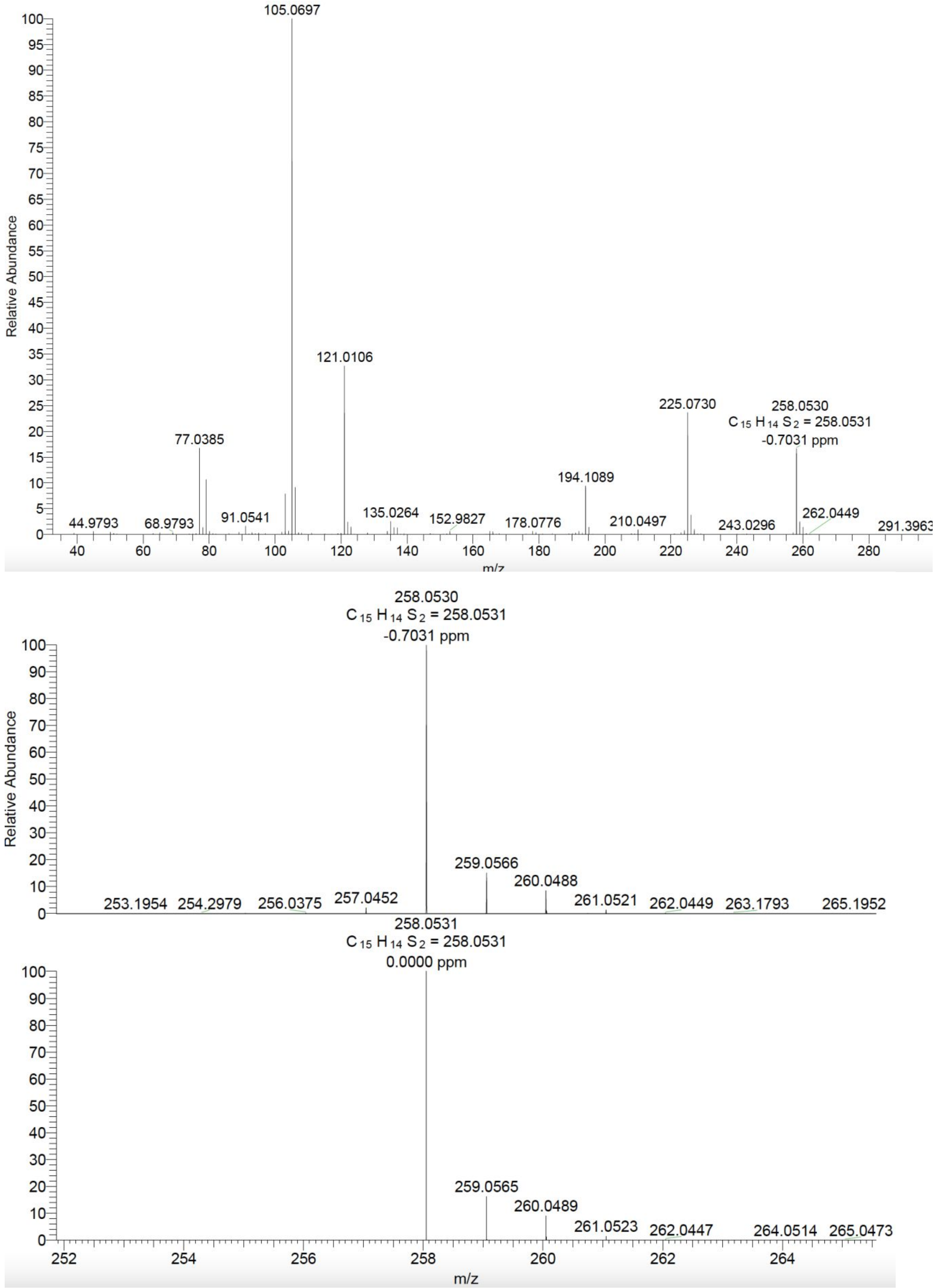

Figure S62. Full MS spectrum of molecule **8** (top), experimental isotope pattern (middle) and predicted isotope pattern (bottom).
